# Hydrogen Bonding Effect on the Oxygen Binding and Activation in Cobalt(III)-Peroxo Complexes

**DOI:** 10.1021/acs.inorgchem.2c04260

**Published:** 2023-01-19

**Authors:** Rob Bakker, Abhinav Bairagi, Mònica Rodríguez, Guilherme L. Tripodi, Aleksandr Y. Pereverzev, Jana Roithová

**Affiliations:** Institute for Molecules and Materials, Radboud University, Heyendaalseweg 135, 6525 AJ Nijmegen, The Netherlands

## Abstract

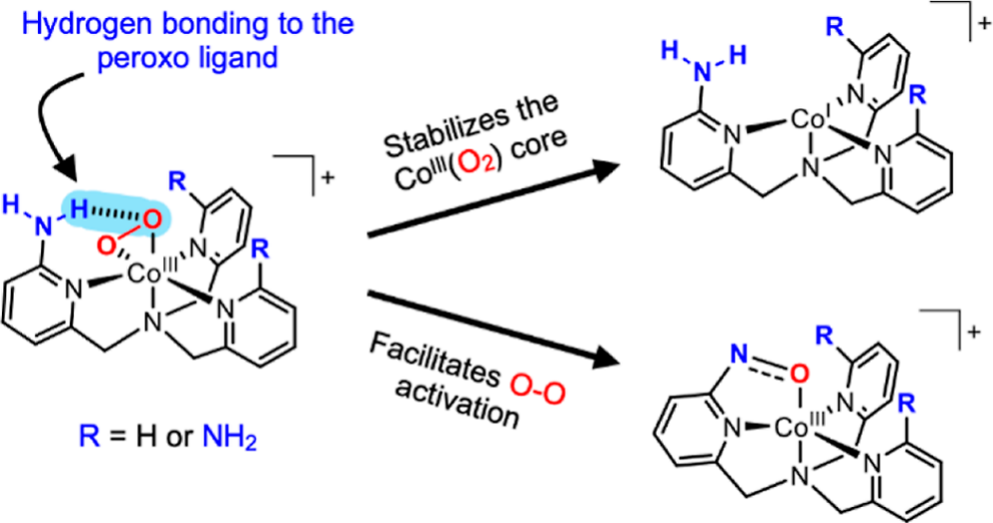

Cobalt(III)peroxo complexes serve as model metal complexes mediating oxygen activation. We report a systematic study of the effect of hydrogen bonding on the O_2_ binding energy and the O–O bond activation within the cobalt(III)peroxo complexes. To this end, we prepared a series of tris(pyridin-2-ylmethyl)amine-based cobalt(III)peroxo complexes having either none, one, two, or three amino groups in the secondary coordination sphere. The hydrogen bonding between the amino group(s) and the peroxo ligand was investigated within the isolated complexes in the gas phase using helium tagging infrared photodissociation spectroscopy, energy-resolved collision-induced dissociation experiments, and density functional theory. The results show that the hydrogen bonding stabilizes the cobalt(III)peroxo core, but the effect is only 10–20 kJ mol^–1^. Introducing the first amino group to the secondary coordination sphere has the largest stabilization effect; more amino groups do not change the results significantly. The amino group can transfer a hydrogen atom to the peroxo ligands, which results in the O–O bond cleavage. This process is thermodynamically favored over the O_2_ elimination but entropically disfavored.

## Abstract 2

Properties of cobalt(III)peroxo complexes with a variable number of the amino groups in the secondary coordination sphere were explored using mass spectrometry and ion spectroscopy. An increasing number of the amino groups in the secondary coordination sphere increases the O_2_ binding energy in the complexes and weakens the O−O bond. Hydrogen atom transfer from an amino group to the peroxy group opens a path to the O−O bond cleavage processes.

## Introduction

Hydrogen bonding is one of the dominant interactions that nature uses to control the structure and reactivity of proteins. Hydrogen bonding plays a vital role in the binding and activating molecular oxygen. On the one hand, it helps stabilize the dioxygen (O_2_) binding to iron-dependent proteins like hemoglobin and myoglobin.^1^ On the other hand, it helps drive the reactivity in O_2_ utilizing enzymes like oxidases and oxygenases.^2^ However, the exact molecular details of hydrogen-bonding assistance are challenging to study due to the complex dynamic behavior of the hydrogen bonds in the enzymes.^3^

**Figure 1:**
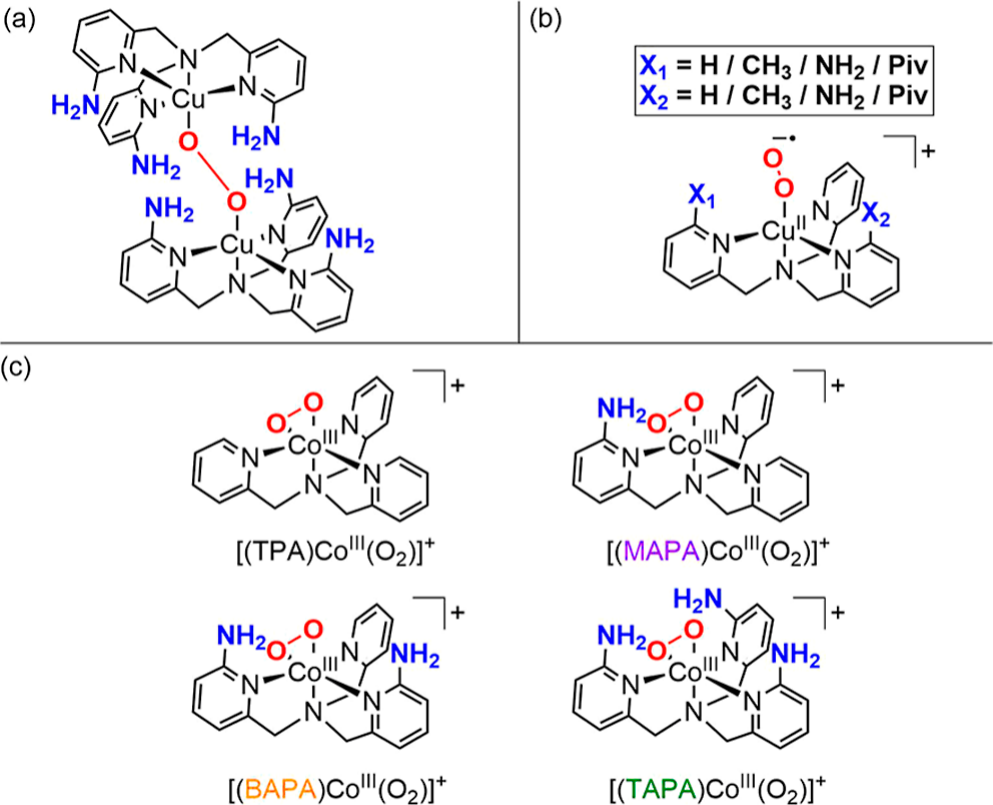
(a) Secondary coordination sphere effect on the copper-peroxo stability.^4^ (b) Secondary coordination sphere effect on copper-superoxo reactivity.^5^ (c) Complexes investigated here.

An elegant example for investigating the effect of hydrogen bonding on metal-dioxygen stabilization was reported previously by the group of Masuda.^4^ They studied the effect of hydrogen bonding on μ-peroxo dinuclear copper(II) complexes. They prepared a series of tripodal copper complexes bearing amino groups in the secondary coordination sphere, which could function as hydrogen bond donors (Figure 1a). They were able to establish a correlation between the thermal stability of the peroxo complexes and the number of hydrogen bond donors. The group of Karlin further expanded this field to mononuclear cupric superoxide complexes.^5^ The study showed a correlation between the reactivity of the cupric superoxide complex and the number of hydrogen bond donors (Figure 1b).

In recent years, cobalt(III)peroxo ([Co^III^(O_2_)]) complexes bearing the peroxo ligand (O_2_^2–^) are increasingly attractive as models for metal–O_2_ complexes. The [Co^III^(O_2_)] complexes have a high thermodynamic stability and a high kinetic stability, while they are electronically comparable to other metal-dioxygen species inspired by enzymes.^6,7^ Moreover, they are readily prepared at ambient conditions *via* the reaction between a cobalt(II) salt and hydrogen peroxide (H_2_O_2_).^8^

In this report, we propose tripodal [Co^III^(O_2_)] complexes as models to investigate the effect of hydrogen bonding on the metal-dioxygen binding and the oxygen–oxygen bond activation (Figure 1c). To this end, we employed the ligands developed by Masuda *et al.* and used advanced mass spectrometric (MS) techniques, such as infrared photodissociation (IRPD) spectroscopy and gas-phase dissociation studies, to obtain a direct structure–reactivity correlation.

## Results and Discussion

**Figure 2:**
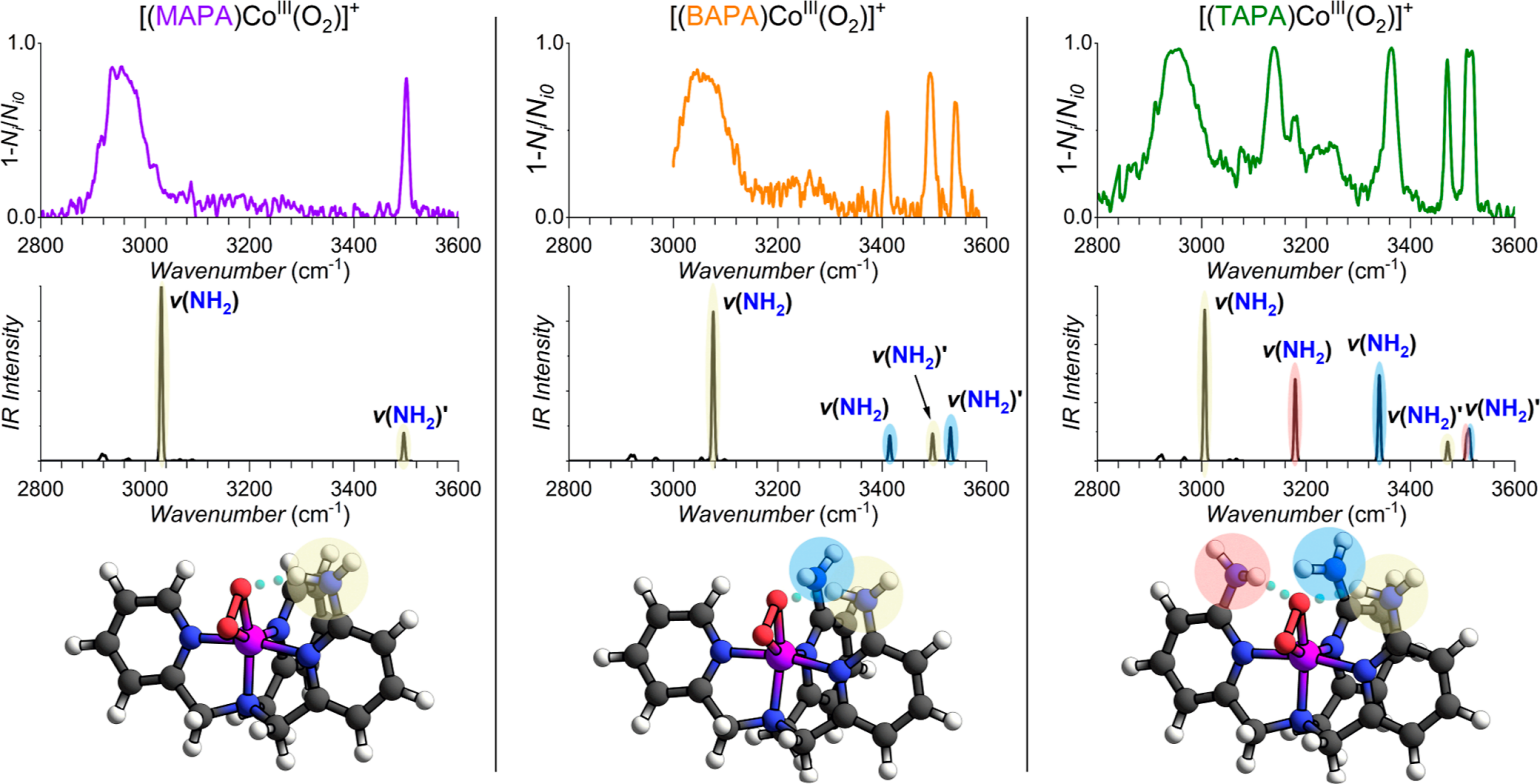
Helium tagging infrared photodissociation spectra of [(MAPA)Co^III^(O_2_)]^+^ (*m*/*z* = 396), [(BAPA)Co^III^(O_2_)]^+^ (*m*/*z* = 411), and [(TAPA)Co^III^(O_2_)]^+^ (*m*/*z* = 426) generated by electrospray ionization from acetonitrile solutions of the cobalt(II)nitrate complexes and H_2_O_2_ (top panels). Theoretical spectra (bottom panels) were calculated for the optimized structures at the B3LYP-D3/6-311G(2d,p) and scaled by a factor of 0.96. Symmetric stretching vibrations are denoted as *v*(NH_2_), and anti-symmetric stretching vibrations are denoted as *v*(NH_2_)′. Color code: blue, N; gray, C; pink, Co; red, O; and white, H.

To study the effect of hydrogen bonding on the O–O bond activation, we investigated the structure of complexes [(L)Co^III^(O_2_)]^+^ using helium tagging IRPD spectroscopy. The N–H stretching vibration region shows one broad red-shifted band reporting on the hydrogen bonding and the corresponding number of the other N–H bands. We have used the density functional theory (DFT) to interpret the origin of the bands. We found that all ions adopt a side-on (η^2^) mononuclear cobalt(III)peroxo geometry in analogy with other structures reported in the literature (Figure 2).^10−14^ Moreover, this side-on O_2_ ligand adopts a distorted axial geometry which puts all the amino groups within the common natural hydrogen bonding distance (2.7–3.3 Å) of the axial oxygen atom (Table S2 in Supporting Information).^15^ Hydrogen bonding leads to a red shift of the N–H stretch to lower wavenumbers, and the shift correlated with the bond strength of the hydrogen bonding. In agreement with the experiment, the DFT calculations predict that all three ionic complexes adopt one dominant hydrogen bond (Figure 2, yellow). The wavenumber of the corresponding N–H bond is shifted to 2953 cm^–1^ for (6-aminopyridin-2-ylmethyl)bis(pyridine-2-ylmethyl)amine (MAPA), 3056 cm^–1^ for bis(6-aminopyridin-2-ylmethyl) (pyridine-2-ylmethyl)amine (BAPA), and 2951 cm^–1^ for tris(6-aminopyridin-2-ylmethyl)amine (TAPA). In addition, TAPA adopts a second weaker hydrogen bond (Figure 2, red), leading to a shift to 3138 cm^–1^ of the corresponding N–H stretch. The vibration of the N–H bond of the amino group trans to the peroxo ligand (Figure 2, blue) is in the region typical for non-hydrogen bonded primary amino groups. These vibrations are at 3410 cm^–1^ for BAPA and 3363 cm^–1^ for TAPA.

**Figure 3:**
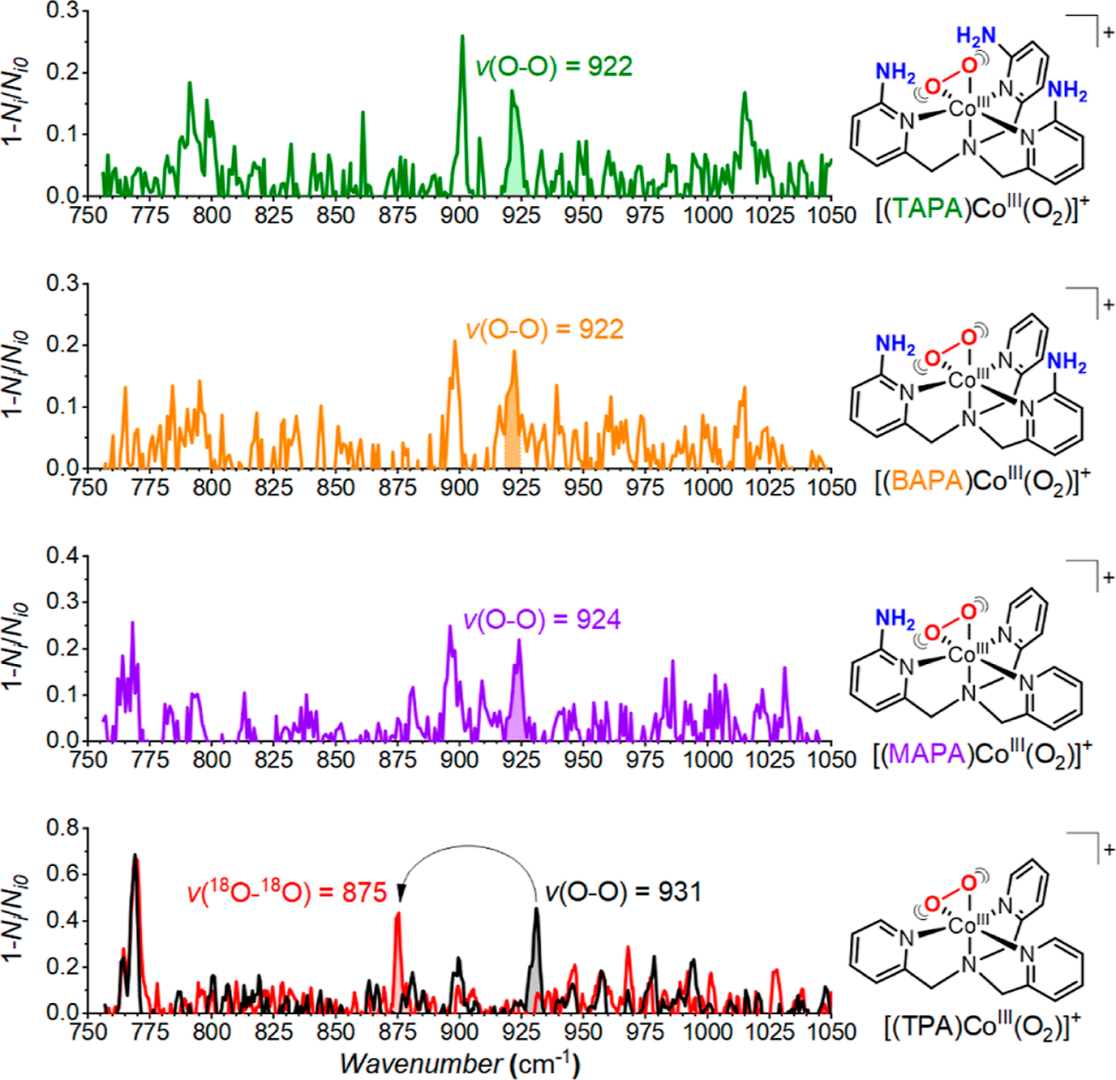
Helium tagging IRPD spectra of [(TPA)Co^III^(O_2_)]^+^ (*m*/*z* = 381), [(TPA)Co^III^(^18^O_2_)]^+^ (*m*/*z* = 385), [(MAPA)Co^III^(O_2_)]^+^ (*m*/*z* = 396), [(BAPA)Co^III^(O_2_)]^+^ (*m*/*z* = 411), and [(TAPA)Co^III^(O_2_)]^+^ (*m*/*z* = 426) generated by electrospray ionization from acetonitrile solutions of the cobalt(II)nitrate complexes and H_2_O_2_ in the O–O stretching vibration range. The O–O vibration was identified using ^18^O labeling of the peroxo ligand.

Next, we identified and investigated the O–O bond stretching vibration using IRPD and ^18^O labeling of the peroxo ligand (Figure 3). The hydrogen bonding should affect the electron density at the O–O group, and thereby, it should affect the observed O–O stretching vibration. As expected, the O–O stretching frequency decreases in the series TPA > MAPA > BAPA = TAPA (931, 924, 922, and 922 cm^–1^, respectively). The measured O–O stretching frequencies are in excellent agreement with the earlier reported side-on metal-peroxo species.^16,17^ Hence, the result confirms that the addition of NH_2_ groups to the tris(pyridin-2-ylmethyl)amine (TPA) ligand weakens the O–O bond. However, the effect is small. The introduction of the first amino group has the largest impact, the second amino group has a smaller effect, and the third amino group does not show any further effect on the O–O bond.

**Figure 4:**
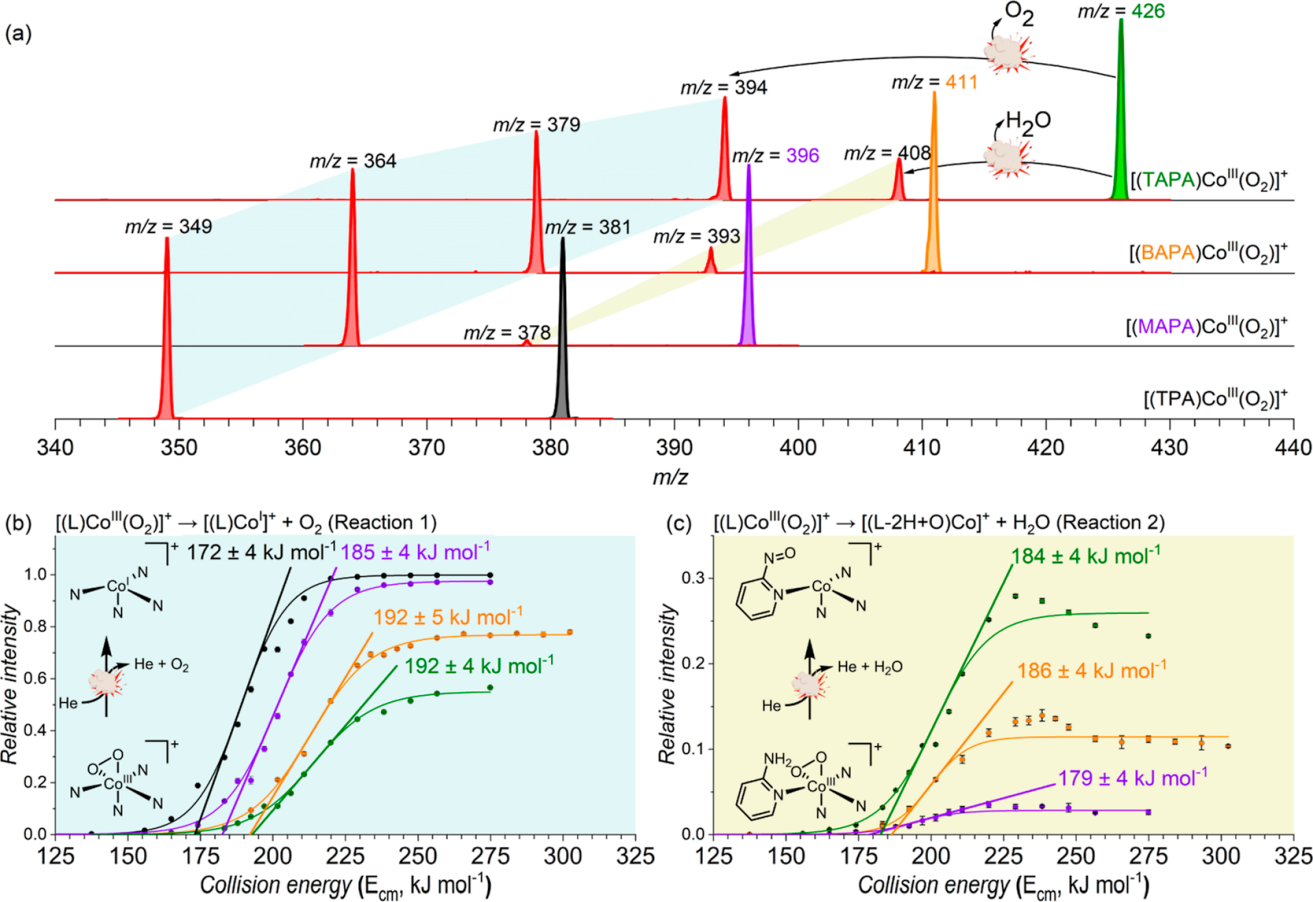
Collision-induced dissociation (CID) experiments of [(L)Co^III^(O_2_)]^+^ (L = TPA, MAPA, BAPA, and TAPA) generated by electrospray ionization from acetonitrile solutions of the cobalt(II)nitrate complexes and H_2_O_2_. (a) CID spectra of the complexes at *E*_col_ = 0 kJ mol^–1^ (green, orange, violet, and black) and *E*_col_ = 255 kJ mol^–1^ (red). (b,c) Energy-resolved CID experiments with mass-selected [(L)Co^III^(O_2_)]^+^. The graphs show the integrated total-ion-current normalized abundances of (b) [(L)Co]^+^ and (c) [(L-H2 + O)Co]^+^ as functions of the collision energy (see also Figure S19).

Next, we studied bond dissociation energies (BDEs) of O_2_ in the [(L)Co^III^(O_2_)]^+^ complexes using energy-resolved collision-induced dissociation (CID) experiments (Figure 4).^18^ These experiments allow us to control the energy supplied to the ions during their fragmentation and thereby derive the onset energy required for the fragmentation processes (see the Experimental Section). The [(TPA)Co^III^(O_2_)]^+^ complex fragments exclusively by the elimination of O_2_ (reaction 1, Figure 4b). The amino substituents at the other [(L)Co^III^(O_2_)]^+^ complexes open a second fragmentation path, leading to the elimination of H_2_O with concomitant formation of a nitroso moiety (reaction 2 in Figure 4b). The relative abundance of the H_2_O elimination increases with the number of NH_2_ substituents. This fragmentation suggests that the nearby amino groups mediate the O–O activation.

The BDEs of O_2_ increase in the series: [(TPA)Co^III^(O_2_)]^+^ (172 ± 4 kJ mol^–1^) < [(MAPA)Co^III^(O_2_)]^+^ (185 ± 4 kJ mol^–1^) < [(BAPA)Co^III^(O_2_)]^+^ (192 ± 5 kJ mol^–1^) = [(TAPA)Co^III^(O_2_)]^+^ (192 ± 4 kJ mol^–1^) (Figure 4b). Hence, the trend correlates well with the O–O stretching frequencies (Figure S24 in Supporting Information). The amino substituents stabilize the [Co^III^(O_2_)] core. The O–O bond cleavage path leading to the H_2_O elimination has about 6–8 kJ mol^–1^ smaller energy demand than the O_2_ elimination (Figure 4c). The abundance of the H_2_O elimination is much smaller than that of the direct O_2_ elimination, meaning that this channel is entropically hindered (the reactions proceed *via* tight transition structures). Roughly, the same energy demand but the increasing abundance of H_2_O elimination with the number of the NH_2_ substituents suggests that the initial hydrogen atom transfer from the NH_2_ group to the peroxo ligand is a bottleneck of the H_2_O elimination and that the increased abundance is based on the statistical grounds.

**Figure 5:**
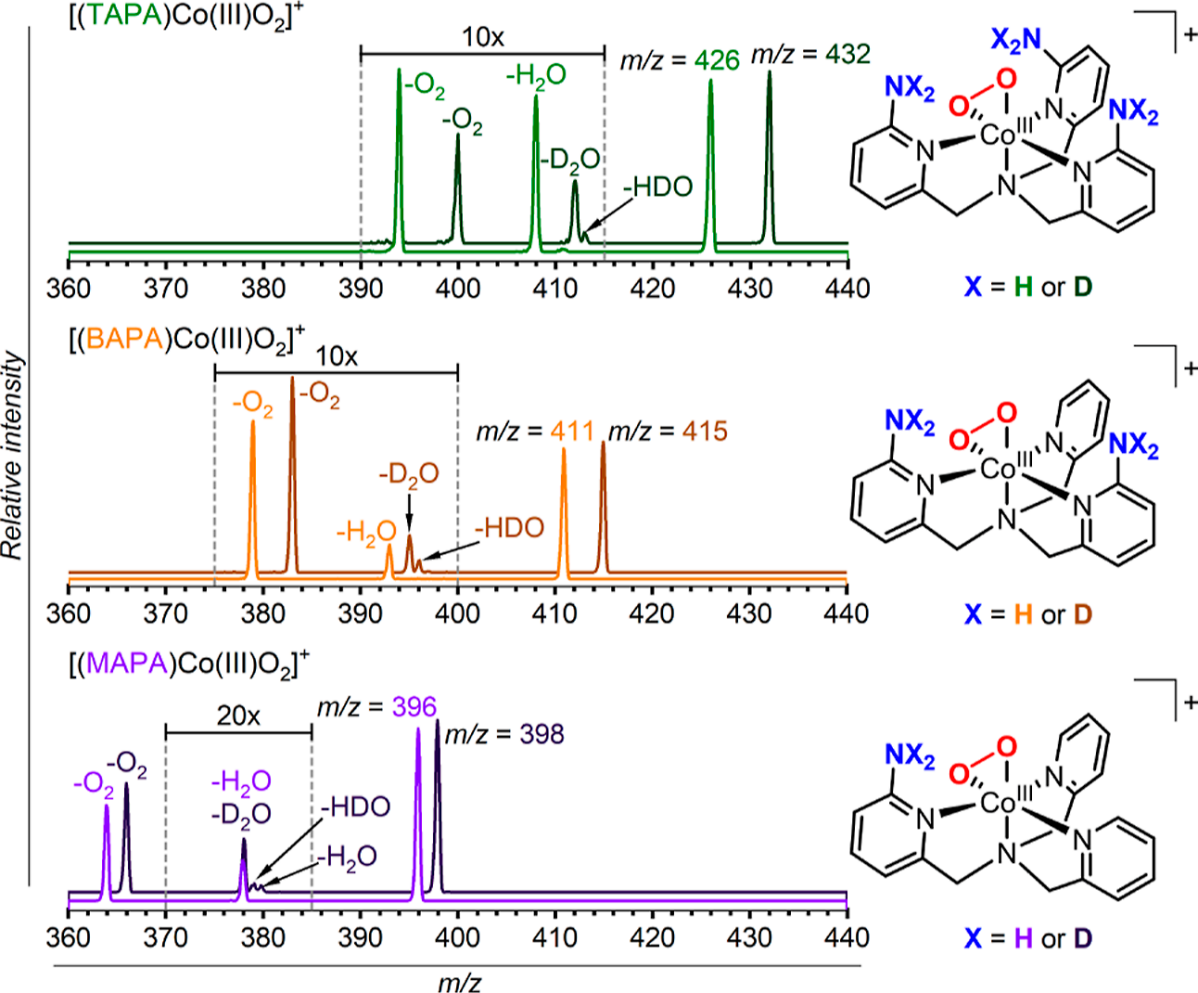
CID spectra of the depicted complexes, where X = H or D (MAPA, *E*_col_ ∼ 192 kJ mol^–1^; BAPA, *E*_col_ ∼ 200 kJ mol^–1^; and TAPA, *E*_col_ ∼ 200 kJ mol^–1^). The deuterated complexes were generated by electrospray ionization from acetonitrile/D_2_O (1:1 v/v) solutions of the cobalt(II)nitrate complexes and H_2_O_2_.

To gain a deeper insight into the process of the O–O bond cleavage and the subsequent H_2_O elimination, we repeated the experiments with deuterium-labeled complexes (Figure 5). To this end, we exchanged the labile H atoms at the amino groups with the D atoms (Figures S5–S16). The CID experiments clearly show that the dominant pathway leads to the D_2_O elimination from the labeled complexes. As expected, the amino groups provide the hydrogen atoms to assist the O–O bond cleavage and mediate the water elimination. We can also observe a minor hydrogen scrambling, leading to the elimination of HDO, which might indicate that the reactive intermediates along the water elimination pathway are sufficiently long-lived to partially scramble the hydrogen atoms in the ligand.

**Figure 6:**
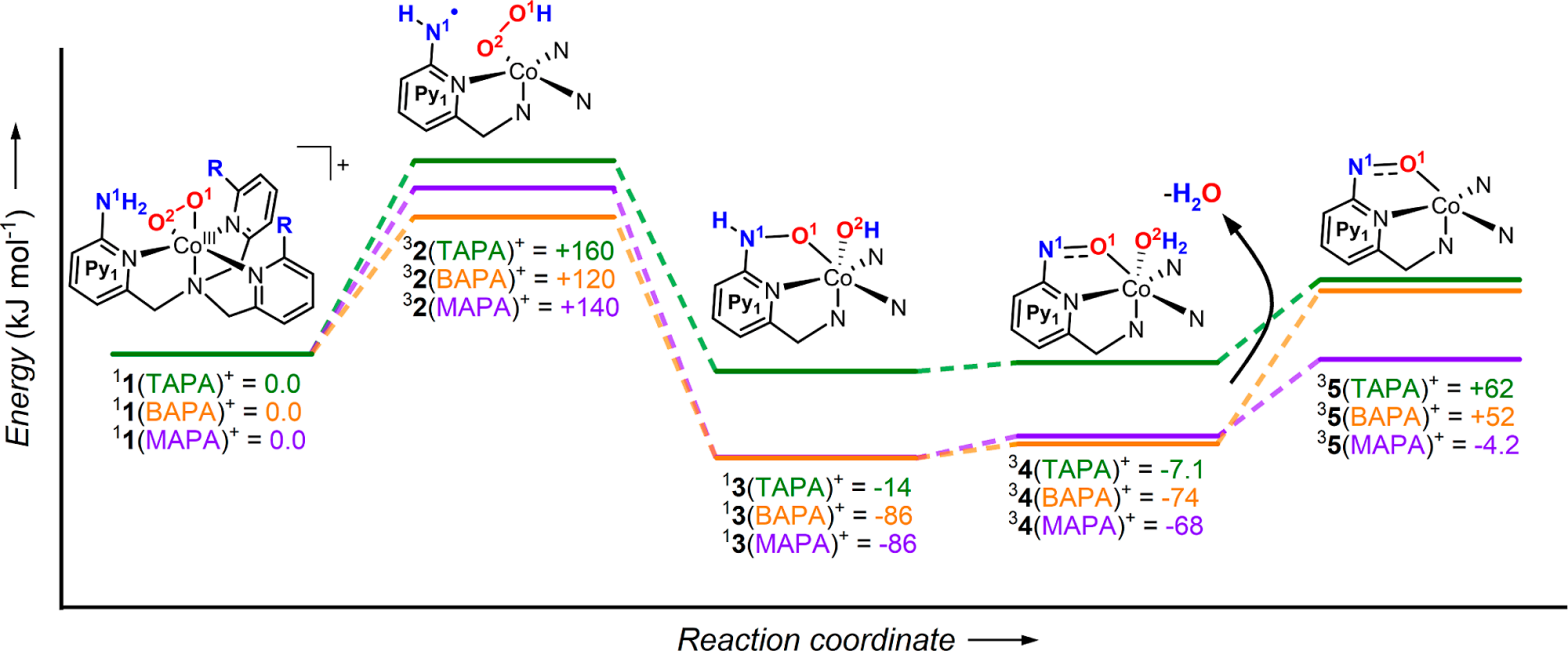
Potential energy surface for the O–O activation of [(L)Co^III^(O_2_)]^+^ [**1**(L)]. The structures were optimized at the B3LYP-D3/6-311G(2d,p) level. The energies refer to the 0 K and are in kJ mol^–1^.

Finally, we explored the O–O bond cleavage mechanism with DFT calculations (Figure 6). The starting cobalt(III)peroxo complex is in the singlet ground state (see the Supporting Information for all calculations and all spin states). The following hydrogen atom transfer from an NH_2_ group to the peroxo unit is associated with a spin-flip to the triplet state. The formed complex ^3^**2**(L) contains an aminyl radical, and the cobalt is reduced to the cobalt(II) state. According to the simple Mulliken population analysis, one unpaired electron partially resides at the aminyl radical site with delocalization toward the pyridine unit (Table 1). This complex represents the highest lying minimum on the explored path toward the H_2_O elimination. The formation of ^3^**2**(L) or the subsequent O–O bond cleavage will thus be the rate-determining step. The intermediates lie 125–165 kJ mol^–1^ higher in energy than the starting complexes. These energies are close to the energy demands determined experimentally for this reaction path, pointing toward late transition structures between ^1^**1**(L) and ^3^**2**(L). We did not attempt to localize the corresponding transition structures because they have inherently multiconfiguration characters, and the DFT values would be, in any case, only a rough estimate.

**Table 1:** Spin Densities Obtained from the Mulliken Population Analysis of the Triplet Intermediates in the O–O Bond Cleavage

complex	*s*_Co_	*s*_O1_	*s*_O2_	*s*_N1_	*s*_Py1_
**2**(MAPA)	1.24	0.13	0.05	0.38	0.19
**2**(BAPA)	1.11	0.08	0.03	0.49	0.30
**2**(TAPA)	1.11	0.05	0.02	0.51	0.32
**4**(MAPA)	0.97	0.31	0.02	0.41	0.21
**4**(BAPA)	0.98	0.26	0.02	0.47	0.19
**4**(TAPA)	0.99	0.24	0.02	0.49	0.19
**5**(MAPA)	1.06	0.25		0.36	0.26
**5**(BAPA)	1.08	0.21		0.39	0.25
**5**(TAPA)	1.11	0.19		0.40	0.23

The subsequent steps involve the O–O bond cleavage to form ^1^**3**(L) and the migration of the second hydrogen atom to form ^3^**4**(L). The final elimination of H_2_O leads to complex ^1^**5**(L), in which the amino substituent of one of the pyridine arms was transformed into the nitrosyl substituent. Such product formation is similar to the reaction of other metal peroxo complexes with aryl amines.^19^ The product complex has a triplet ground state with one unpaired electron localized at the nitrosyl ligand and the other at the cobalt center. We have also explored alternative pathways, but they either did not converge or lead to energetically higher intermediates (Scheme S2 in Supporting Information).

## Conclusions

The series of cobalt complexes with the TPA-based ligands being substituted with one, two, or three amino groups at the pyridine arms allowed us to investigate how hydrogen bonding affects the stability of cobalt(III)peroxo complexes and how the available labile hydrogen atoms can mediate the O–O bond cleavage. The complexes were investigated in the gas phase and were characterized by their IR photodissociation spectra. We found out that the hydrogen bonding stabilized the cobalt(III)peroxo core, but the effect was small. The BDEs of O_2_ increase in the series of complexes [(TPA)Co^III^(O_2_)]^+^ (172 ± 4 kJ mol^–1^) < [(MAPA)Co^III^(O_2_)]^+^ (185 ± 4 kJ mol^–1^) < [(BAPA)Co^III^(O_2_)]^+^ (192 ± 5 kJ mol^–1^) = [(TAPA)Co^III^(O_2_)]^+^ (192 ± 4 kJ mol^–1^). These values correlate well with the determined O–O bond stretching frequencies of the complexes. The O–O bond cleavage leading to the H_2_O elimination demands slightly smaller energy (6–8 kJ mol^–1^) than the elimination of O_2_, but it is entropically disfavored. Isotopic labeling studies and DFT calculations suggest that the reaction starts with hydrogen atom transfer from an amino substituent of the modified TPA ligands to the peroxo unit. This is the most energy-demanding step and determines the observed results. We observed the biggest effect upon introducing the first amino substituent. More of the substituents statistically favor more of the O–O bond cleavage path, but they do not change the underlying rationale. Future work should explore more acidic substituents in the vicinity of the reaction center. Especially, the protonated substituents should favor the O–O bond activation in this type of complexes. This would require a ligand design allowing protonation while keeping the cobalt complex stable.

## Experimental Section

The reagents and solvents were commercially obtained. Ligands, TPA, MAPA, BAPA, and TAPA, were prepared according to the procedures reported in the Supporting Information The cobalt(II)nitrate complex ([(L)Co^II^(NO_3_)_2_] 25 μM in acetonitrile) ligands, which are used to generate the peroxo complexes, were prepared by dissolving the ligands and Co(NO_3_)_2_·6H_2_O in acetonitrile. From this, the ionic complexes, denoted as [(L)Co^III^(O_2_)]^+^, were generated by an in-flow mixing of [(L)Co^II^(NO_3_)_2_] (25 μM in acetonitrile) and H_2_O_2_ [3.5%, (v/v) in acetonitrile] prior to the injection to the mass spectrometer.^20^ The electrospray ionization mass spectrometry (ESI-MS) analysis showed signals of ions corresponding to the peroxo complexes [(L)Co^III^(O_2_)]^+^ along with intense signals of [(L)Co^II^(NO_3_)]^+^, [(L)Co^II^(Cl)]^+^, [(L)Co^II^(OH)]^+^, and [(L-H)Co^III^(OH)]^+^ (Figures S3–S16 in the Supporting Information).

The MS experiments were performed with a Finnigan LCQ XP mass spectrometer equipped with an ESI source. The conditions were as follows: capillary temperature 200 °C, spray voltage 4.5 kV, capillary voltage 0 V, and tube lens offset 20 V. The collision energy was calibrated using Schröder’s method^21^ using the dissociation energies of known benzylpyridinium- and benzhydrylpyridinium thermometer ions (Table S1, Figures S17 and S18).^22^ The energy-resolved CID experiments were performed with mass-selected [(L)Co^III^(O_2_)]^+^ complexes (Figures S4, S6, S10, S14) in the full range of collision energies (Figure 4). The relative intensities of the parent and the fragment ions were plotted as a function of the collision energy (Figure 4b,c). The fragmentation threshold (the BDE) was determined as an extrapolation of a tangent of the sigmoidal fits of the experimental data to the zero intensity.

IRPD spectra were recorded with an ISORI instrument (Figure S20 in Supporting Information).^23^ In short, the [(L)Co^III^(O_2_)]^+^ complexes were mass-selected and guided to a helium-cooled ion trap (∼3K). Trapped ions formed weakly bound complexes between [(L)Co^III^(O_2_)]^+^ and helium atoms. The complexes were irradiated with a tuneable IR light (OPO/OPA system from LaserVision). If a helium-tagged complex absorbs an IR photon (v_i_), the vibrational excitation results in the helium elimination. The depletion of the helium complexes (1 – *N*(v_i_)/*N*_0_) correlates with the IR absorption intensity at v_i_.

## Supporting Information Available

Ligand synthesis; ^1^H and ^13^C NMR spectra, further details on MS methods; mass spectra; DFT calculations; and optimized *XYZ* coordinates (PDF)
