# Supplementary material for: Hydrogen Bonding Effect on the Oxygen Binding and Activation in Cobalt(III)-Peroxo Complexes
Source: Inorg Chem. 2023 Jan 19;62(4):1728–34. doi: 10.1021/acs.inorgchem.2c04260 (PMC9890563; doi:10.1021/acs.inorgchem.2c04260)
Supplement: Supplementary file 1 — ic2c04260_si_001.pdf [file ic2c04260_si_001.pdf]

Supporting Information

Hydrogen Bonding Effect on the Oxygen Binding and Activation in Cobalt(III)-peroxo Complexes

Rob Bakker, Abhinav Bairagi, Mònica Rodríguez, Guilherme L. Tripodi, Aleksandr Y. Pereverzev, Jana Roithová*

Institute for Molecules and Materials, Radboud University, Heyendaalseweg 135, 6525 AJ Nijmegen, The Netherlands

Table of Contents

General information S2

Synthesis and characterization of ligands S2

Mass spectrometric studies S7

Energy resolved collision induced dissociation experiments S15

Helium tagged photodissociation experiments and spectra S17

Density functional theory calculations S21

NMR Spectra S22

XYZ Coordinates S28

References S64

General information

All reagents and solvents were commercially obtained and used as received. All preparation and manipulations were carried out under normal conditions, unless noted otherwise. All the solvent and general reagents used in this synthesis were bought from commercial sources and were used as received. Dichloromethane(>99%, Fisher scientific), ethyl acetate (>99.8%, Fisher scientific), acetonitrile (>99.9%, HPLC grade Fisher scientific) were used. Methanol, heptane and chloroform were also received from Fisher scientific. Ammonia solution (7 N in methanol) was purchased from Sigma-Aldrich (Merck Life sciences N.V.). 6-Aminopicolinic acid methyl ester was purchased from Fluorochem EU. Phosphorus tribromide (99%) was purchased from Fisher scientific. Trimethylacetyl chloride (99%), Sodium iodide (acs reagent, >=99.5%), Sodium borohydride, and sodium methoxide, anhydrous sodium sulfate and magnesium sulfate were purchased from Sigma-Aldrich (Merck Life sciences N.V.). NMR spectra were recorded at 25 °C on a Bruker 500 MHz Avance III (1H NMR -500 MHz, 13C NMR -126 MHz) and on a 400 MHz Avance III (1H NMR-400 MHz, 13C NMR-101 MHz) spectrometer. The NMR spectra were referenced to the deuterated solvent ($CDCl_3$ 1H δ 7.26, 13C δ 77.16 and DMSO-D6 1H δ 2.5, 13C δ 39.52). Mass spectra were collected at Thermo scientific LTQ XL mass spectrometer with an electrospray source.

Synthesis and characterization of ligands

The ligands MAPA, BAPA, and TAPA were synthesized from the common precursor as shown in Scheme S1.

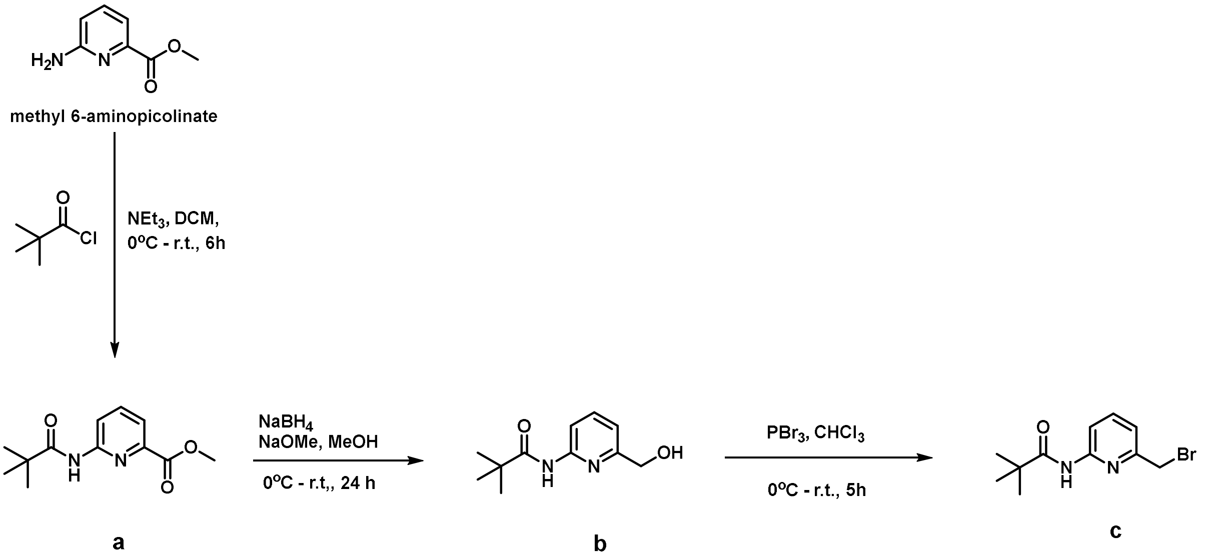

Scheme S1. Synthesis of the common precursor of MAPA, BAPA and TAPA.

Methyl-6-pivalamidopicolinate (a):

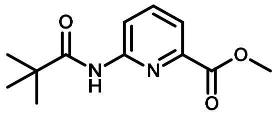

Methyl-6-aminopicolinate (4.0 g, 0.026 mol, 1eq.) was dissolved in 70 mL DCM, and (3.98 mL, 0.0286 mmol, 1.1 eq.) of trimethylamine was added to the mixture. While cooling on ice bath, (3.50 mL, 0.0286 mmol 1.1 eq.) of pivaloyl chloride was added to the mixture dropwise. After that, the solution was left for stirring for 6 hrs. Then the white ppt. was filtered, and filtrate was collected. The organic layers were washed with brine and dried over anhydrous NaSO4. The organic solvent was evaporated to yield off-white solid. The product was purified using ethyl acetate (10%)/heptane solvent system on silica gel column. (~ 5.5 g, 90%)

^{1}**H NMR** (400 MHz, $CDCl_3$) δ 8.48 – 8.40 (m, 1H), 8.14 (s, 1H), 7.84 – 7.74 (m, 2H), 3.94 (s, 3H), 1.28 (s, 9H).

^{13}**C NMR** (101 MHz, $CDCl_3$) δ 177.18, 164.97, 151.61, 145.62, 139.04, 120.82, 117.59, 52.65, 39.69, 27.21(3C).

MF: C25H33N7 (431.59 g/mol)

ESI-MS(M/Z):m/z = 384.08 [M+H]

N-(6-(hydroxymethyl)pyridin-2-yl)pivalamide (b):

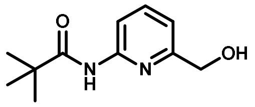

A modified reported procedure was used to synthesize b. [1] Methyl-6-pivalamidopicolinate (5.0 g, 0.021 mol, 1 eq.) was dissolved in 20 mL methanol with 8 mol% (~ 0.1 g, 0.00185 mol) sodium methoxide in a round bottom flask on an ice bath. Then, (3g, 0.079 mol) NaBH4 was added to mixture in small portions, effervescences were observed. Then reaction was left for stirring at room temperature overnight. After that, water was added to quench the reaction, and remaining methanol was evaporated under reduced pressure. As soon as methanol was evaporated the desired product precipitate as white solid. The product was used in the next step without further purification (2.8 g, yield 65%).

^{1}H NMR (400 MHz, $CDCl_3$) δ 8.15 (dt, J = 8.3, 0.8 Hz, 1H), 8.08 (s, 1H), 7.69 (t, J = 7.9 Hz, 1H), 6.98 (dq, J = 7.6, 0.8 Hz, 1H), 4.68 (d, J = 4.4 Hz, 2H), 3.54 (t, J = 5.3 Hz, 1H), 1.33 (s, 9H).

^{13}C NMR (101 MHz, $CDCl_3$) δ 177.26, 157.62, 151.04, 139.32, 116.31, 112.42, 64.11, 39.99, 27.62(3C).

MF: C11H16N2O2 (208.26 g/mol)

ESI-MS(M/Z):m/z = 209.19 [M+H]

N-(6-(bromomethyl)pyridin-2-yl)pivalamide (c):

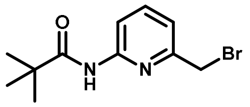

PBr3 (1.26 mL, 0.0156 mol, 1.2 eq.) was added dropwise to a solution of N-(6-(hydroxymethyl)pyridin-2-yl)pivalamide (2.8 g, 0.0131 mol) in 70 mL chloroform at 0oC. The solution was stirred at room temperature for 5 hr. After that, saturated Na2CO3 solution was added and solution was extracted with DCM. The organic layers were dried over anhydrous MgSO4 and solvent was evaporated in rotavapor to give light yellow oil. The product was purified using silica column with 2-5% MeOH in DCM. Light yellow oil was obtained as product. (2.8 g, ~ 85%)

^{1}H NMR (400 MHz, $CDCl_3$) δ 8.17 (dd, J = 8.4, 0.8 Hz, 1H), 7.99 (s, 1H), 7.72 – 7.64 (m, 1H), 7.13 (dd, J = 7.5, 0.9 Hz, 1H), 4.41 (s, 2H), 1.32 (s, 9H).

^{13}C NMR (101 MHz, $CDCl_3$) δ 177.26, 155.03, 151.48, 139.48, 119.25, 113.43, 39.96, 33.49, 27.61(3C)

ESI-MS(M/Z): 336.33 (100) [M+H+], 358.25 (45) [M+Na+]

N-(6-((bis(pyridin-2-ylmethyl)amino)methyl)pyridin-2-yl)pivalamide:

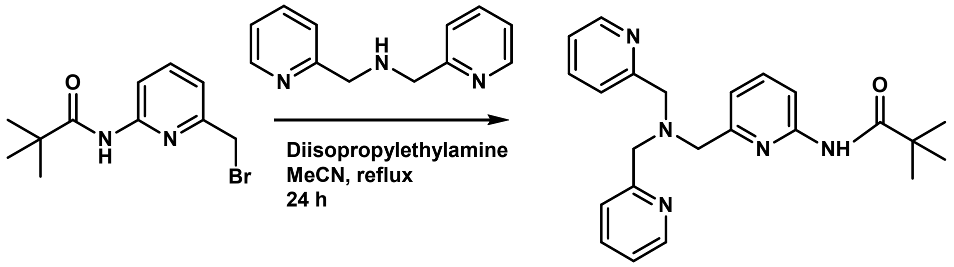

A reported procedure was followed for this synthesis. [2] To N-(6-(bromomethyl)pyridin-2-yl)pivalamide (1g, 3.7 mmol, 1 eq.) solution in 50 mL EtOAC, 2,2'-Dipicolylamine (0.734 g, 3.7 mmol, 1 eq.) and diisopropylethylamine (1.19g, 9.25 mmol, 2.5 eq.) were added and the solution was stirred for 24 h at room temperature. EtOAc was removed via reduced pressure after the reaction was complete, affording a brown oil. The residue was dissolved in an aqueous sodium carbonate solution and extracted into DCM. The organic layers were dried over sodium sulfate and the solvent removed by rotary evaporation. The resulting oil was purified using a silica column with EtOAC/Heptane solvent system. The brownish solid (0.86 g) was obtained in 60% yield.

^{1}H NMR (500 MHz, $CDCl_3$) δ 8.52 (dd, J = 4.9, 2.7 Hz, 1H), 8.07 (d, J = 8.4, 0.9 Hz, 1H), 7.94 (s, 1H), 7.67 – 7.61 (m, 3H), 7.55 (d, J = 7.9, 1.1 Hz, 2H), 7.29 (d, J = 7.5, 0.9 Hz, 1H), 7.13 (dd, J = 7.5, 4.8, 1.3 Hz, 2H), 3.87 (s, 4H), 3.76 (s, 2H), 1.31 (s, 9H).

^{13}C NMR (126 MHz, $CDCl_3$) δ 177.15, 159.50, 157.90, 150.99, 149.21, 138.84, 136.54, 122.97, 122.14, 118.69, 112.03, 60.41, 59.95, 27.62.

MF: C28H36N6O2 (488.64 g/mol)

ESI-MS(M/Z): 390.35 [M+H+], 412.24 [M+Na+]

6-((bis(pyridin-2-ylmethyl)amino)methyl)pyridin-2-amine (MAPA):

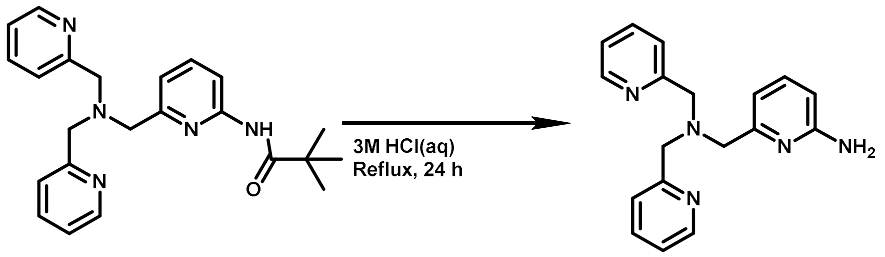

0.7 g of N-(6-((bis(pyridin-2-ylmethyl)amino)methyl)pyridin-2-yl)pivalamide was dissolved in 80 mL of 3M HCl (aq.) and refluxed for 24 h. After refluxing, 4M NaOH was added to neutralize the HCl. The solution was extracted with dichloromethane and organic layers were dried over anhydrous Na2SO4. The organic solvent was evaporated to get brown oil. Which was recrystallized using ethanol-water to give brownish solid (0.49 g). (~90% yield).

^{1}H NMR (500 MHz, $CDCl_3$) δ 8.50 (dd, J = 4.9, 1.3 Hz, 2H), 7.63 (td, J = 7.5, 1.8 Hz, 2H), 7.59 (d, J = 7.7 Hz, 2H), 7.38 (t, J = 7.7 Hz, 1H), 7.11 (t, J = 6.7, 4.8, 1.5 Hz, 2H), 6.91 (d, J = 7.4 Hz, 1H), 6.33 (d, J = 8.0 Hz, 1H), 4.47 (s, 3H), 3.87 (s, 4H), 3.69 (s, 2H).

^{13}C NMR (126 MHz, CDCl3) δ 159.74, 158.10, 157.92, 149.13, 138.26, 136.49, 122.96, 121.58, 112.81, 106.87, 60.28 (2 C), 60.21.

ESI-MS(M/Z): 306 (100) [M+H+], 328 [M+Na+]

N,N'-((((pyridin-2-ylmethyl)azanediyl)bis(methylene))bis(pyridine-6,2-diyl))bis(2,2-dimethylpropanamide):

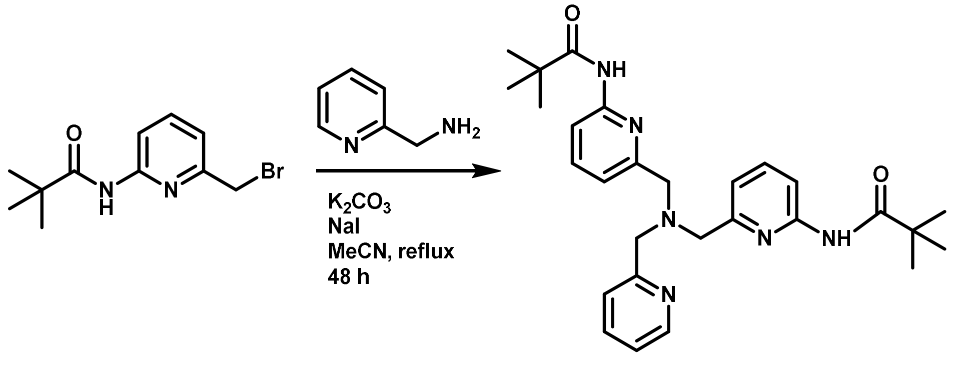

2g (7.4 mmol) of N-(6-(bromomethyl)pyridin-2-yl)pivalamide, 380 mL (3.7 mmol) of picolyl amine, 1.5 g (11 mmol) of potassium carbonate, and 1.1 g (7.4 mmol) of sodium iodide was dissolved in 70 mL acetonitrile. As described in the previously reported procedure. [3] The mixture was left on heating at 70oC for 48h, after that brown color mixture was cooled down to room temperature and the solvent was removed in rotavapor. The resulting residue was extracted with dichloromethane-water. The organic layers were collected and dried over anhydrous Na2SO4. The organic solvent was evaporated in rotavapor to yield brown solid. The crude solid was purified with silica column with 4-8% MeOH (ammonia saturated)/DCM solvent system. Final product was obtained as light brown solid. (2.5 g, 70%)

^{1}H NMR (500 MHz, $CDCl_3$) δ 8.53 (dd, J = 4.9, 0.9 Hz, 1H), 8.10 (d, J = 7.4 Hz, 2H), 7.95 (bs, 4H, NH-), 7.72 – 7.65 (m, 2H), 7.64 (d, J = 1.8 Hz, 1H), 7.55 (d, J = 9.0 Hz, 1H), 7.30 (d, J = 6.7 Hz, 2H), 7.18 – 7.12 (m, 2H), 3.88 (s, 2H), 3.76 (s, 4H), 1.32 (s, 18H).

^{13}C NMR (126 MHz, $CDCl_3$) δ 177.13, 159.50, 157.86, 151.02, 149.20, 138.87, 136.56, 122.86, 122.17, 118.63, 112.08, 60.39, 59.92, 27.63.

ESI-MS(M/Z): 511 (100) [M+Na+]

6-((((6-aminopyridin-2-yl)methyl)(pyridin-2-ylmethyl)amino)methyl)pyridin-2-amine (BAPA):

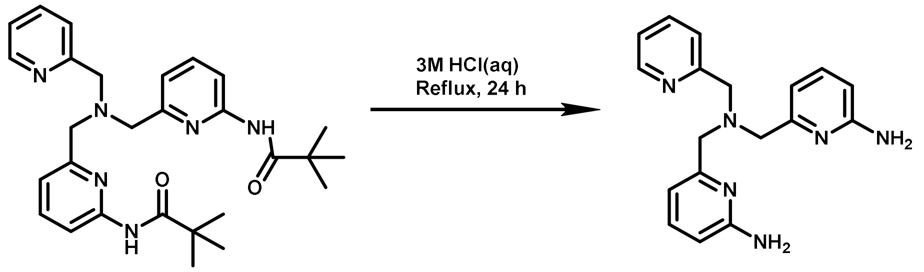

2 g of N,N'-((((pyridin-2-ylmethyl)azanediyl)bis(methylene))bis(pyridine-6,2-diyl))bis(2,2-dimethylpropanamide) was dissolved in 100 mL of 3M HCl (aq.) and refluxed overnight. After refluxing, 4M NaOH was added to neutralize the HCl until the brown precipitation appeared. The solution was extracted with dichloromethane and organic layers were dried over anhydrous Na2SO4. [3] The organic solvent was evaporated to get light brown solid. (1.2 g, 93 % yield)

^{1}H NMR (500 MHz, DMSO): δ 8.48 (dd, J = 4.9, 1.8, 0.9 Hz, 1H), 7.78 (t, J = 7.7, 1.8 Hz, 1H), 7.62 (dt, J = 7.8, 1.1 Hz, 1H), 7.35 (dd, J = 8.2, 7.3 Hz, 2H), 7.24 (t, J = 7.5, 4.8, 1.2 Hz, 1H), 6.75 (d, J = 7.3, 0.9 Hz, 2H), 6.29 (d, J = 8.2, 0.9 Hz, 2H), 5.81 (s, 4H), 3.73 (s, 2H), 3.50 (s, 4H).

^{13}C NMR (126 MHz, DMSO) δ 159.39, 159.19, 157.30, 148.77, 137.48, 136.57, 122.25, 122.04, 109.74, 106.10, 59.50, 59.44.

ESI-MS(M/Z): 321 (100) [M+H+], 344 [M+Na+]

N,N',N"-((nitrilotris(methylene))tris(pyridine-6,2-diyl))tris(2,2-dimethylpropanamide):

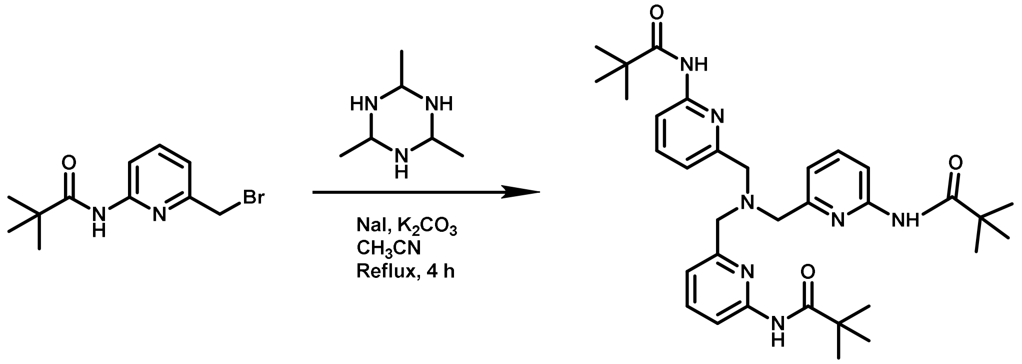

The procedure was adapted from literature for the synthesis of symmetrical tripodal ligands. [4] 2 g (7.3 mmol) of N-(6-(bromomethyl)pyridin-2-yl)pivalamide, (0.45 g, 2.4 mmol) of acetaldehyde ammonia trimer trihydrate, (1.1g, 7.5 mmol) of sodium iodide, and (1.1 g, 7.9 mmol) of potassium carbonate was mixed with 100 mL acetonitrile and refluxed for 4 hrs. The solvent was evaporated in rotavapor and residue was extracted with chloroform-water. Organic layers were washed with brine and dried over anhydrous sodium sulfate. The solvent was evaporated under reduced pressure to give brown solid. (85%). The crude product was eluted on the silica column with 5-10% ammonia saturated MeOH-DCM to give light brown solid as final product. (3.5 g)

^{1}H NMR (400 MHz, $CDCl_3$) δ 8.10 (dd, J = 8.3, 1.0 Hz, 3H), 7.96 (s, 3H), 7.66 (t, J = 7.9 Hz, 3H), 7.30 – 7.22 (m, 3H), 3.74 (s, 6H), 1.32 (s, 27H).

^{13}C NMR (101 MHz, $CDCl_3$) δ 177.17(3C), 157.73(3C), 151.09(3C), 138.93(3C), 118.65(3C), 112.19(3C), 59.90(3C), 39.92(3C), 27.64(9C).

MF: C33H45N7O3 (587.77 g/mol).

ESI-MS(M/Z): 610.58 (100) [M+Na+]

N,N',N"-((nitrilotris(methylene))tris(pyridine-6,2-diyl))tris(2,2-dimethylpropanamide)(TAPA):

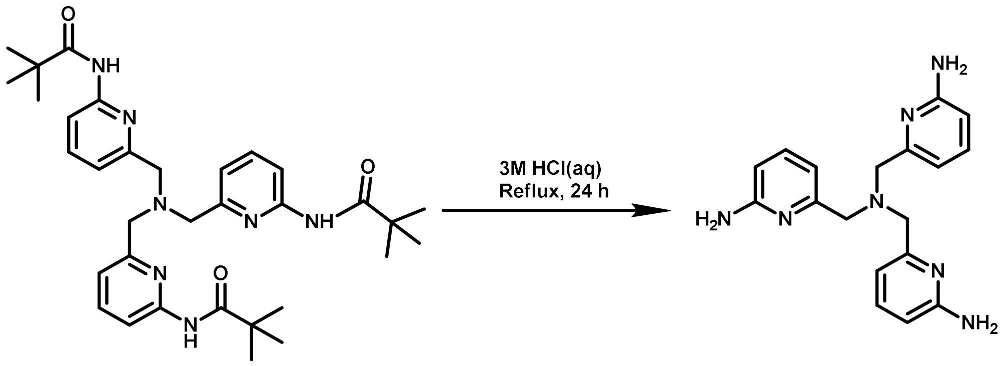

N,N',N"-((nitrilotris(methylene))tris(pyridine-6,2-diyl))tris(2,2-dimethylpropanamide) (2.1 g, 3.4 mmol) was dissolved in 100 mL of 3M HCl (aq.) and refluxed overnight. After refluxing, the solution was washed with DCM. To aqueous layer, 4M NaOH was added to neutralize the HCl until the brown precipitation appeared. The precipitate was filtered and washed with water then with diethyl ether. The light brown solid was obtained as TAPA. (0.652 g)

^{1}H NMR (400 MHz, 298K, DMSO-d6): δ 7.34 (dd, J = 8.2, 7.3 Hz, 3H), 6.74 (d, J = 7.3 Hz, 3H), 6.28 (d, J = 8.1 Hz, 3H), 5.81 (bs, 6H, N-H protons), 3.46 (s, 6H).

^{13}C NMR (101 MHz, 298K, DMSO-d6): δ 159.62, 158.10, 137.94, 109.99, 106.47, 60.11.

ESI-MS(M/Z): 336.33 (100) [M+H+], 358.25 (45) [M+Na+]

General procedure for the generation of $[(L)Co^{III}(O_2)]^+$

A 25 mM stock of $[(L)Co^{II}(NO_3)_2]$ was prepared by dissolving $Co(NO_3)_2{\cdot}6H_2O$ (7.2 mg, 25 µmol) and ligands L (25 µmol, 1 equiv.) (L=TPA, 7.3 mg; L=MAPA, 7.6 mg; L=BAPA, 8.0 mg; L=TAPA, 8.3 mg) in acetonitrile (1 mL). From this stock, a 25 µM solution was prepared. This solution was mixed in flow with a solution containing H_2O_2 (3.5% *v/v* in acetonitrile) prior to electrospray ionization (Figure S1)

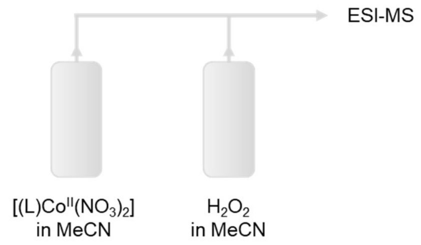

Figure S1. A schematic of the in-flow mixing setup used for the generation of $[(L)Co^{III}(O_2)]^+$

General procedure for the generation of D_n-$[(L)Co^{III}(O_2)]^+$

A 25 mM stock of $[(L)Co^{II}(NO_3)_2]$ was prepared by dissolving $Co(NO_3)_2{\cdot}6H_2O$ (7.3 mg, 25 µmol) and ligands L (25 µmol, 1 equiv.) (L=TPA, 7.2 mg; L=MAPA, 7.6 mg; L=BAPA, 7.9 mg; L=TAPA, 8.3 mg) in a mixture of acetonitrile:deuterium oxide (1 mL, 1:1 *v*/*v*). From this stock, a 25 µM solution was prepared. This solution was mixed in flow with a solution containing H_2O_2 (3.5% *v/v* in acetonitrile:deuterium oxide, 1:1, *v*/*v*) prior to electrospray ionization (Figure S2)

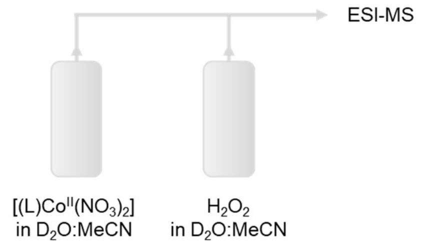

Figure S2. A schematic of the in-flow mixing setup used for the generation of D_n-$[(L)Co^{III}(O_2)]^+$

Mass spectrometric studies

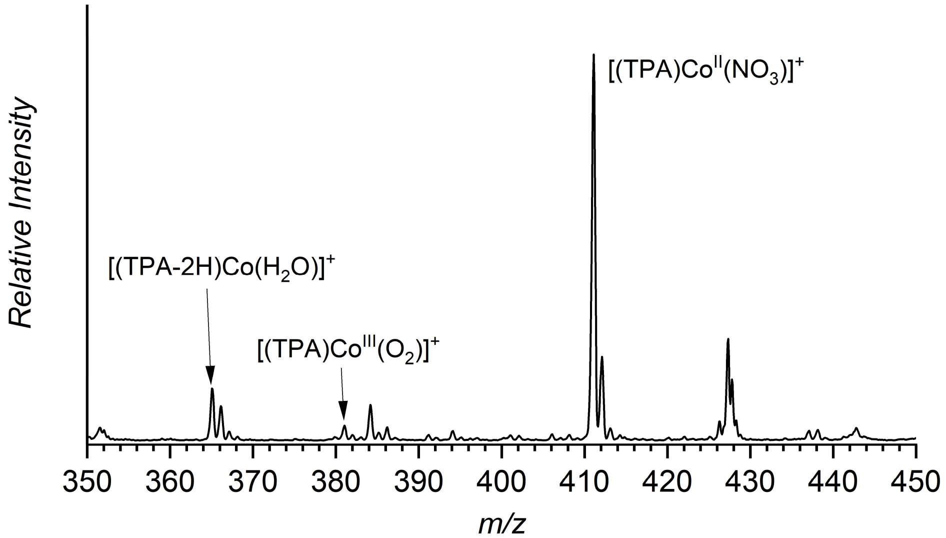

Figure S3. Source spectrum for the studies on $[(TPA)Co^{III}(O_2)]^+$ as generated *via* the general procedure for $[(L)Co^{III}(O_2)]^+$.

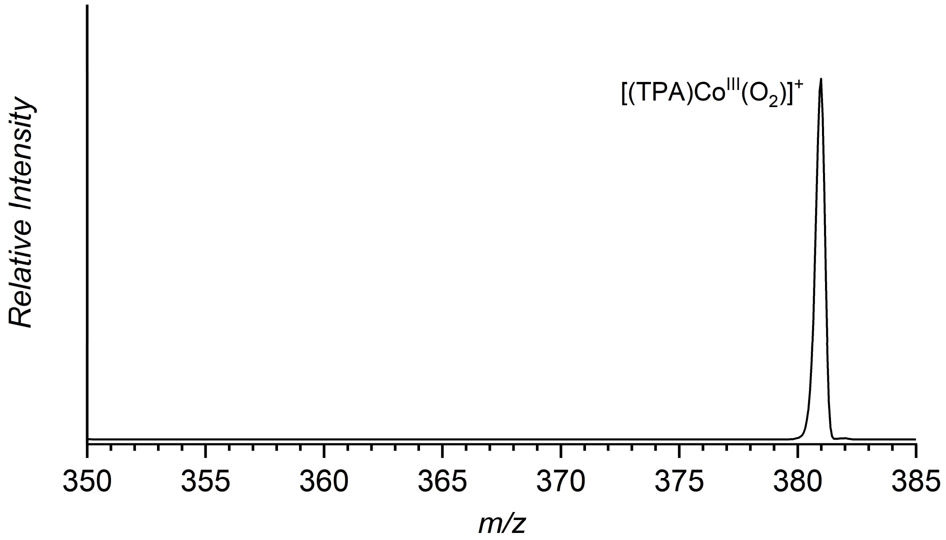

Figure S4. The selection of $[(TPA)Co^{III}(O_2)]^+$ in the ion trap with a selection width of 1.3 *m/z*.

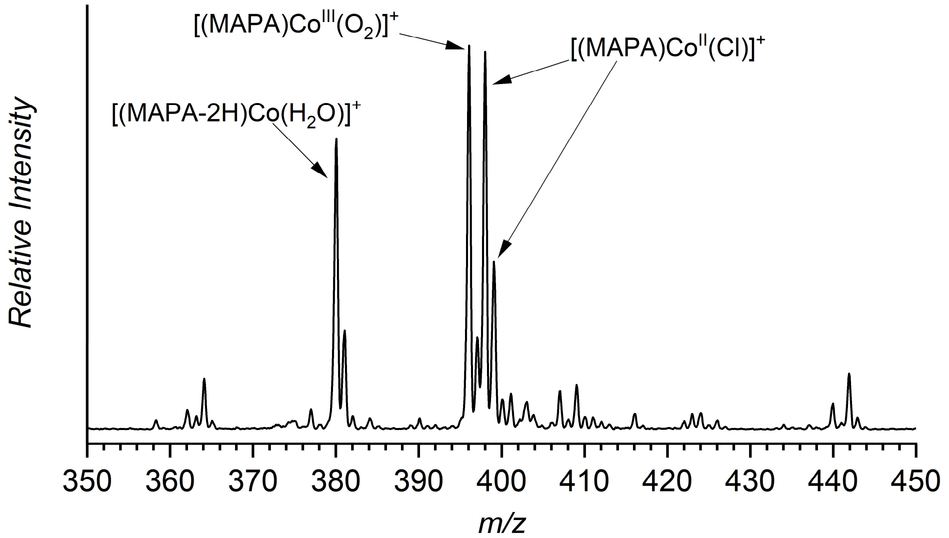

Figure S5. Source spectrum for the studies on [(MAPA)Co^{III}(O$_2$)]$^+$ as generated *via* the general procedure for [(L)Co^{III}(O$_2$)]$^+$.

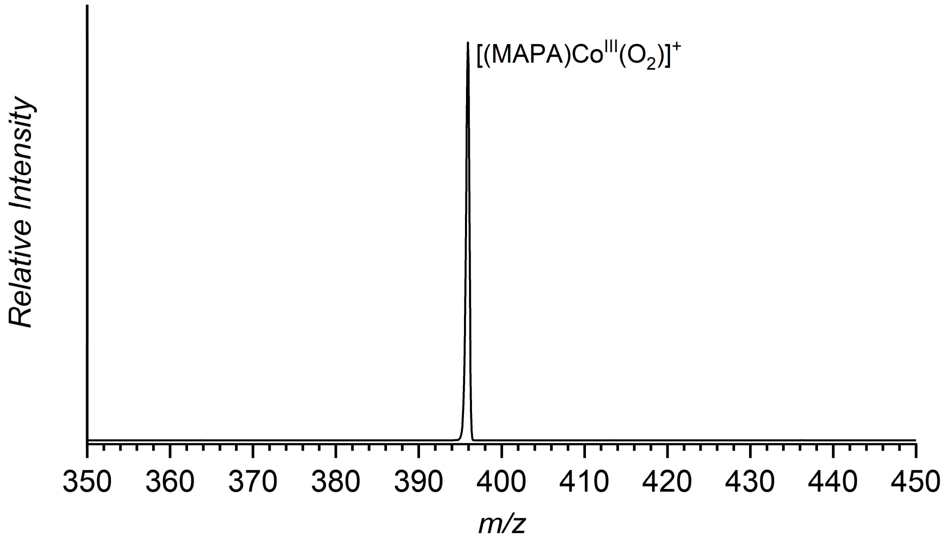

Figure S6. The selection of [(MAPA)Co^{III}(O$_2$)]$^+$ in the ion trap with a selection width of 1.3 *m/z*.

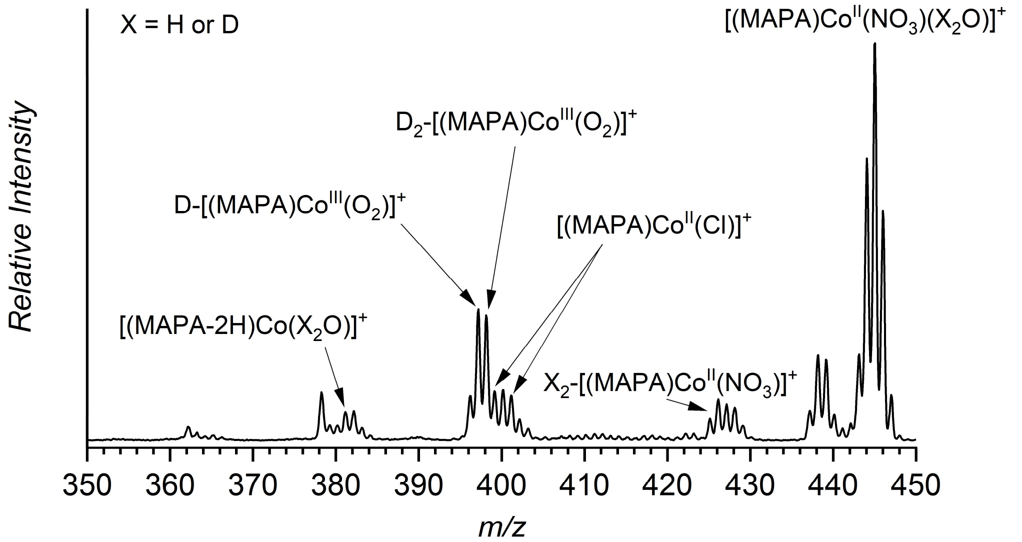

Figure S7. Source spectrum for the studies on D_2-[(MAPA)$Co^{III}(O_2)$]$^+$ as generated *via* the general procedure for D_n-[(L)$Co^{III}(O_2)$]$^+$.

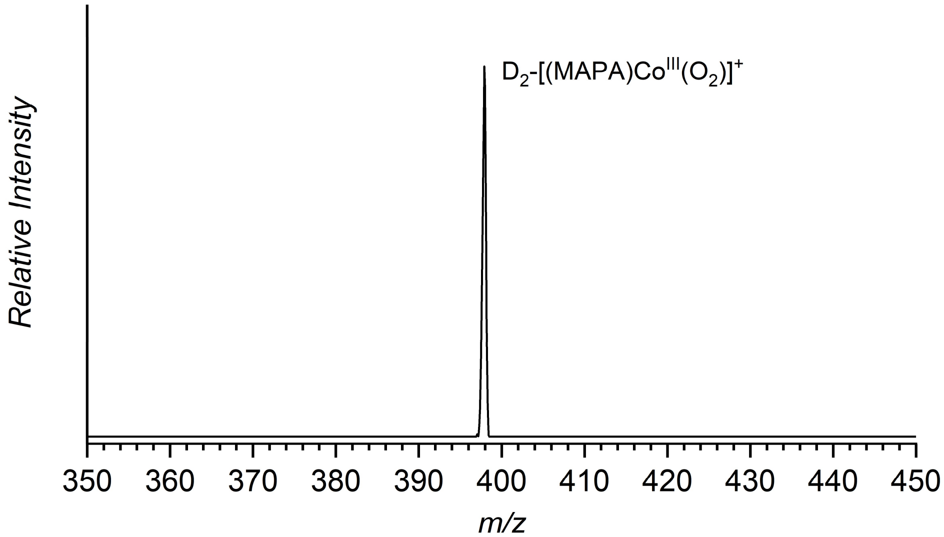

Figure S8. The selection of D_2-[(MAPA)$Co^{III}(O_2)$]$^+$ in the ion trap with a selection width of 0.8 *m/z*.

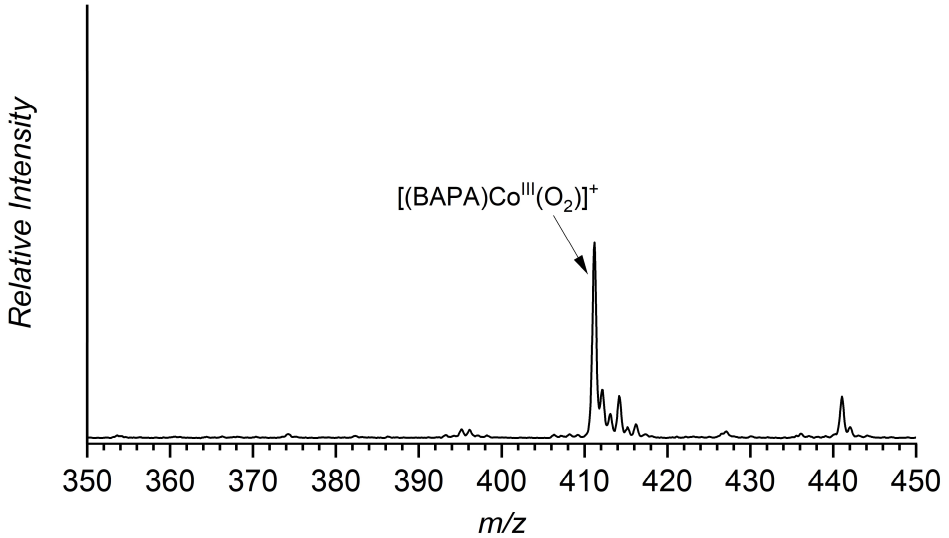

Figure S9. Source spectrum for the studies on [(BAPA)Co^{III}(O$_2$)]$^+$ as generated *via* the general procedure for [(L)Co^{III}(O$_2$)]$^+$.

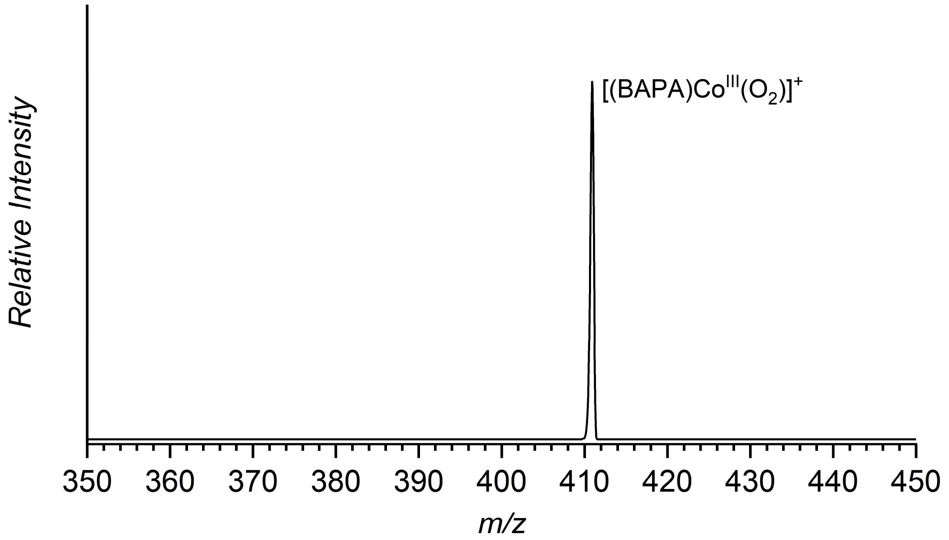

Figure S10. The selection of [(BAPA)Co^{III}(O$_2$)]$^+$ in the ion trap with a selection width of 1.3 *m/z*.

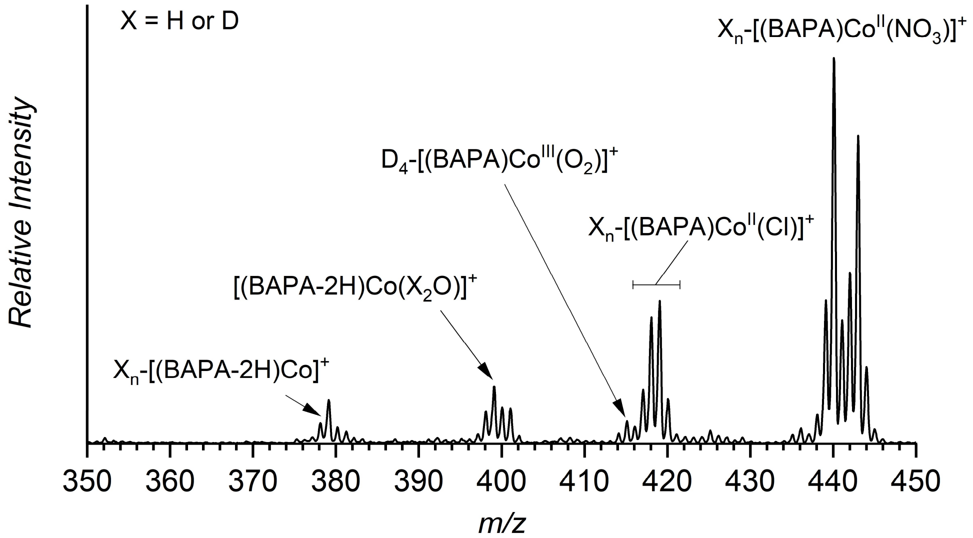

Figure S11. Source spectrum for the studies on D_4-[(BAPA)Co^{III}(O_2)]$^+$ as generated *via* the general procedure for D_n-[(L)Co^{III}(O_2)]$^+$.

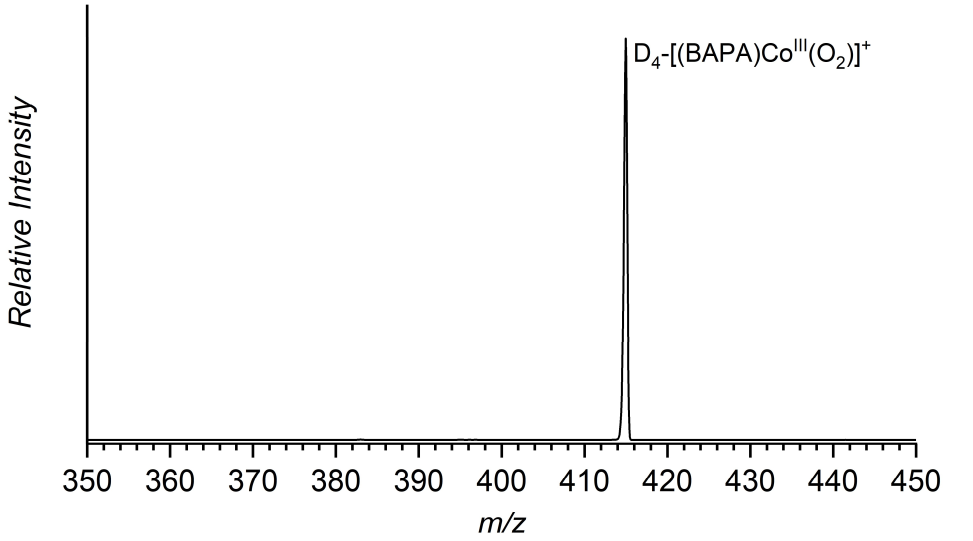

Figure S12. The selection of D_4-[(BAPA)Co^{III}(O_2)]$^+$ in the ion trap with a selection width of 0.8 *m/z*.

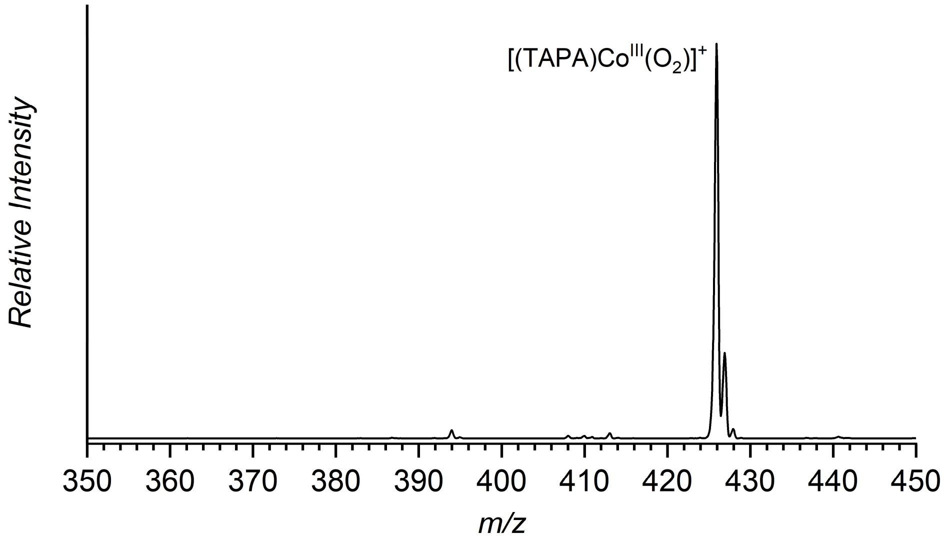

Figure S13. Source spectrum for the studies on [(TAPA)Co^{III}(O_2)]$^+$ as generated *via* the general procedure for [(L)Co^{III}(O_2)]$^+$.

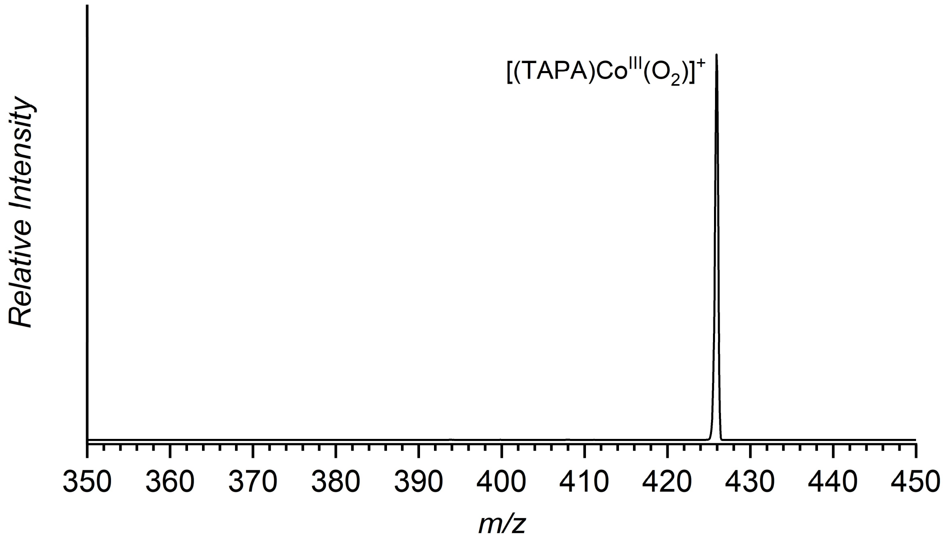

Figure S14. The selection of [(TAPA)Co^{III}(O_2)]$^+$ in the ion trap with a selection width of 1.3 *m/z*.

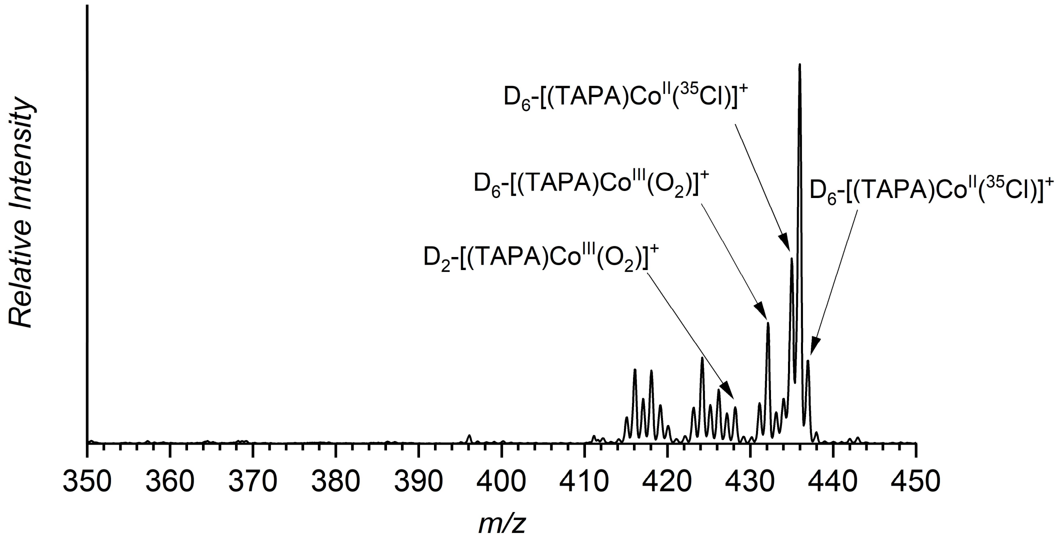

Figure S15. Source spectrum for the studies on D_6-[(TAPA)Co^{III}(O_2)]$^+$ as generated *via* the general procedure for D_n-[(L)Co^{III}(O_2)]$^+$.

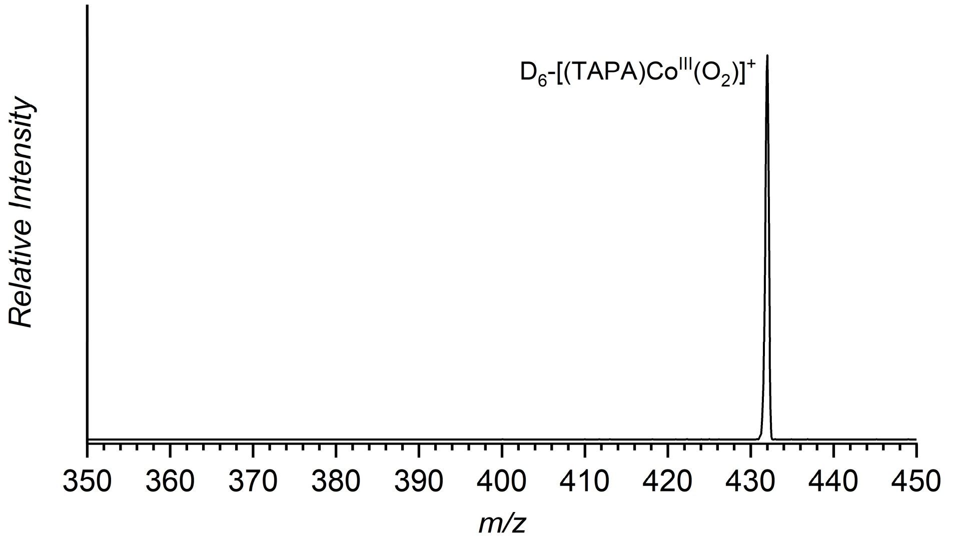

Figure S16. The selection of D_6-[(TAPA)Co^{III}(O_2)]$^+$ in the ion trap with a selection width of 0.8 *m/z*.

Energy resolved collision induced dissociation experiments

Table S1: The experimentally determined BDE's of thermometer ions converted to kJ mol^{-1} from eV, as determined by Armentrout.[5]

Entry	Structure	Experimental BDE (kJ mol^{-1})
p-H	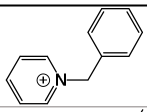	249±14.5
p-Me	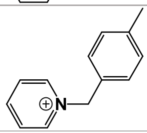	218±12.5
p-NO_2	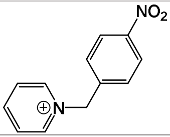	293±11.6
p-OMe	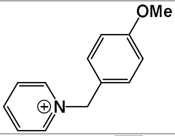	186±7.72
BH_HH	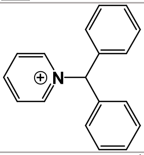	173±10.6
BH-MeMe	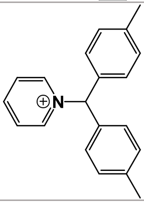	149±12.6
BH-HOMe	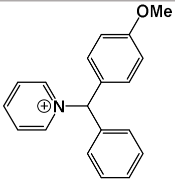	132±13.5

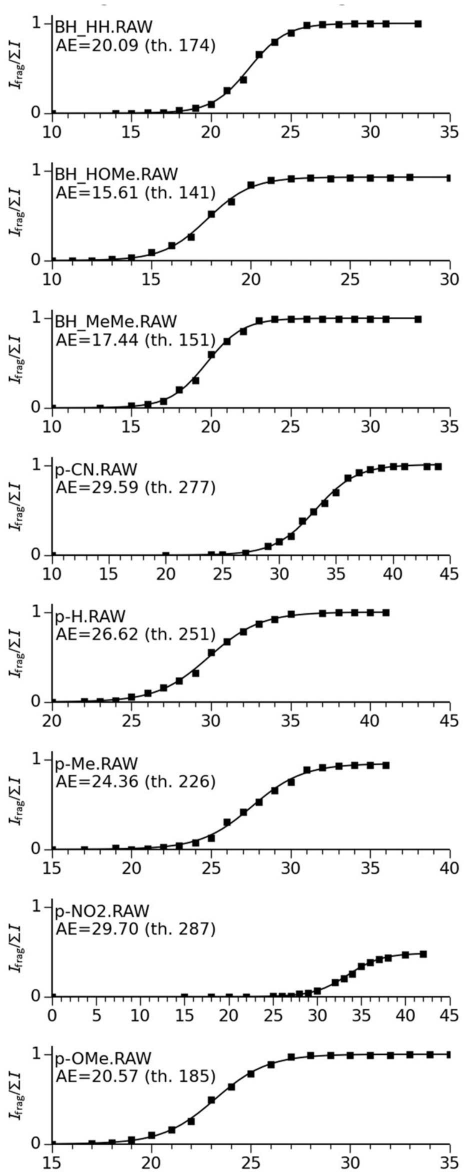

Figure S17. The determination of the activation energy for the thermometer ions in the ion trap instrument.

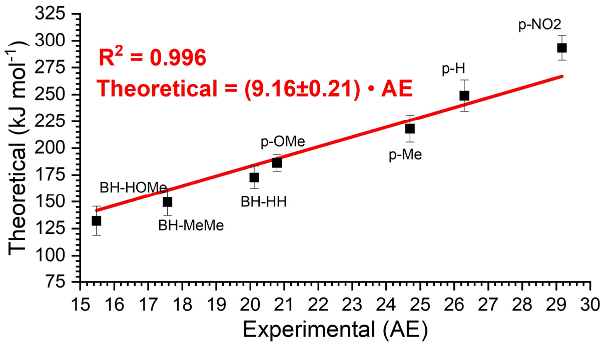

Figure S18: The calibration line for the ion trap using the benzylpyridinium and benzhydrylpyridinium ions. The appearance energy (AE) is plotted against the theoretical bond dissociation energy (kJ mol^{-1}).

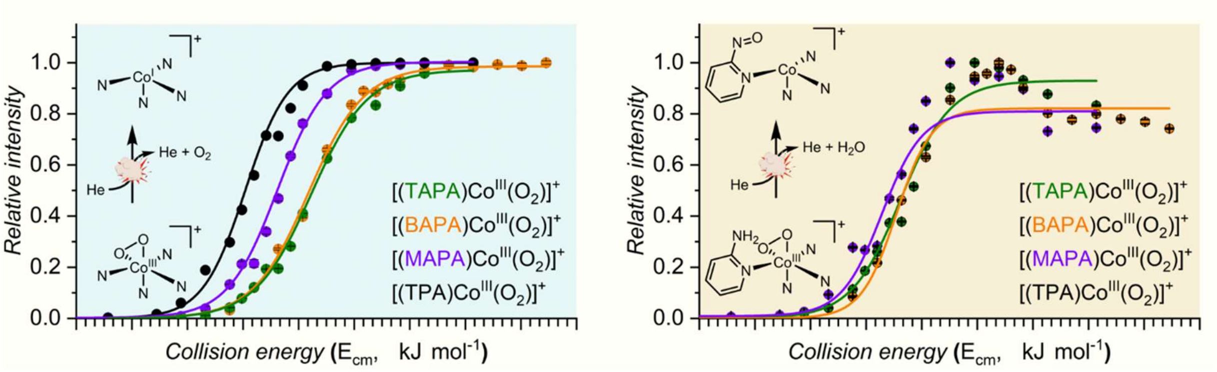

Figure S19. A scaled version of the energy resolved CID experiments providing a clearer depiction of the discrete energies for O_2 and H_2O dissociation.

Helium tagged photodissociation experiments and spectra

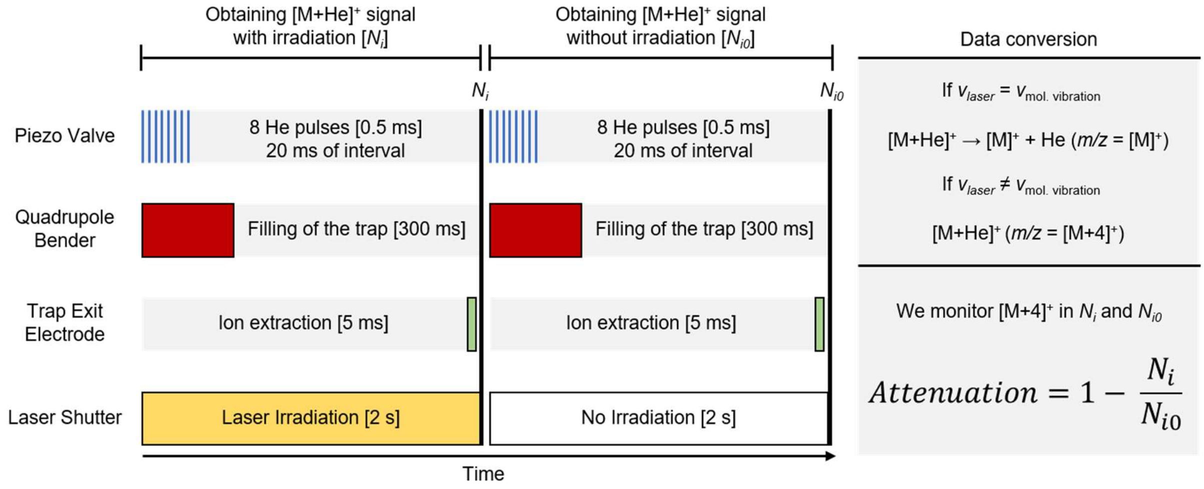

Figure S20: The experimental setup and pulse sequence for the helium tagged photodissociation experiments. The piezo valve controls the helium pulses (and therefore the helium tagging). The quadrupole bender controls the filling of the trap. The trap exit electrode controls the ion extraction towards the detector. The total experiment time is 4 seconds.

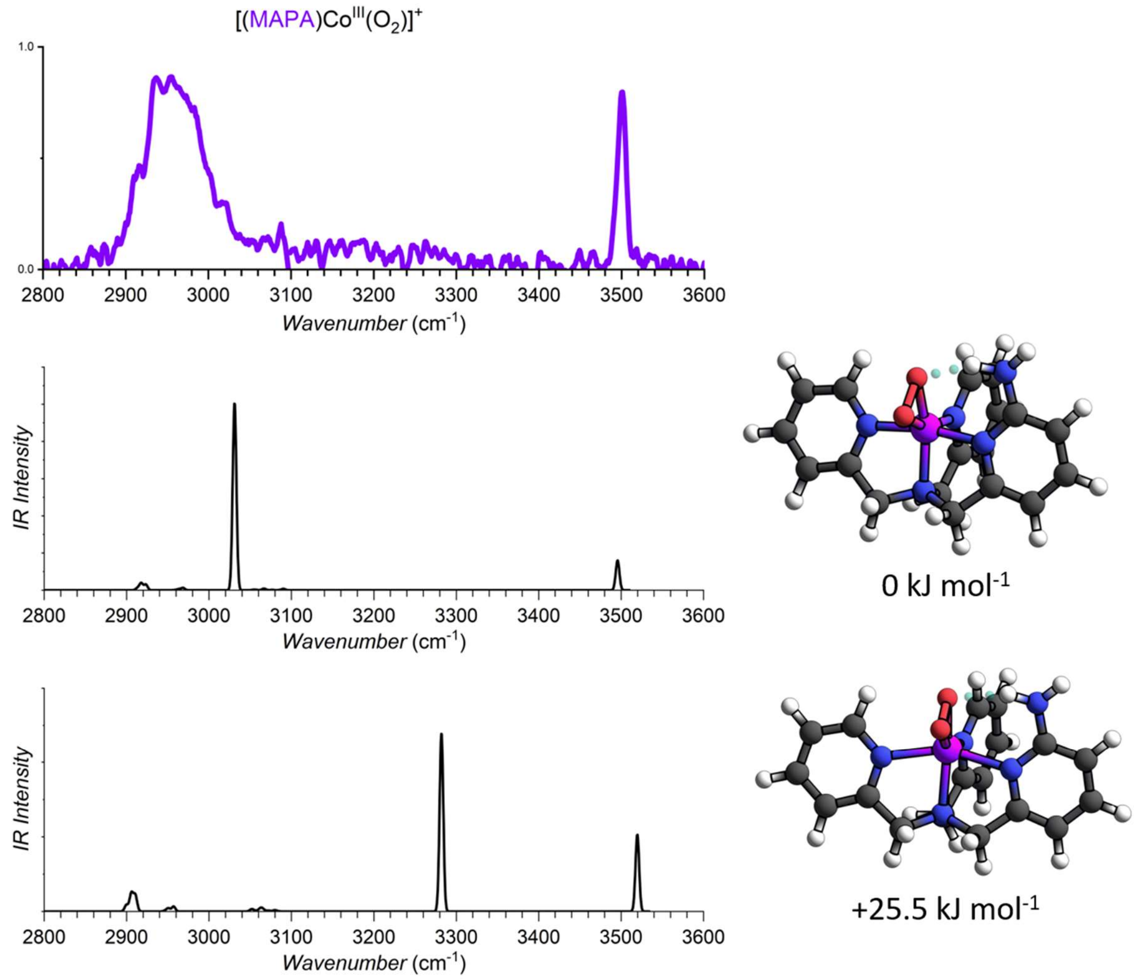

Figure S21. Helium tagging infrared photodissociation spectra of $[(MAPA)Co^{III}(O_2)]^+$ (*m/z* = 396), generated by electrospray ionization from acetonitrile solutions of the cobalt(II)nitrate complexes and H_2O_2 (top panel) and theoretical spectra or lowest in energy lying isomers of $[(MAPA)Co^{III}(O_2)]^+$ (B3LYP-D3/6-311G(2d,p), scaling factor: 0.96). Color code: blue, N; grey, C; pink, Co; red, O; white, H.

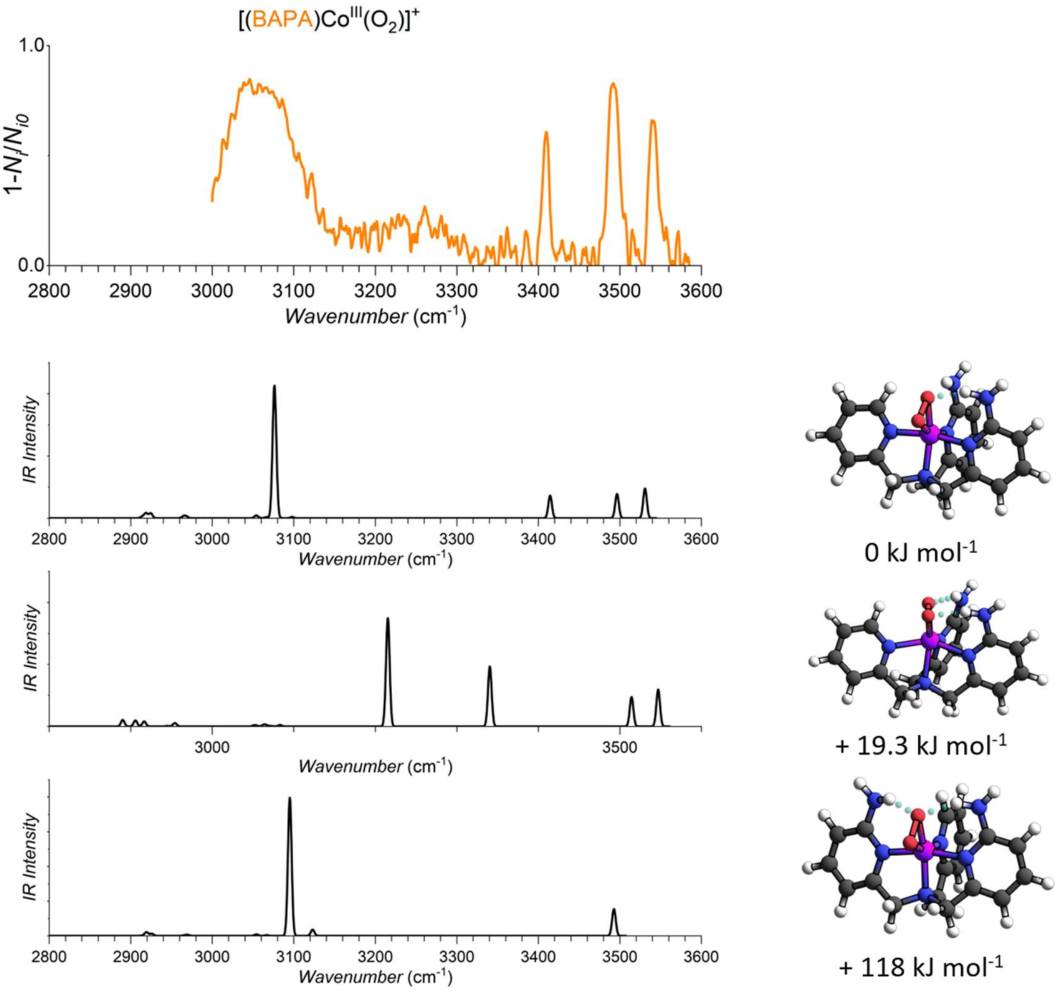

Figure S22. Helium tagging infrared photodissociation spectra of [(BAPA)Co^{III}(O$_2$)]$^+$ (*m/z* = 411), generated by electrospray ionization from acetonitrile solutions of the cobalt(II)nitrate complexes and H_2O_2 (top panel) and theoretical spectra or lowest in energy lying isomers of [(BAPA)Co^{III}(O$_2$)]$^+$ (B3LYP-D3/6-311G(2d,p), scaling factor: 0.96). Color code: blue, N; grey, C; pink, Co; red, O; white, H.

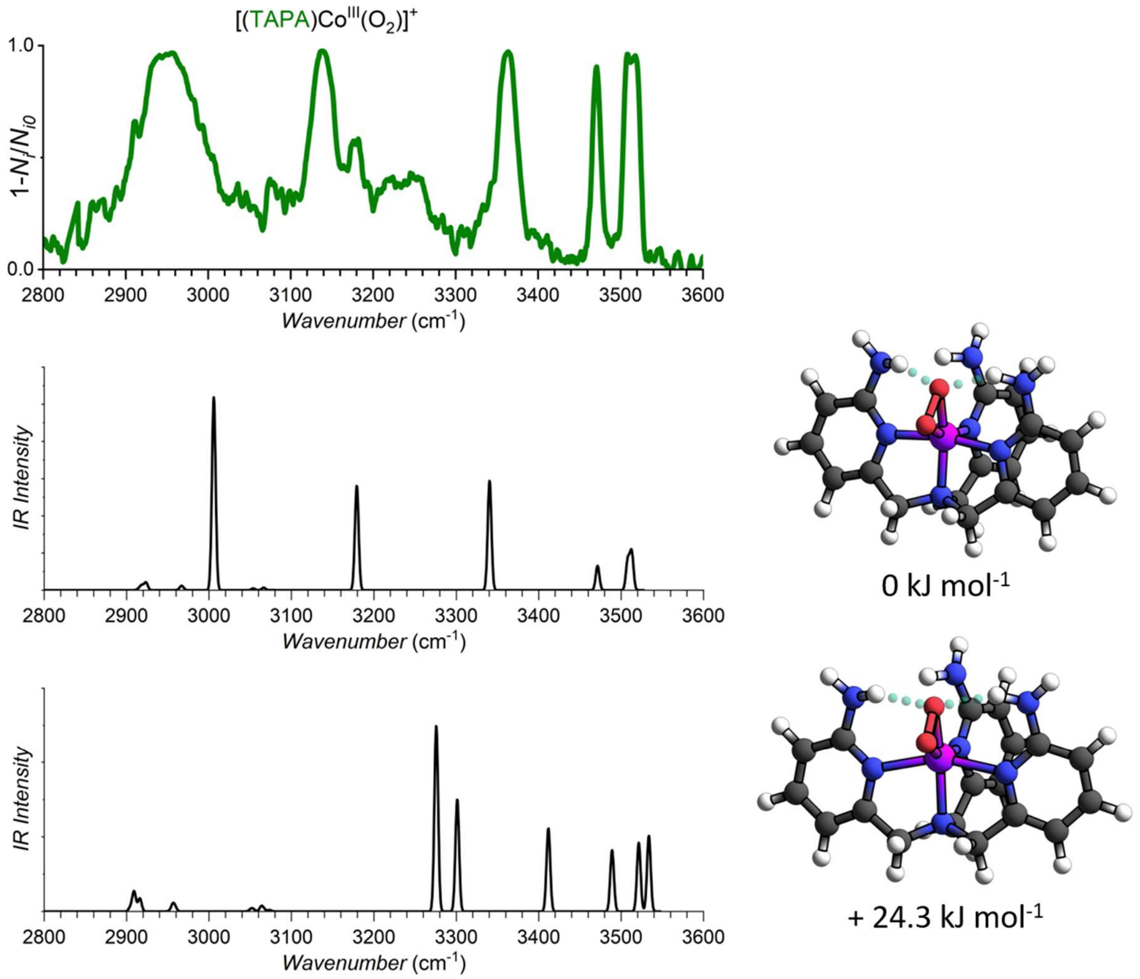

Figure S23. Helium tagging infrared photodissociation spectra of [(TAPA)Co^{III}(O$_2$)]$^+$ (*m/z* = 426), generated by electrospray ionization from acetonitrile solutions of the cobalt(II)nitrate complexes and H_2O_2 (top panel) and theoretical spectra or lowest in energy lying isomers of [(TAPA)Co^{III}(O$_2$)]$^+$ (B3LYP-D3/6-311G(2d,p), scaling factor: 0.96). Color code: blue, N; grey, C; pink, Co; red, O; white, H.

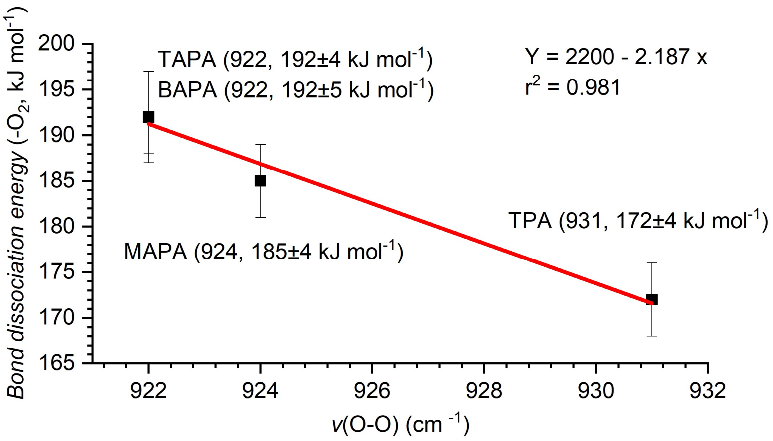

Figure S24. The correlation between the bond dissociation energy for O_2 elimination and the O-O stretching frequency.

Density functional theory calculations

All geometries were optimized and analyzed using the B3LYP-D3/6-311G(2d,p) level of theory.

Table S2: The relevant internuclear distances and angles for the ionic complexes $[(L)Co^{III}(O_2)]^+$, as determined by density functional theory calculations

	TPA	MAPA	BAPA	TAPA
**Co-O**	1.816	1.833	1.844	1.852
**Co-O'**	1.834	1.833	1.831	1.831
**O-O'**	1.417	1.420	1.422	1.420
α(**O-Co-O'**)	45.7	45.6	45.5	45.4
**(1)NH$_2$-O**	-	2.715	2.723	2.749
**(1)NH$_2$-O'**	-	3.418	3.385	3.520
**(2)NH$_2$-O**	-	-	3.033	3.169
**(2)NH$_2$-O'**	-	-	4.270	4.376
**(3)NH$_2$-O**	-	-	-	2.678
**(3)NH$_2$-O'**	-	-	-	3.439
**(1)Co-PyrN**	1.927	1.969	1.980	1.986
**(2)Co-PyrN**	1.965	1.964	1.988	2.008
**(3)Co-PyrN**	1.927	1.932	1.932	1.975
**(4)Co-PyrN**	2.025	2.009	1.996	1.990

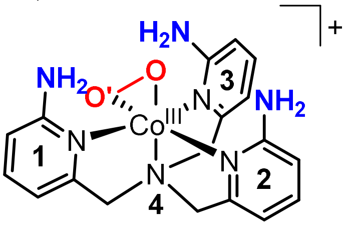

Inner sphere versus outer sphere mechanism

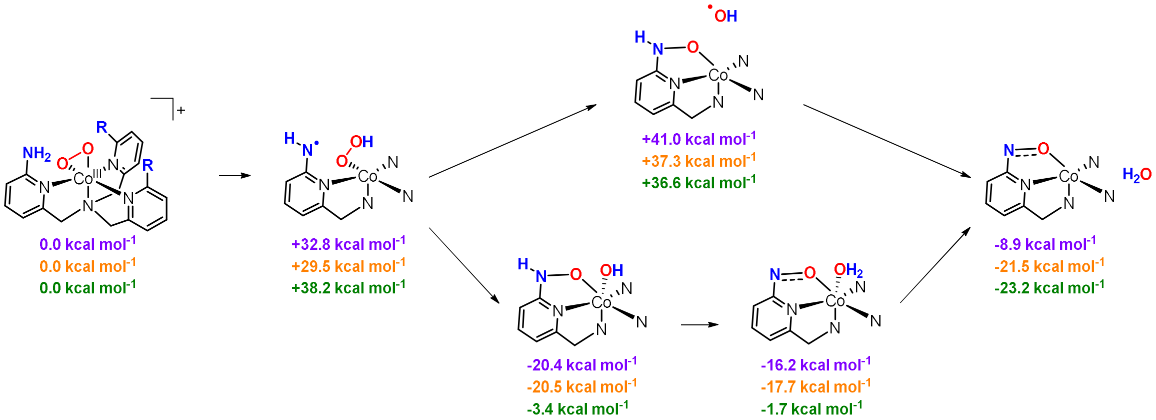

Scheme S2: The inner sphere versus outer sphere hydrogen atom transfer mechanism

NMR Spectra

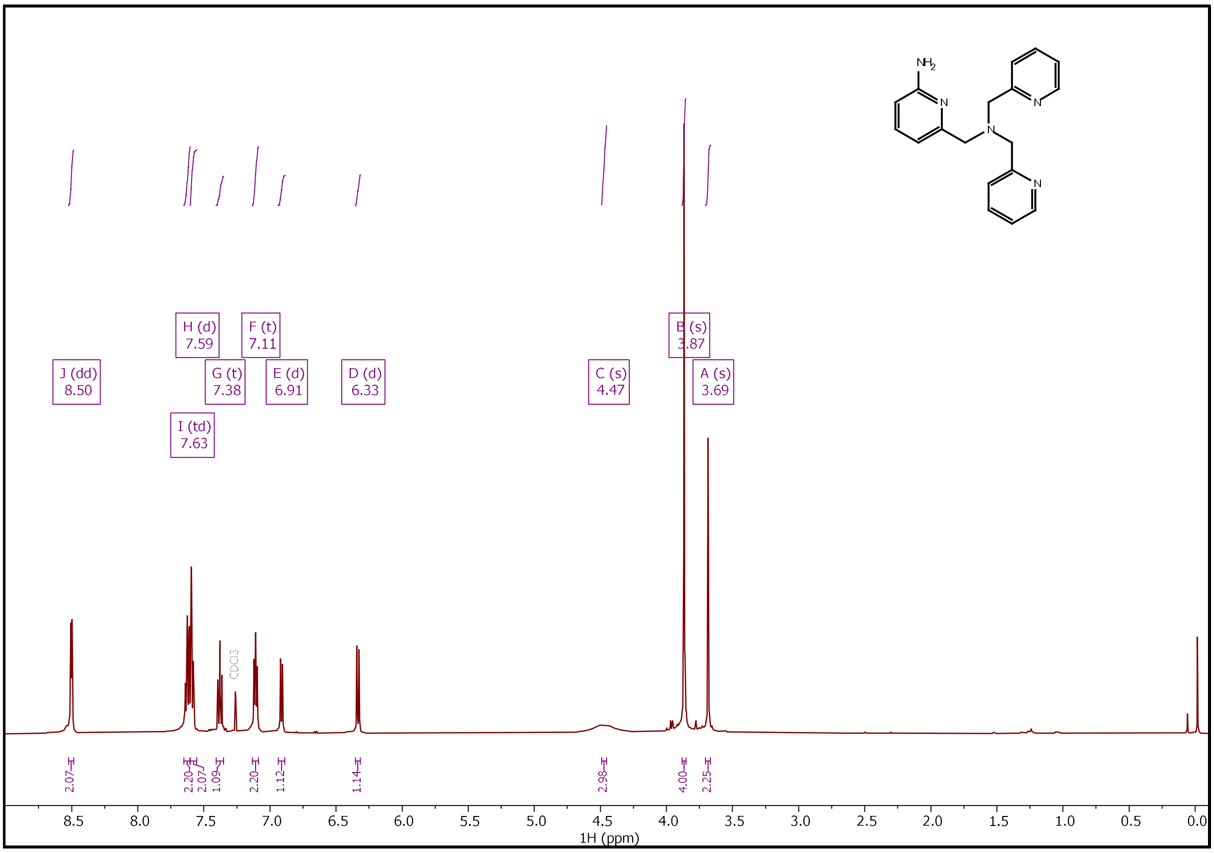

Figure S25. ^1H NMR of MAPA ($CDCl_3$)

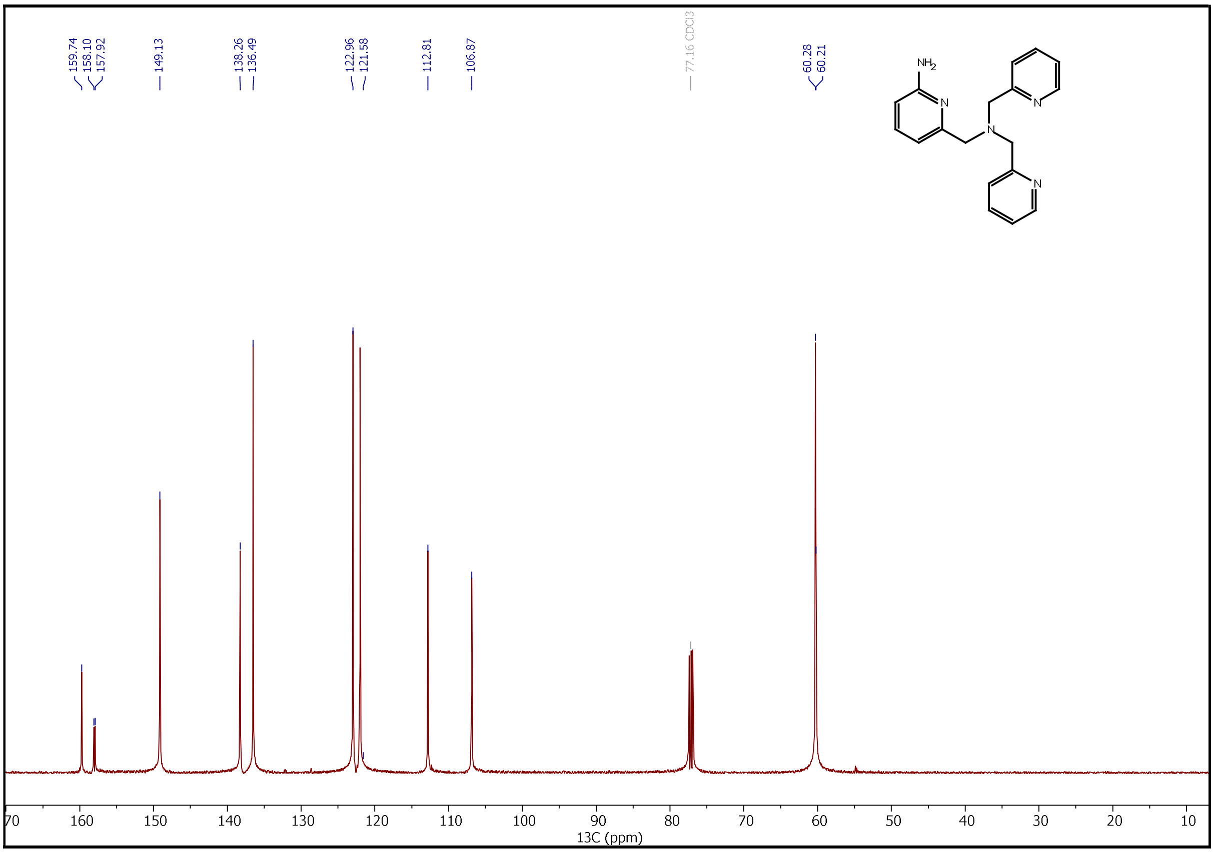

Figure S26. ^{13}C NMR of MAPA ($CDCl_3$)

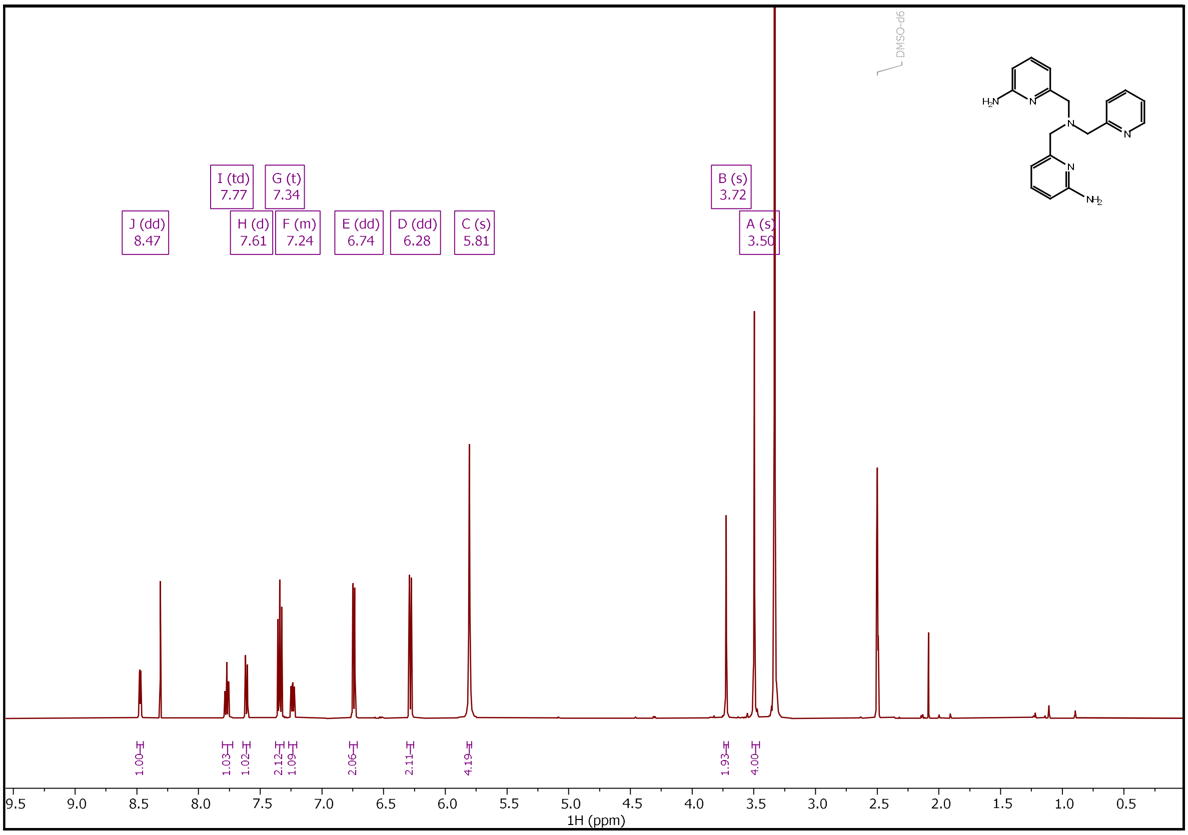

Figure S27. ^{1}H NMR of BAPA (DMSO-*d6*)

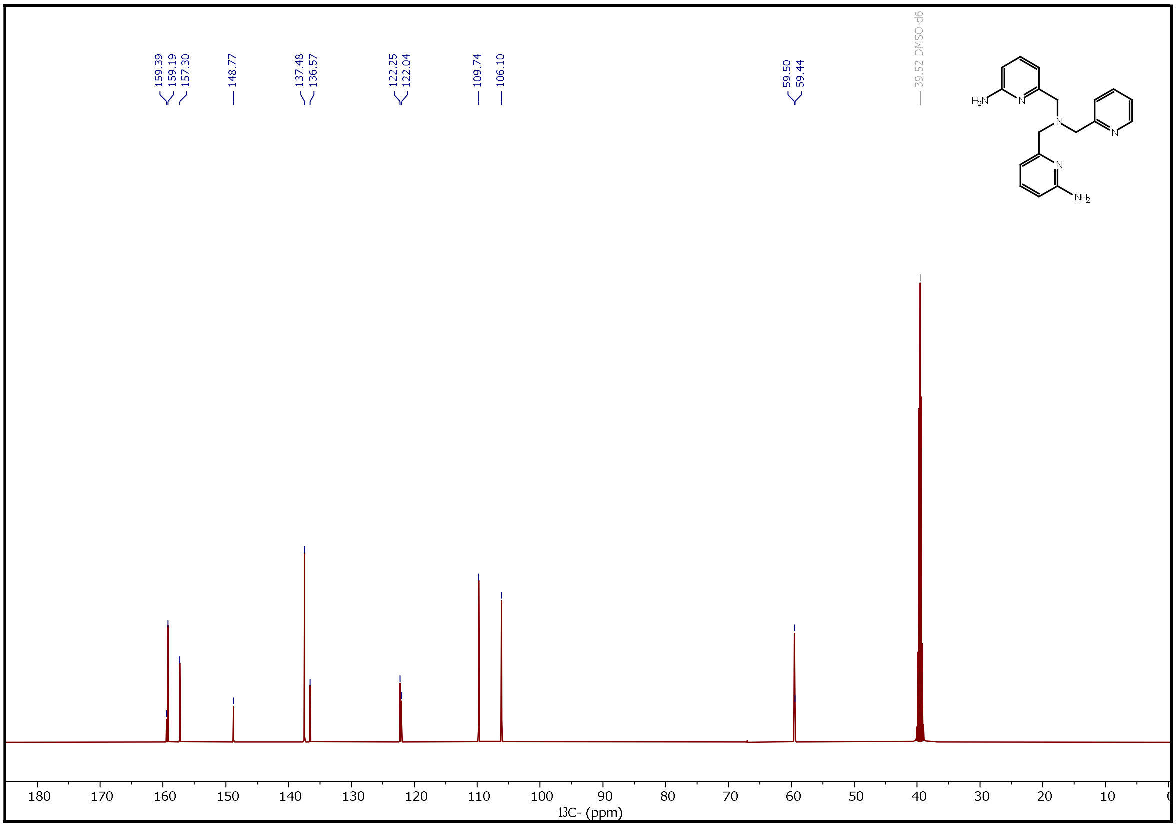

Figure S28. ^{13}C NMR of BAPA (DMSO-*d6*)

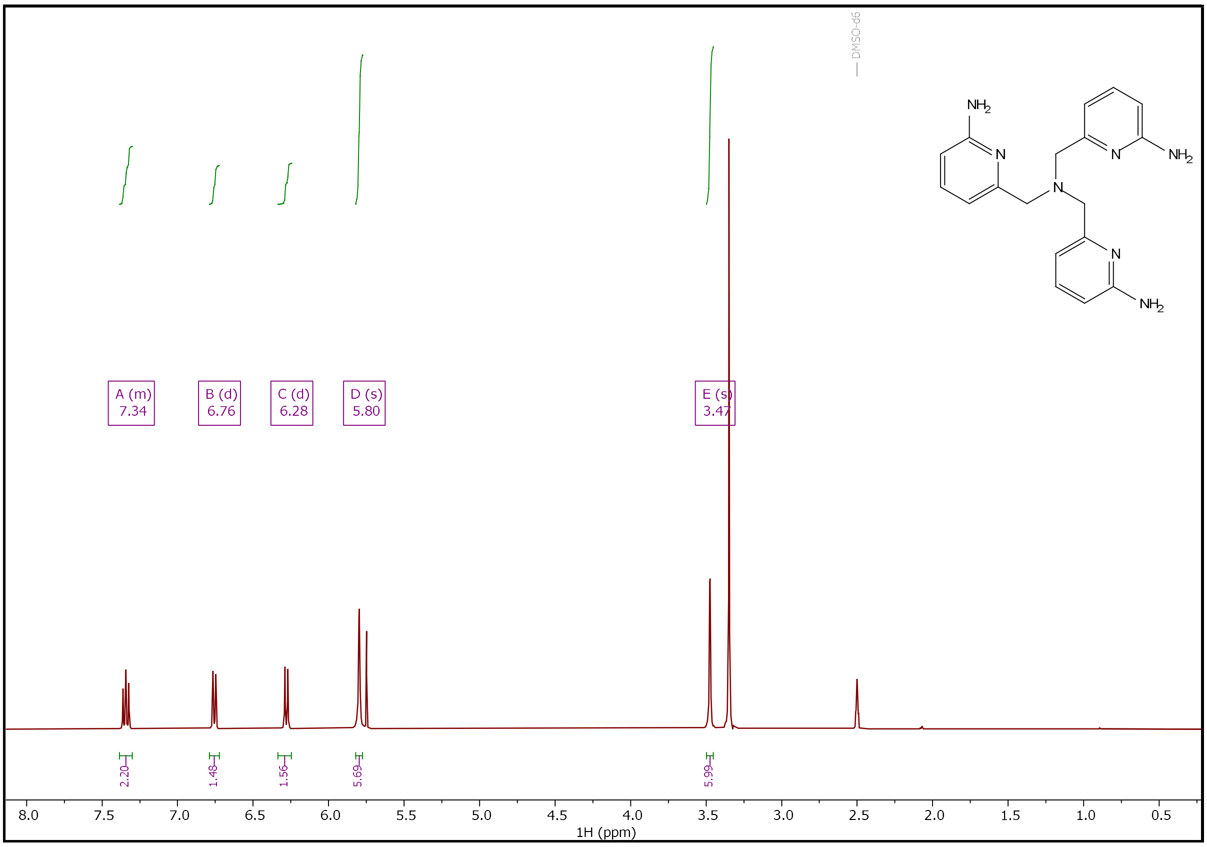

Figure S29. ^{1}H NMR of TAPA (DMSO-*d6*)

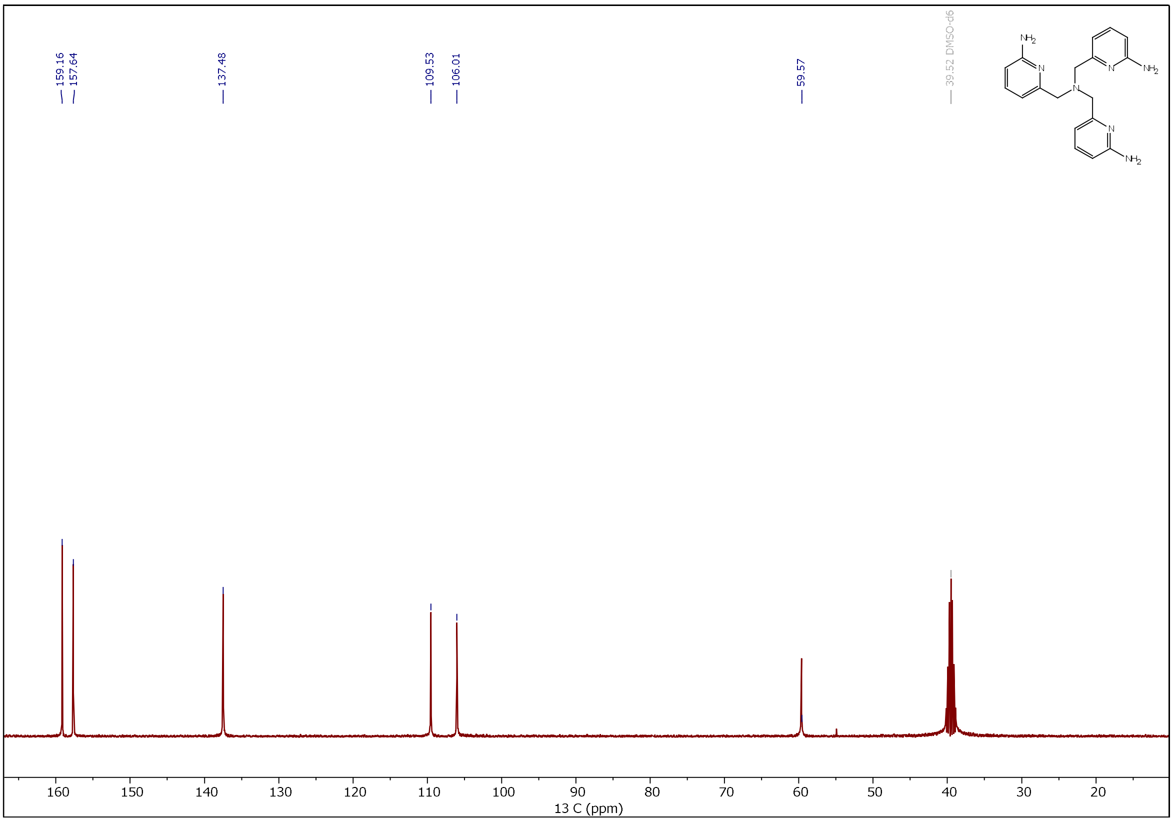

Figure S30. ^{13}C NMR of TAPA (DMSO-*d6*)

XYZ of calculated structures

[(TPA)Co^{III}(O$_2$)]$^+$ (singlet)

Co	0.065107000	-0.510928000	-0.543128000
O	0.176927000	-2.092406000	-1.464180000
N	1.984682000	-0.470915000	-0.377918000
N	-1.843219000	-0.722116000	-0.377573000
N	-0.108973000	1.426167000	-0.261325000
N	0.084966000	-0.620172000	1.478402000
C	1.354937000	-1.340229000	1.764574000
C	2.433873000	-0.845351000	0.833190000
C	3.786088000	-0.858146000	1.126198000
C	4.688781000	-0.505327000	0.126862000
C	4.211779000	-0.158858000	-1.130011000
C	2.841831000	-0.149974000	-1.348104000
C	-1.137772000	-1.396126000	1.813448000
C	-2.254678000	-1.054469000	0.858068000
C	-3.599992000	-1.180691000	1.158013000
C	-4.531288000	-0.988878000	0.141721000
C	-4.088589000	-0.687701000	-1.139470000
C	-2.726217000	-0.557291000	-1.364188000
C	0.078605000	0.757995000	2.056063000
C	-0.154371000	1.827961000	1.013591000
C	-0.365520000	3.158796000	1.347501000
C	-0.523904000	4.087541000	0.327990000
C	-0.469627000	3.661003000	-0.995056000
C	-0.263872000	2.316857000	-1.250449000
H	1.186720000	-2.393831000	1.536337000
H	1.649248000	-1.260464000	2.815008000
H	4.127379000	-1.153992000	2.109839000
H	5.752574000	-0.511981000	0.328035000
H	-1.435852000	-1.263640000	2.857073000
H	-0.900568000	-2.450588000	1.662613000
H	-3.912215000	-1.441245000	2.161130000
H	-5.589594000	-1.085753000	0.348631000
H	1.045731000	0.944033000	2.527851000
H	-0.674142000	0.831452000	2.843792000
H	-0.403227000	3.460313000	2.386885000
H	-0.689545000	5.131513000	0.562173000
H	-4.783836000	-0.554487000	-1.957041000
H	4.886226000	0.100391000	-1.934644000
H	-0.589383000	4.355452000	-1.815434000
O	0.097496000	-0.953875000	-2.304102000
H	-2.293662000	-0.349544000	-2.333731000

H	2.389476000	0.077898000	-2.303562000
H	-0.221171000	1.916365000	-2.254998000

[(MAPA)Co^{III}(O$_2$)]$^+$ (singlet)

Co	0.178557000	-0.541081000	-0.462449000
O	0.358185000	-2.147380000	-1.327164000
N	2.102303000	-0.379178000	-0.378957000
N	-1.751465000	-0.804499000	-0.178141000
N	-0.108006000	1.386479000	-0.214333000
N	0.308345000	-0.610590000	1.541118000
C	1.619717000	-1.265613000	1.788076000
C	2.628922000	-0.725165000	0.808906000
C	3.991588000	-0.658633000	1.039739000
C	4.824930000	-0.251137000	0.002261000
C	4.270285000	0.071333000	-1.228923000
C	2.894083000	-0.002940000	-1.384421000
C	-0.857830000	-1.435042000	1.950937000
C	-2.055434000	-1.079222000	1.112115000
C	-3.344918000	-1.120036000	1.581518000
C	-4.382188000	-0.899255000	0.662670000
C	-4.090298000	-0.667240000	-0.656609000
C	-2.741176000	-0.631275000	-1.080146000
C	0.262100000	0.776660000	2.096480000
C	-0.113570000	1.806112000	1.056453000
C	-0.413874000	3.120438000	1.384900000
C	-0.707920000	4.014679000	0.364539000
C	-0.699830000	3.569352000	-0.952854000
C	-0.399744000	2.242118000	-1.203452000
H	1.494022000	-2.329284000	1.579600000
H	1.948234000	-1.156261000	2.825445000
H	4.393563000	-0.934337000	2.006094000
H	5.895418000	-0.195057000	0.154188000
H	-1.064870000	-1.346558000	3.020676000
H	-0.602871000	-2.474018000	1.735768000
H	-3.547506000	-1.336803000	2.621073000
H	-5.413469000	-0.926137000	0.991939000
H	1.249740000	1.028695000	2.488077000
H	-0.433103000	0.820069000	2.937356000
H	-0.417558000	3.435367000	2.420875000
H	-0.945650000	5.045566000	0.594649000
H	-4.874711000	-0.514771000	-1.386497000
H	4.889641000	0.375103000	-2.061744000

H	-0.930017000	4.235228000	-1.773300000
O	0.244956000	-1.050829000	-2.221474000
N	-2.384644000	-0.388974000	-2.361050000
H	-3.109665000	-0.453194000	-3.057671000
H	-1.439347000	-0.720356000	-2.615260000
H	2.389538000	0.201650000	-2.318206000
H	-0.402325000	1.826477000	-2.201436000

[(MAPA)Co^{III}(O_2)]$^+$ (triplet)

+6.1 kcal mol^{-1} from singlet [(MAPA)Co^{III}(O_2)]$^+$

Co	0.282980000	-0.534865000	-0.584229000
O	0.595111000	-2.176909000	-1.456882000
N	2.383938000	-0.312807000	-0.318492000
N	-1.731472000	-1.060599000	0.025449000
N	-0.293805000	1.382604000	-0.510781000
N	0.445655000	-0.275889000	1.561139000
C	1.775866000	-0.794372000	1.955538000
C	2.825959000	-0.401393000	0.941963000
C	4.164475000	-0.228943000	1.260336000
C	5.067049000	0.017167000	0.231341000
C	4.604938000	0.080432000	-1.077396000
C	3.248501000	-0.088309000	-1.311204000
C	-0.661457000	-1.045756000	2.177180000
C	-1.926573000	-0.986002000	1.356285000
C	-3.175385000	-0.961606000	1.932544000
C	-4.287498000	-1.048132000	1.082675000
C	-4.107585000	-1.143556000	-0.274297000
C	-2.794312000	-1.139658000	-0.797637000
C	0.344238000	1.185918000	1.814268000
C	-0.366436000	1.931996000	0.711977000
C	-0.998415000	3.148149000	0.920694000
C	-1.555732000	3.812336000	-0.165187000
C	-1.472708000	3.239106000	-1.428059000
C	-0.839859000	2.014929000	-1.558554000
H	1.710887000	-1.884089000	1.958240000
H	2.056791000	-0.476664000	2.965110000
H	4.494685000	-0.296946000	2.289270000
H	6.118870000	0.152822000	0.449849000
H	-0.844658000	-0.726922000	3.207832000
H	-0.343759000	-2.089955000	2.204560000
H	-3.290387000	-0.900221000	3.006069000
H	-5.288495000	-1.043283000	1.496156000
H	1.359335000	1.586310000	1.869108000

H	-0.129070000	1.381790000	2.780411000
H	-1.053271000	3.566982000	1.917373000
H	-2.054360000	4.763182000	-0.025039000
H	-4.951399000	-1.213574000	-0.948659000
H	5.279944000	0.258502000	-1.903582000
H	-1.899162000	3.722872000	-2.296114000
O	0.313394000	-1.148156000	-2.317802000
N	-2.557999000	-1.159115000	-2.132533000
H	-3.312109000	-1.422048000	-2.744811000
H	-1.609119000	-1.353257000	-2.447169000
H	2.820538000	-0.061210000	-2.305560000
H	-0.768368000	1.504394000	-2.509666000

[(BAPA)Co^{III}(O_2)]$^+$ (singlet)

Co	0.201101000	-0.472315000	-0.521732000
O	0.398613000	-1.798331000	-1.769308000
N	2.122180000	-0.318492000	-0.392057000
N	-1.735270000	-0.837546000	-0.329526000
N	-0.083373000	1.337923000	0.247930000
N	0.334585000	-1.090290000	1.371693000
C	1.640968000	-1.799383000	1.421416000
C	2.650984000	-1.004764000	0.636235000
C	4.013759000	-1.008458000	0.875384000
C	4.844862000	-0.303002000	0.010606000
C	4.287183000	0.383141000	-1.059613000
C	2.911243000	0.355875000	-1.229900000
C	-0.833399000	-1.983429000	1.567186000
C	-2.032745000	-1.421357000	0.856192000
C	-3.318440000	-1.585636000	1.308345000
C	-4.363870000	-1.160899000	0.475706000
C	-4.081018000	-0.633137000	-0.757460000
C	-2.735393000	-0.493207000	-1.168602000
C	0.308248000	0.095178000	2.281239000
C	-0.163792000	1.352771000	1.592966000
C	-0.558292000	2.463792000	2.302489000
C	-0.855754000	3.626700000	1.583968000
C	-0.737169000	3.637372000	0.214968000
C	-0.339863000	2.461924000	-0.451710000
H	1.508093000	-2.760104000	0.921470000
H	1.969313000	-1.987456000	2.447469000
H	4.416800000	-1.560901000	1.714257000
H	5.915444000	-0.293357000	0.171551000
H	-1.030964000	-2.168743000	2.626040000

H	-0.591280000	-2.935712000	1.092049000
H	-3.510960000	-2.048928000	2.265910000
H	-5.392611000	-1.269168000	0.795981000
H	1.327045000	0.274971000	2.630559000
H	-0.299486000	-0.117759000	3.162487000
H	-0.622654000	2.436299000	3.381652000
H	-1.172740000	4.521195000	2.105501000
H	-4.870653000	-0.328160000	-1.431865000
H	4.903706000	0.934617000	-1.756144000
H	-0.946114000	4.530533000	-0.359140000
O	0.274080000	-0.513184000	-2.364203000
N	-0.150836000	2.436255000	-1.797769000
H	-0.565109000	3.176354000	-2.340632000
H	-0.111629000	1.534706000	-2.251539000
N	-2.399182000	0.007260000	-2.380025000
H	-3.132301000	0.059101000	-3.069084000
H	-1.455722000	-0.252234000	-2.703038000
H	2.408759000	0.852700000	-2.046824000

[(BAPA)Co^{III}(O$_2$)]$^+$ (triplet)

+4.6 kcal mol^{-1} from singlet [(BAPA)Co^{III}(O$_2$)]$^+$

Co	0.231528000	-0.144122000	-0.712801000
O	0.487766000	-0.844567000	-2.376160000
N	2.360496000	-0.291702000	-0.258568000
N	-1.914458000	-0.660643000	-0.731734000
N	-0.164522000	1.232919000	0.706824000
N	0.170751000	-1.506334000	0.882546000
C	1.467080000	-2.227348000	0.873915000
C	2.620592000	-1.288107000	0.601134000
C	3.886790000	-1.483464000	1.131163000
C	4.915704000	-0.635726000	0.734893000
C	4.647549000	0.375562000	-0.178668000
C	3.349176000	0.515587000	-0.648875000
C	-0.971416000	-2.415697000	0.611202000
C	-2.186085000	-1.664059000	0.121896000
C	-3.468818000	-2.034277000	0.457083000
C	-4.524598000	-1.339298000	-0.148447000
C	-4.263003000	-0.334467000	-1.047995000
C	-2.920910000	-0.009422000	-1.340118000
C	-0.012769000	-0.721261000	2.137326000
C	-0.482922000	0.694601000	1.906232000
C	-1.110491000	1.415438000	2.894036000
C	-1.393763000	2.764333000	2.646693000

C	-1.050653000	3.331950000	1.444454000
C	-0.434551000	2.532435000	0.461142000
H	1.428609000	-2.948562000	0.055189000
H	1.624274000	-2.785629000	1.801995000
H	4.067850000	-2.289926000	1.830749000
H	5.913802000	-0.767302000	1.133354000
H	-1.209725000	-3.021761000	1.490483000
H	-0.658002000	-3.089136000	-0.188861000
H	-3.652050000	-2.843631000	1.150428000
H	-5.549292000	-1.599688000	0.086366000
H	0.956990000	-0.659339000	2.636442000
H	-0.690781000	-1.241792000	2.817107000
H	-1.362452000	0.954055000	3.838985000
H	-1.884846000	3.362236000	3.404440000
H	-5.064493000	0.205436000	-1.535290000
H	5.424986000	1.045422000	-0.520171000
H	-1.264013000	4.371365000	1.233096000
O	0.380003000	0.518988000	-2.450037000
N	-0.153536000	3.023991000	-0.771497000
H	-0.198964000	4.017220000	-0.922678000
H	0.428043000	2.491324000	-1.400743000
N	-2.587758000	0.991222000	-2.198090000
H	-3.306709000	1.352475000	-2.803245000
H	-1.644120000	0.968855000	-2.575603000
H	3.081128000	1.276536000	-1.372142000

[(BAPA)Co^{III}(O$_2$)]$^+$ (singlet, 2 hydrogen bonds)

+28.3 kcal mol^{-1} from singlet [(BAPA)Co^{III}(O$_2$)]$^+$

Co	0.000159000	-0.540401000	0.489382000
O	-0.000241000	-2.004248000	1.593849000
N	-1.965985000	-0.584694000	0.292650000
N	1.966203000	-0.584912000	0.292407000
N	0.000241000	1.336264000	-0.073011000
N	-0.000243000	-0.965076000	-1.461800000
C	-1.241336000	-1.757236000	-1.655938000
C	-2.358343000	-1.100600000	-0.896361000
C	-3.661098000	-1.074393000	-1.328981000
C	-4.622046000	-0.510795000	-0.477262000
C	-4.246120000	-0.024276000	0.748401000
C	-2.888950000	-0.080179000	1.138899000
C	1.241274000	-1.756356000	-1.656807000
C	2.358252000	-1.099832000	-0.897110000
C	3.660896000	-1.073170000	-1.330064000

C	4.621995000	-0.510205000	-0.478098000
C	4.246409000	-0.024708000	0.748054000
C	2.889338000	-0.080987000	1.138877000
C	-0.001023000	0.299068000	-2.262292000
C	-0.000626000	1.536751000	-1.395750000
C	-0.001032000	2.815965000	-1.934120000
C	-0.000451000	3.904346000	-1.073044000
C	0.000593000	3.684927000	0.300266000
C	0.000925000	2.382244000	0.764772000
H	-1.064884000	-2.740334000	-1.216491000
H	-1.481464000	-1.888680000	-2.714291000
H	-3.930807000	-1.490012000	-2.289802000
H	-5.660924000	-0.471596000	-0.780243000
H	1.481192000	-1.886937000	-2.715316000
H	1.065480000	-2.739883000	-1.218059000
H	3.930466000	-1.487897000	-2.291305000
H	5.660800000	-0.470737000	-0.781315000
H	-0.878924000	0.314786000	-2.910988000
H	0.876007000	0.315112000	-2.912158000
H	-0.001736000	2.953944000	-3.008092000
H	-0.000748000	4.912295000	-1.468374000
H	4.972907000	0.394684000	1.431888000
H	-4.972393000	0.395816000	1.432052000
H	0.001129000	4.508042000	1.001607000
O	-0.000002000	-0.781579000	2.318335000
N	-2.456481000	0.390832000	2.332975000
H	-3.164450000	0.582998000	3.024096000
H	-1.587236000	-0.049611000	2.663031000
H	0.001767000	2.143243000	1.817985000
N	2.457210000	0.388913000	2.333436000
H	3.165278000	0.580857000	3.024458000
H	1.587629000	-0.051327000	2.663274000

[(TAPA)Co^{III}(O$_2$)]$^+$ (singlet)

Co	0.188261000	-0.327835000	-0.611190000
O	0.401853000	-1.176707000	-2.218929000
N	2.138573000	-0.308711000	-0.300766000
N	-1.771986000	-0.643826000	-0.651617000
N	-0.150477000	1.116531000	0.742461000
N	0.151707000	-1.574710000	0.939809000
C	1.466162000	-2.260256000	0.907186000
C	2.533884000	-1.252638000	0.585212000
C	3.816013000	-1.320954000	1.076726000

C	4.750656000	-0.392608000	0.603998000
C	4.376725000	0.526500000	-0.346339000
C	3.048856000	0.540832000	-0.815035000
C	-0.989601000	-2.478851000	0.657003000
C	-2.146083000	-1.664721000	0.156494000
C	-3.456387000	-1.965032000	0.431359000
C	-4.448390000	-1.188778000	-0.185718000
C	-4.089238000	-0.184029000	-1.044632000
C	-2.719959000	0.077053000	-1.289903000
C	-0.037752000	-0.795475000	2.200126000
C	-0.512487000	0.610941000	1.940777000
C	-1.186730000	1.343216000	2.888455000
C	-1.484317000	2.679088000	2.592451000
C	-1.069940000	3.225041000	1.402783000
C	-0.371527000	2.421107000	0.478163000
H	1.433341000	-2.983683000	0.090733000
H	1.671255000	-2.798358000	1.836384000
H	4.088809000	-2.081722000	1.794842000
H	5.769905000	-0.411449000	0.968971000
H	-1.254998000	-3.081938000	1.529300000
H	-0.676957000	-3.148247000	-0.146415000
H	-3.706702000	-2.784136000	1.090796000
H	-5.494996000	-1.391608000	0.004332000
H	0.931579000	-0.726882000	2.698711000
H	-0.714057000	-1.323079000	2.874673000
H	-1.471124000	0.897891000	3.831805000
H	-2.024613000	3.286868000	3.307558000
H	-4.834925000	0.414739000	-1.551399000
H	5.088639000	1.231961000	-0.754776000
H	-1.263509000	4.262764000	1.164360000
O	0.293242000	0.230797000	-2.374622000
N	0.074146000	2.915552000	-0.704228000
H	0.035750000	3.914555000	-0.825529000
N	2.618926000	1.414803000	-1.775431000
H	3.350202000	1.883274000	-2.289642000
H	1.845173000	1.023378000	-2.341557000
H	0.872349000	2.458608000	-1.132982000
N	-2.316291000	1.057395000	-2.123593000
H	-3.011679000	1.469426000	-2.723828000
H	-1.368752000	0.944609000	-2.496889000

$[(TAPA)Co^{III}(O_2)]^+$ (triplet)

+5.8 kcal mol^{-1} from singlet $[(TAPA)Co^{III}(O_2)]^+$

Co	0.197386000	-0.101608000	-0.743696000
O	0.400943000	-0.507251000	-2.540038000
N	2.326567000	-0.331373000	-0.395470000
N	-1.919706000	-0.638711000	-0.781128000
N	-0.153049000	1.064992000	0.864422000
N	0.183237000	-1.671386000	0.668456000
C	1.483183000	-2.373514000	0.539049000
C	2.612875000	-1.380923000	0.396140000
C	3.852414000	-1.562950000	0.969338000
C	4.846742000	-0.618598000	0.687167000
C	4.573843000	0.440096000	-0.148599000
C	3.283289000	0.558205000	-0.698377000
C	-0.965013000	-2.540796000	0.316878000
C	-2.184329000	-1.722885000	-0.028101000
C	-3.459487000	-2.098463000	0.325723000
C	-4.522656000	-1.317263000	-0.149566000
C	-4.272085000	-0.222486000	-0.938972000
C	-2.934034000	0.108281000	-1.252190000
C	0.030466000	-1.052032000	2.014518000
C	-0.501631000	0.357512000	1.964864000
C	-1.205208000	0.912055000	3.005972000
C	-1.543798000	2.269381000	2.923881000
C	-1.149854000	3.012529000	1.838991000
C	-0.430252000	2.384421000	0.802420000
H	1.438109000	-2.965483000	-0.377132000
H	1.657643000	-3.058128000	1.374537000
H	4.049075000	-2.414410000	1.606472000
H	5.834574000	-0.725477000	1.117965000
H	-1.179366000	-3.258926000	1.114148000
H	-0.677422000	-3.101471000	-0.574982000
H	-3.631819000	-2.975156000	0.934801000
H	-5.543110000	-1.580212000	0.100593000
H	1.021794000	-1.001002000	2.471095000
H	-0.590261000	-1.679121000	2.658343000
H	-1.480086000	0.315857000	3.864950000
H	-2.103464000	2.737635000	3.724083000
H	-5.078618000	0.389245000	-1.322411000
H	5.333147000	1.172156000	-0.391963000
H	-1.380251000	4.067310000	1.766308000
O	0.265164000	0.848344000	-2.354433000
N	-0.020020000	3.067210000	-0.296317000
H	-0.085755000	4.071480000	-0.278370000

N	2.926831000	1.596100000	-1.522236000
H	3.686897000	2.115884000	-1.933945000
H	2.154260000	1.393312000	-2.156056000
H	0.746532000	2.687396000	-0.834524000
N	-2.614720000	1.200842000	-1.988843000
H	-3.347152000	1.650639000	-2.512079000
H	-1.673762000	1.243978000	-2.373293000

^{1}2(MAPA)$^{+}$

Co	-0.047767000	-0.269442000	-0.489772000
O	-0.042869000	-2.126472000	-0.711251000
N	1.916224000	-0.539660000	-0.558461000
N	-1.796705000	-0.182423000	-0.077848000
N	0.092556000	1.690093000	-0.271204000
N	0.185357000	-0.355729000	1.492625000
C	1.273379000	-1.367027000	1.643754000
C	2.338926000	-1.097911000	0.597444000
C	3.670550000	-1.428372000	0.785061000
C	4.575677000	-1.205087000	-0.247406000
C	4.127326000	-0.640744000	-1.433185000
C	2.784038000	-0.316334000	-1.546476000
C	-1.159323000	-0.752019000	2.103745000
C	-2.276448000	-0.603222000	1.076571000
C	-3.603818000	-0.985863000	1.106507000
C	-4.272594000	-1.030885000	-0.138211000
C	-3.665494000	-0.747240000	-1.356175000
C	-2.321240000	-0.312678000	-1.313551000
C	0.652974000	0.985615000	1.960962000
C	0.296625000	2.083061000	0.994613000
C	0.254763000	3.420283000	1.357228000
C	0.012133000	4.372585000	0.375306000
C	-0.192200000	3.957058000	-0.934369000
C	-0.158008000	2.601831000	-1.219165000
H	0.843680000	-2.346495000	1.445704000
H	1.696455000	-1.352304000	2.652188000
H	3.992907000	-1.861826000	1.723048000
H	5.618953000	-1.466409000	-0.122894000
H	-1.335912000	-0.166244000	3.006249000
H	-1.103658000	-1.802842000	2.377948000
H	-4.098824000	-1.293372000	2.016399000
H	-5.306173000	-1.356769000	-0.146733000
H	1.742001000	0.953720000	2.033112000
H	0.275488000	1.199832000	2.962051000

H	0.410772000	3.708680000	2.388943000
H	-0.024870000	5.423808000	0.631907000
H	-4.187955000	-0.893926000	-2.291469000
H	4.800494000	-0.452731000	-2.258363000
H	-0.392347000	4.666956000	-1.725307000
O	-0.998418000	-2.860912000	0.074915000
N	-1.295001000	-0.065003000	-2.154803000
H	-1.707376000	-3.026563000	-0.561924000
H	-1.353601000	-0.516804000	-3.059257000
H	2.375427000	0.128070000	-2.444665000
H	-0.366058000	2.199975000	-2.200483000

^{3}2(MAPA)$^{+}$

-2.0 kcal mol^{-1} from ^{1}2(MAPA)$^{+}$

Co	0.079843000	-0.414255000	-0.594460000
O	0.235564000	-2.120146000	-1.190855000
N	2.052017000	-0.360052000	-0.564759000
N	-1.758062000	-0.538777000	-0.237650000
N	-0.013396000	1.507227000	-0.200513000
N	0.291423000	-0.568954000	1.536667000
C	1.511228000	-1.395735000	1.574557000
C	2.536652000	-0.870786000	0.585920000
C	3.901406000	-0.943703000	0.814181000
C	4.780111000	-0.503748000	-0.169823000
C	4.268682000	0.010494000	-1.353089000
C	2.892824000	0.069797000	-1.509050000
C	-0.969873000	-1.229177000	1.936518000
C	-2.115098000	-0.857920000	1.009667000
C	-3.445494000	-0.945828000	1.376083000
C	-4.428867000	-0.744343000	0.387634000
C	-4.067162000	-0.438369000	-0.896874000
C	-2.680750000	-0.281129000	-1.230136000
C	0.469200000	0.789753000	2.078889000
C	0.120873000	1.876975000	1.081235000
C	-0.017096000	3.203866000	1.464429000
C	-0.288195000	4.162645000	0.498264000
C	-0.426522000	3.767690000	-0.827337000
C	-0.291129000	2.426497000	-1.138244000
H	1.229246000	-2.399206000	1.251655000
H	1.949129000	-1.461531000	2.576701000
H	4.269708000	-1.345406000	1.749448000
H	5.849649000	-0.559342000	-0.010902000
H	-1.228440000	-1.014809000	2.977741000

H	-0.825107000	-2.304081000	1.836930000
H	-3.717875000	-1.201420000	2.391266000
H	-5.476044000	-0.835200000	0.649159000
H	1.514633000	0.922637000	2.369173000
H	-0.123148000	0.925649000	2.988051000
H	0.087672000	3.477662000	2.506901000
H	-0.397449000	5.203194000	0.776959000
H	-4.809303000	-0.278005000	-1.668271000
H	4.917971000	0.362935000	-2.142834000
H	-0.648723000	4.482058000	-1.608261000
O	-0.567991000	-3.061029000	-0.454276000
N	-2.162166000	0.124900000	-2.366383000
H	-0.579703000	-3.804229000	-1.073409000
H	-2.882886000	0.223032000	-3.080179000
H	2.437987000	0.464708000	-2.408314000
H	-0.437111000	2.043344000	-2.138806000

^{1}2(BAPA)$^{+}$

Co	-0.155486000	-0.403124000	-0.487692000
O	-0.389924000	-1.878567000	-1.459886000
N	1.764679000	-0.812084000	-0.589133000
N	-1.970334000	-0.087620000	-0.141572000
N	0.390048000	1.358055000	0.191469000
N	-0.082150000	-1.002598000	1.332704000
C	1.053927000	-1.970389000	1.386205000
C	2.170305000	-1.517916000	0.480060000
C	3.502385000	-1.846119000	0.662693000
C	4.425028000	-1.448052000	-0.299517000
C	3.991200000	-0.726768000	-1.404372000
C	2.644447000	-0.416596000	-1.512677000
C	-1.428896000	-1.645491000	1.545916000
C	-2.461307000	-0.741244000	0.932672000
C	-3.763024000	-0.627299000	1.326650000
C	-4.607152000	0.170189000	0.509188000
C	-4.125554000	0.803017000	-0.597075000
C	-2.733435000	0.704911000	-0.964746000
C	0.093172000	0.183144000	2.243052000
C	0.676830000	1.355557000	1.506628000
C	1.389602000	2.364257000	2.108344000
C	1.813890000	3.429952000	1.301015000
C	1.510245000	3.449094000	-0.039112000
C	0.776779000	2.382370000	-0.600763000
H	0.676948000	-2.918987000	1.002558000

H	1.392590000	-2.120963000	2.413040000
H	3.811053000	-2.406975000	1.535335000
H	5.472427000	-1.696406000	-0.183667000
H	-1.595728000	-1.846149000	2.605926000
H	-1.423563000	-2.583294000	0.990608000
H	-4.135371000	-1.146168000	2.198216000
H	-5.653849000	0.272095000	0.770285000
H	0.690856000	-0.098402000	3.110652000
H	-0.902263000	0.461704000	2.593157000
H	1.608260000	2.335529000	3.166744000
H	2.377605000	4.245339000	1.737119000
H	-4.775233000	1.400920000	-1.223836000
H	4.682053000	-0.400624000	-2.169656000
H	1.819845000	4.269571000	-0.673085000
O	-1.693028000	-2.475094000	-1.458964000
N	0.461575000	2.270413000	-1.913366000
H	0.661425000	3.083048000	-2.478750000
H	-0.491075000	1.833582000	-2.080878000
N	-2.123905000	1.273653000	-1.972357000
H	-1.472336000	-3.360180000	-1.783713000
H	-2.773287000	1.806557000	-2.542231000
H	2.241368000	0.169015000	-2.328528000

^{3}2(BAPA)$^{+}$

-2.4 kcal mol^{-1} from ^{1}2(BAPA)$^{+}$

Co	-0.162261000	-0.374112000	-0.562892000
O	-0.473714000	-1.834609000	-1.535932000
N	1.760502000	-0.804729000	-0.629495000
N	-1.968066000	-0.082097000	-0.150255000
N	0.406631000	1.360980000	0.208418000
N	-0.048951000	-1.026105000	1.449948000
C	1.048837000	-2.005019000	1.370814000
C	2.148318000	-1.535523000	0.434428000
C	3.478820000	-1.880768000	0.609084000
C	4.413833000	-1.479260000	-0.338458000
C	3.997022000	-0.729581000	-1.430482000
C	2.655207000	-0.402206000	-1.537025000
C	-1.412652000	-1.573666000	1.657329000
C	-2.425779000	-0.681929000	0.969394000
C	-3.731666000	-0.563674000	1.366049000
C	-4.613612000	0.155420000	0.522854000
C	-4.165701000	0.729457000	-0.629549000

C	-2.773897000	0.652653000	-0.991426000
C	0.228224000	0.151999000	2.300939000
C	0.726473000	1.345545000	1.515935000
C	1.430445000	2.370734000	2.106176000
C	1.807045000	3.457264000	1.308664000
C	1.471283000	3.484644000	-0.024797000
C	0.754758000	2.405790000	-0.576075000
H	0.633708000	-2.921757000	0.947615000
H	1.465078000	-2.246380000	2.354259000
H	3.776329000	-2.459356000	1.474134000
H	5.456961000	-1.744186000	-0.220113000
H	-1.648298000	-1.703769000	2.717242000
H	-1.450510000	-2.549121000	1.173305000
H	-4.080391000	-1.042045000	2.270617000
H	-5.657974000	0.243567000	0.797506000
H	0.934333000	-0.099596000	3.096658000
H	-0.706982000	0.443852000	2.783567000
H	1.682659000	2.330048000	3.157032000
H	2.360547000	4.280838000	1.742619000
H	-4.835548000	1.275456000	-1.281527000
H	4.695856000	-0.394644000	-2.184671000
H	1.744479000	4.319483000	-0.656701000
O	-1.612293000	-2.617999000	-1.215848000
N	0.425275000	2.307386000	-1.892179000
H	0.599551000	3.135602000	-2.442877000
H	-0.505989000	1.862886000	-2.074973000
N	-2.185138000	1.214276000	-2.017875000
H	-1.754820000	-3.082512000	-2.054231000
H	-2.859844000	1.668891000	-2.626358000
H	2.266435000	0.205377000	-2.343840000

^{1}2(TAPA)$^{+}$

Co	-0.167483000	-0.407642000	-0.441915000
O	-0.274056000	-1.846489000	-1.477088000
N	1.824108000	-0.744583000	-0.406468000
N	-2.031584000	-0.191034000	-0.186034000
N	0.213742000	1.382155000	0.329322000
N	-0.165483000	-1.052055000	1.381457000
C	0.985002000	-2.000556000	1.463545000
C	2.160038000	-1.488885000	0.674302000
C	3.454719000	-1.839210000	0.964665000
C	4.456260000	-1.437090000	0.069441000
C	4.128981000	-0.698869000	-1.038187000

C	2.782127000	-0.330345000	-1.260499000
C	-1.497582000	-1.743126000	1.517325000
C	-2.536908000	-0.878398000	0.864240000
C	-3.858870000	-0.843491000	1.201634000
C	-4.715289000	-0.095962000	0.353542000
C	-4.222195000	0.571643000	-0.726259000
C	-2.812158000	0.566841000	-1.027494000
C	-0.054327000	0.104842000	2.337036000
C	0.434705000	1.354243000	1.656320000
C	0.992374000	2.414825000	2.331927000
C	1.313342000	3.560556000	1.594563000
C	1.054467000	3.609644000	0.243716000
C	0.484314000	2.491277000	-0.391893000
H	0.653183000	-2.929398000	0.999050000
H	1.246382000	-2.205322000	2.503448000
H	3.684339000	-2.427501000	1.842121000
H	5.488915000	-1.705495000	0.254040000
H	-1.709377000	-1.968503000	2.564121000
H	-1.428153000	-2.672184000	0.951337000
H	-4.234334000	-1.391811000	2.053708000
H	-5.776817000	-0.060188000	0.567761000
H	0.579929000	-0.171749000	3.179725000
H	-1.057098000	0.302335000	2.720212000
H	1.167006000	2.362758000	3.397728000
H	1.754881000	4.415998000	2.090517000
H	-4.876664000	1.134677000	-1.379749000
H	4.887063000	-0.375361000	-1.739462000
H	1.275422000	4.494974000	-0.337896000
O	-1.535683000	-2.517641000	-1.557363000
N	0.212892000	2.412611000	-1.726816000
H	0.321901000	3.285372000	-2.225842000
N	2.420987000	0.412552000	-2.329642000
H	3.154975000	0.804051000	-2.896620000
H	1.552298000	0.942590000	-2.311873000
H	-0.717608000	1.908432000	-1.934500000
N	-2.208740000	1.202006000	-2.001250000
H	-1.246038000	-3.377871000	-1.895207000
H	-2.875683000	1.694362000	-2.586817000

^{3}2(TAPA)$^{+}$

-5.6 kcal mol^{-1} from ^{1}2(TAPA)$^{+}$

Co	0.111477000	-0.467470000	-0.391343000
O	0.293146000	-2.105216000	-1.188785000

N	2.044084000	-0.536849000	-0.183191000
N	-1.779305000	-0.569586000	-0.257207000
N	0.033412000	1.474145000	0.140851000
N	0.054270000	-0.755725000	1.729946000
C	1.353647000	-1.405568000	1.964847000
C	2.437553000	-0.823503000	1.074487000
C	3.757461000	-0.700437000	1.453093000
C	4.690748000	-0.315921000	0.484844000
C	4.295748000	-0.127834000	-0.824227000
C	2.940492000	-0.283157000	-1.142527000
C	-1.142015000	-1.610283000	1.816186000
C	-2.222211000	-1.117923000	0.871544000
C	-3.579260000	-1.317694000	1.105540000
C	-4.495689000	-0.978329000	0.101406000
C	-4.043490000	-0.439959000	-1.077587000
C	-2.645233000	-0.196852000	-1.264049000
C	-0.091920000	0.551273000	2.391975000
C	-0.325087000	1.693612000	1.424901000
C	-0.811155000	2.902054000	1.867946000
C	-0.912756000	3.951158000	0.947922000
C	-0.490945000	3.764921000	-0.344281000
C	0.012945000	2.506080000	-0.732335000
H	1.250592000	-2.451806000	1.671229000
H	1.656781000	-1.377111000	3.016277000
H	4.059799000	-0.912694000	2.469504000
H	5.733331000	-0.200920000	0.753843000
H	-1.526191000	-1.694730000	2.838712000
H	-0.866722000	-2.605188000	1.461539000
H	-3.909128000	-1.753782000	2.039025000
H	-5.554253000	-1.146144000	0.255879000
H	0.828519000	0.771196000	2.939412000
H	-0.894909000	0.519597000	3.132930000
H	-1.099710000	3.031049000	2.902423000
H	-1.305304000	4.911892000	1.257411000
H	-4.727778000	-0.176204000	-1.873770000
H	5.012516000	0.111240000	-1.598841000
H	-0.522242000	4.569066000	-1.067829000
O	-0.880333000	-2.960793000	-1.079713000
N	0.539073000	2.323259000	-1.966102000
H	0.262410000	2.951670000	-2.701307000
N	2.422925000	-0.172468000	-2.421831000
H	3.111874000	-0.103451000	-3.157230000

H	1.713698000	-0.875095000	-2.618749000
H	0.825893000	1.398556000	-2.254966000
N	-2.080686000	0.381417000	-2.303288000
H	-0.591380000	-3.707252000	-1.620975000
H	-2.779597000	0.570679000	-3.022617000

^{1}3(MAPA)$^{+}$

Co	0.053058000	-0.494909000	-0.467180000
O	-0.455084000	-0.221013000	-2.269519000
N	1.999006000	-0.438837000	-0.515128000
N	-1.745680000	-0.653112000	-0.152011000
N	-0.035770000	1.444999000	-0.061113000
N	0.259683000	-0.734712000	1.492431000
C	1.532661000	-1.506648000	1.600502000
C	2.532816000	-0.945400000	0.613753000
C	3.904952000	-0.996200000	0.791955000
C	4.733294000	-0.539699000	-0.228807000
C	4.167533000	-0.036548000	-1.392655000
C	2.785143000	0.004544000	-1.495740000
C	-0.971080000	-1.504609000	1.899927000
C	-2.149159000	-1.079339000	1.048196000
C	-3.494190000	-1.214961000	1.301475000
C	-4.381565000	-0.934432000	0.240557000
C	-3.937580000	-0.550058000	-1.008089000
C	-2.546472000	-0.414217000	-1.193246000
C	0.355269000	0.597210000	2.167808000
C	0.037147000	1.744106000	1.241134000
C	-0.117682000	3.045993000	1.695491000
C	-0.340000000	4.055394000	0.768371000
C	-0.400754000	3.735006000	-0.583149000
C	-0.249075000	2.412374000	-0.962182000
H	1.311363000	-2.523743000	1.280301000
H	1.921708000	-1.513096000	2.621540000
H	4.318479000	-1.395208000	1.709206000
H	5.809205000	-0.577545000	-0.113801000
H	-1.158526000	-1.420690000	2.971754000
H	-0.775686000	-2.544422000	1.641267000
H	-3.857951000	-1.552204000	2.261645000
H	-5.446362000	-1.043193000	0.405625000
H	1.375242000	0.728168000	2.535008000
H	-0.298810000	0.621249000	3.041138000
H	-0.061134000	3.263329000	2.754710000
H	-0.464894000	5.079419000	1.097222000

H	-4.626694000	-0.362325000	-1.820038000
H	4.780956000	0.321693000	-2.208010000
H	-0.570997000	4.494528000	-1.333988000
N	-1.862205000	-0.009073000	-2.291873000
H	-2.245018000	-0.215415000	-3.204775000
O	0.156553000	-2.333147000	-0.659953000
H	-0.103336000	-2.523627000	-1.568230000
H	2.273225000	0.384487000	-2.370728000
H	-0.297870000	2.074480000	-1.988168000

^{3}3(MAPA)$^+$

+12.5 kcal mol^{-1} from ^{1}3(MAPA)$^+$

Co	-0.051214000	-0.562868000	-0.641432000
O	-0.734029000	0.140715000	-2.365451000
N	1.907782000	-0.672313000	-0.598926000
N	-1.883184000	-0.561948000	-0.200739000
N	0.160470000	1.398840000	0.010765000
N	0.129511000	-0.821834000	1.579599000
C	1.365674000	-1.625523000	1.594238000
C	2.382683000	-1.133097000	0.575295000
C	3.749012000	-1.223235000	0.793917000
C	4.627066000	-0.856832000	-0.219828000
C	4.118203000	-0.401977000	-1.428762000
C	2.743133000	-0.318961000	-1.577795000
C	-1.140462000	-1.537996000	1.858721000
C	-2.274443000	-0.969455000	1.018729000
C	-3.601765000	-0.913871000	1.366209000
C	-4.517492000	-0.451350000	0.392851000
C	-4.105361000	-0.042297000	-0.852637000
C	-2.719886000	-0.083956000	-1.135764000
C	0.238028000	0.471590000	2.266056000
C	0.334640000	1.643630000	1.313183000
C	0.567245000	2.930072000	1.785723000
C	0.615766000	3.982594000	0.885359000
C	0.428582000	3.719733000	-0.467180000
C	0.204430000	2.414953000	-0.866130000
H	1.097347000	-2.636968000	1.290450000
H	1.821260000	-1.664483000	2.589030000
H	4.118874000	-1.584942000	1.744762000
H	5.696485000	-0.926495000	-0.065381000
H	-1.398011000	-1.525572000	2.921506000
H	-0.996340000	-2.574089000	1.553797000
H	-3.936348000	-1.227490000	2.345111000

H	-5.572314000	-0.417044000	0.635984000
H	1.102655000	0.479541000	2.936360000
H	-0.641310000	0.621976000	2.897585000
H	0.706165000	3.096411000	2.846949000
H	0.796236000	4.992361000	1.232355000
H	-4.807621000	0.314524000	-1.594073000
H	4.768553000	-0.112885000	-2.242887000
H	0.455988000	4.510346000	-1.204713000
N	-2.092695000	0.363547000	-2.237634000
H	-2.584345000	0.488130000	-3.110862000
O	-0.049249000	-2.350390000	-0.855410000
H	-0.943968000	-2.686534000	-0.987817000
H	2.278861000	0.031886000	-2.490511000
H	0.043318000	2.128015000	-1.896659000

$^{1}3(BAPA)^{+}$

Co	0.080266000	-0.492134000	-0.484109000
O	-0.448168000	-0.026524000	-2.265253000
N	2.029349000	-0.418650000	-0.502399000
N	-1.726068000	-0.721428000	-0.214134000
N	-0.003839000	1.407446000	0.249281000
N	0.276240000	-1.054101000	1.395824000
C	1.546495000	-1.839388000	1.391441000
C	2.553402000	-1.122245000	0.521434000
C	3.923184000	-1.201425000	0.706174000
C	4.763122000	-0.559572000	-0.198171000
C	4.209231000	0.156987000	-1.250095000
C	2.828450000	0.211815000	-1.364026000
C	-0.951853000	-1.874327000	1.686733000
C	-2.127452000	-1.339361000	0.901454000
C	-3.469200000	-1.556198000	1.115515000
C	-4.359158000	-1.156274000	0.099920000
C	-3.915036000	-0.590205000	-1.078482000
C	-2.530646000	-0.385413000	-1.221841000
C	0.376971000	0.148633000	2.274680000
C	-0.073306000	1.416156000	1.598194000
C	-0.392195000	2.535069000	2.330382000
C	-0.610091000	3.731686000	1.636549000
C	-0.474884000	3.760021000	0.272523000
C	-0.154805000	2.573156000	-0.424221000
H	1.329287000	-2.793084000	0.913893000
H	1.923531000	-2.004613000	2.403520000
H	4.325458000	-1.760709000	1.540962000

H	5.837567000	-0.614918000	-0.076851000
H	-1.140710000	-1.939628000	2.759431000
H	-0.757324000	-2.869401000	1.288778000
H	-3.824742000	-2.045087000	2.011474000
H	-5.420888000	-1.320977000	0.235269000
H	1.429081000	0.282669000	2.534666000
H	-0.163111000	-0.016740000	3.207983000
H	-0.446265000	2.490992000	3.409532000
H	-0.869903000	4.634407000	2.175369000
H	-4.600417000	-0.317033000	-1.869057000
H	4.830279000	0.672250000	-1.969926000
H	-0.612531000	4.676520000	-0.286514000
N	0.062968000	2.572896000	-1.759791000
H	-0.245060000	3.384640000	-2.270637000
H	-0.035534000	1.661063000	-2.224672000
N	-1.863966000	0.172345000	-2.271020000
H	-2.233700000	-0.002532000	-3.196796000
O	0.190558000	-2.279155000	-0.953682000
H	-0.041343000	-2.317140000	-1.888539000
H	2.333859000	0.768132000	-2.148771000

^{3}3(BAPA)$^{+}$

+12.9 kcal mol^{-1} from ^{1}3(BAPA)$^{+}$

Co	-0.268122000	-0.407743000	-0.724264000
O	-0.933786000	0.854309000	-2.193604000
N	1.647276000	-0.868036000	-0.796380000
N	-2.095985000	-0.236714000	-0.200299000
N	0.499797000	1.271917000	0.441371000
N	-0.171598000	-1.271989000	1.325310000
C	0.969417000	-2.190120000	1.160633000
C	2.044256000	-1.641793000	0.233555000
C	3.379177000	-1.985173000	0.380379000
C	4.304362000	-1.542497000	-0.557696000
C	3.873836000	-0.758441000	-1.618907000
C	2.529322000	-0.435215000	-1.700674000
C	-1.512613000	-1.894630000	1.433032000
C	-2.551873000	-0.957022000	0.844819000
C	-3.859597000	-0.846050000	1.245322000
C	-4.706644000	0.009885000	0.506948000
C	-4.240561000	0.724594000	-0.568925000
C	-2.875954000	0.591646000	-0.912871000
C	0.019087000	-0.202924000	2.315503000
C	0.712339000	1.011731000	1.750667000

C	1.458508000	1.824859000	2.571977000
C	2.008992000	2.991054000	2.028230000
C	1.809797000	3.274650000	0.703600000
C	1.059523000	2.383753000	-0.099068000
H	0.589296000	-3.094839000	0.685002000
H	1.413170000	-2.466719000	2.122373000
H	3.687357000	-2.597563000	1.217992000
H	5.349732000	-1.806167000	-0.457610000
H	-1.765257000	-2.166528000	2.462017000
H	-1.499123000	-2.800670000	0.827673000
H	-4.229896000	-1.406249000	2.092470000
H	-5.746785000	0.102384000	0.793890000
H	0.554050000	-0.570558000	3.196803000
H	-0.970869000	0.113316000	2.652891000
H	1.602246000	1.566665000	3.612260000
H	2.592727000	3.660937000	2.647471000
H	-4.886129000	1.378556000	-1.139827000
H	4.562298000	-0.395230000	-2.369451000
H	2.231467000	4.161742000	0.248640000
N	0.931451000	2.591510000	-1.427204000
H	1.215493000	3.488730000	-1.784475000
H	0.180887000	2.078598000	-1.916860000
N	-2.225345000	1.265001000	-1.881581000
H	-2.738857000	1.620267000	-2.676528000
O	-0.583694000	-2.018684000	-1.440530000
H	-1.522503000	-2.146557000	-1.627697000
H	2.132403000	0.192290000	-2.486499000

$^{1}3(TAPA)^{+}$

Co	-0.081489000	-0.484773000	0.417449000
O	0.342611000	0.024791000	2.219946000
N	-2.048046000	-0.391913000	0.315879000
N	1.731434000	-0.780460000	0.284111000
N	0.190566000	1.405432000	-0.393426000
N	-0.176269000	-1.089813000	-1.452052000
C	-1.452272000	-1.860181000	-1.506271000
C	-2.507513000	-1.080757000	-0.759893000
C	-3.837909000	-1.109506000	-1.100941000
C	-4.745208000	-0.432415000	-0.274900000
C	-4.296068000	0.218436000	0.846164000
C	-2.916300000	0.213712000	1.144489000
C	1.057446000	-1.921967000	-1.667854000
C	2.187096000	-1.429128000	-0.792479000

C	3.528828000	-1.718675000	-0.897101000
C	4.355384000	-1.360286000	0.183374000
C	3.847872000	-0.765067000	1.321894000
C	2.470738000	-0.487309000	1.352499000
C	-0.242816000	0.088733000	-2.361188000
C	0.321011000	1.331643000	-1.739021000
C	0.812616000	2.352042000	-2.516159000
C	1.162418000	3.550952000	-1.882057000
C	0.993797000	3.669946000	-0.528888000
C	0.498855000	2.574818000	0.218227000
H	-1.287231000	-2.789042000	-0.963530000
H	-1.749858000	-2.071809000	-2.535878000
H	-4.168837000	-1.652124000	-1.975479000
H	-5.801687000	-0.434064000	-0.512390000
H	1.322657000	-1.969807000	-2.725204000
H	0.819427000	-2.923686000	-1.311473000
H	3.926828000	-2.234247000	-1.759705000
H	5.414409000	-1.580815000	0.133498000
H	-1.298030000	0.282306000	-2.564800000
H	0.234912000	-0.137530000	-3.315822000
H	0.903298000	2.232700000	-3.586976000
H	1.555433000	4.379843000	-2.457519000
H	4.480566000	-0.522976000	2.164778000
H	-4.980788000	0.727112000	1.512253000
H	1.238880000	4.588134000	-0.010750000
N	0.286563000	2.687991000	1.545852000
H	0.717807000	3.463639000	2.020464000
N	-2.423748000	0.836196000	2.245457000
H	-3.084362000	1.144697000	2.939212000
H	-1.517056000	0.549480000	2.592905000
H	0.190102000	1.813799000	2.061007000
N	1.761928000	0.106867000	2.359075000
H	2.030344000	-0.137052000	3.304236000
O	-0.257838000	-2.251200000	0.937163000
H	-0.109650000	-2.258442000	1.889524000

^{3}3(TAPA)$^{+}$

+13.5 kcal mol^{-1} from ^{1}3(TAPA)$^{+}$

Co	-0.118236000	-0.593639000	0.558681000
O	0.504146000	0.145317000	2.355733000
N	-2.066678000	-0.476763000	0.326092000
N	1.734913000	-0.876114000	0.356536000
N	0.156253000	1.404607000	-0.397179000

N	-0.138685000	-1.098622000	-1.594195000
C	-1.443885000	-1.795623000	-1.637476000
C	-2.486463000	-1.026387000	-0.844324000
C	-3.798990000	-0.947711000	-1.242963000
C	-4.717763000	-0.316816000	-0.392161000
C	-4.304826000	0.188135000	0.814488000
C	-2.944861000	0.082324000	1.176307000
C	1.076808000	-1.941584000	-1.697055000
C	2.183070000	-1.444113000	-0.779453000
C	3.531783000	-1.615051000	-0.973223000
C	4.403303000	-1.212852000	0.060914000
C	3.931689000	-0.647574000	1.222398000
C	2.538031000	-0.461395000	1.347882000
C	-0.109818000	0.099828000	-2.442797000
C	0.358124000	1.346567000	-1.732140000
C	0.839760000	2.399993000	-2.475862000
C	1.091603000	3.611401000	-1.824780000
C	0.844485000	3.713060000	-0.481997000
C	0.371667000	2.588094000	0.232940000
H	-1.316138000	-2.758325000	-1.144315000
H	-1.789945000	-1.960689000	-2.661876000
H	-4.110521000	-1.376488000	-2.185242000
H	-5.758510000	-0.239723000	-0.681199000
H	1.435041000	-2.018384000	-2.727326000
H	0.804993000	-2.941887000	-1.358116000
H	3.910525000	-2.059400000	-1.882985000
H	5.470094000	-1.352742000	-0.062451000
H	-1.130245000	0.296521000	-2.780996000
H	0.490812000	-0.071737000	-3.340145000
H	0.990104000	2.291842000	-3.541508000
H	1.471229000	4.462467000	-2.376623000
H	4.599436000	-0.335507000	2.014123000
H	-5.001816000	0.658496000	1.495647000
H	1.014694000	4.638913000	0.052403000
N	0.082819000	2.688462000	1.549676000
H	0.419594000	3.511749000	2.022075000
N	-2.478742000	0.562956000	2.356129000
H	-3.152828000	0.841597000	3.049373000
H	-1.593652000	0.226947000	2.712603000
H	0.101025000	1.812761000	2.081939000
N	1.898813000	0.167196000	2.359782000
H	2.299784000	0.158606000	3.287378000

O	-0.369842000	-2.314550000	1.016213000
H	0.461124000	-2.726739000	1.285213000

^{1}4(MAPA)$^{+}$ - Note: This is a transition structure

Co	0.113855000	-0.383345000	-0.349899000
O	-0.298364000	-0.872346000	-2.049844000
N	2.045474000	-0.199544000	-0.430895000
N	-1.525166000	-0.997506000	-0.008082000
N	-0.359000000	1.464026000	-0.340795000
N	0.328791000	-0.197997000	1.629440000
C	1.650333000	-0.844101000	1.865016000
C	2.613875000	-0.427740000	0.773092000
C	3.984558000	-0.345455000	0.945239000
C	4.784322000	-0.048931000	-0.154871000
C	4.189319000	0.165130000	-1.391484000
C	2.808825000	0.088747000	-1.488585000
C	-0.852777000	-0.918088000	2.247884000
C	-1.997416000	-0.997505000	1.257219000
C	-3.331041000	-1.179174000	1.479702000
C	-4.181031000	-1.351244000	0.341035000
C	-3.696650000	-1.335114000	-0.936865000
C	-2.296059000	-1.139888000	-1.132115000
C	0.358769000	1.260098000	1.944533000
C	-0.298751000	2.068941000	0.855939000
C	-0.769784000	3.359715000	1.032126000
C	-1.305881000	4.032848000	-0.059070000
C	-1.369715000	3.390777000	-1.290016000
C	-0.896833000	2.093854000	-1.394804000
H	1.497952000	-1.919138000	1.766773000
H	2.047882000	-0.627060000	2.860820000
H	4.420762000	-0.519220000	1.920530000
H	5.859478000	0.014212000	-0.044611000
H	-1.140125000	-0.451735000	3.191655000
H	-0.523060000	-1.936274000	2.458860000
H	-3.736048000	-1.220857000	2.481620000
H	-5.240867000	-1.499823000	0.505425000
H	1.403817000	1.570337000	2.010305000
H	-0.097597000	1.461160000	2.915923000
H	-0.721777000	3.825601000	2.008104000
H	-1.680699000	5.042190000	0.054022000
H	-4.340615000	-1.451447000	-1.797452000
H	4.780343000	0.392140000	-2.268280000
H	-1.793312000	3.877031000	-2.158140000

N	-1.645639000	-1.049399000	-2.283208000
O	0.908361000	-3.061182000	-0.377802000
H	0.528597000	-2.889354000	-1.250647000
H	2.277417000	0.242717000	-2.419269000
H	-0.954151000	1.518618000	-2.309128000
H	1.182363000	-3.984843000	-0.387119000

^{3}4(MAPA)$^{+}$

-12.6 kcal mol^{-1} from ^{1}4(MAPA)$^{+}$

Co	0.244976000	-0.513804000	-0.389415000
O	-0.342010000	-0.882075000	-2.182246000
N	2.166650000	-0.211435000	-0.460534000
N	-1.473358000	-0.988580000	-0.006395000
N	-0.379051000	1.556954000	-0.464195000
N	0.417001000	-0.144373000	1.574171000
C	1.763597000	-0.701760000	1.881378000
C	2.721595000	-0.311905000	0.770198000
C	4.074971000	-0.099083000	0.967030000
C	4.876801000	0.207964000	-0.128962000
C	4.298887000	0.306551000	-1.387534000
C	2.934016000	0.095797000	-1.511976000
C	-0.739208000	-0.851489000	2.237538000
C	-1.891055000	-1.020096000	1.259588000
C	-3.221071000	-1.273920000	1.534957000
C	-4.082362000	-1.510140000	0.442218000
C	-3.624552000	-1.495648000	-0.860924000
C	-2.260353000	-1.219951000	-1.085709000
C	0.385676000	1.333004000	1.798436000
C	-0.457069000	2.057370000	0.774974000
C	-1.203496000	3.187444000	1.068945000
C	-1.881208000	3.823209000	0.032605000
C	-1.798171000	3.301888000	-1.251430000
C	-1.041662000	2.155191000	-1.455795000
H	1.671452000	-1.787678000	1.880687000
H	2.137320000	-0.380169000	2.858245000
H	4.496381000	-0.171877000	1.961419000
H	5.938371000	0.374721000	0.002999000
H	-1.044646000	-0.334988000	3.149837000
H	-0.391585000	-1.845337000	2.523330000
H	-3.589558000	-1.302284000	2.551242000
H	-5.128565000	-1.713032000	0.634231000
H	1.410098000	1.695384000	1.693143000
H	0.063977000	1.559790000	2.817738000

H	-1.255721000	3.563893000	2.082939000
H	-2.473119000	4.708121000	0.230259000
H	-4.278033000	-1.684509000	-1.701178000
H	4.889577000	0.548466000	-2.260612000
H	-2.318646000	3.763138000	-2.080080000
N	-1.642203000	-1.175166000	-2.297393000
O	0.801296000	-2.837975000	-0.148817000
H	1.394750000	-3.062040000	-0.876070000
H	2.417737000	0.165764000	-2.461101000
H	-0.966930000	1.675205000	-2.423894000
H	-0.016764000	-3.307827000	-0.353220000

^{1}4(BAPA)$^{+}$

Co	0.143342000	-0.460110000	-0.457811000
O	-0.387158000	-0.190412000	-2.230549000
N	2.089695000	-0.222807000	-0.443740000
N	-1.578706000	-0.988123000	-0.257007000
N	-0.200311000	1.342200000	0.185807000
N	0.371326000	-0.987353000	1.452784000
C	1.696346000	-1.662998000	1.468346000
C	2.654678000	-0.874017000	0.596471000
C	4.021544000	-0.828400000	0.810509000
C	4.818820000	-0.110004000	-0.075369000
C	4.225533000	0.555871000	-1.139667000
C	2.849290000	0.481828000	-1.287333000
C	-0.818628000	-1.856113000	1.790746000
C	-2.002416000	-1.430885000	0.948699000
C	-3.333186000	-1.561703000	1.202028000
C	-4.236210000	-1.209272000	0.142333000
C	-3.805981000	-0.764009000	-1.071131000
C	-2.392296000	-0.623485000	-1.305979000
C	0.385074000	0.263153000	2.261228000
C	-0.263808000	1.410049000	1.540981000
C	-0.746873000	2.499638000	2.216503000
C	-1.154542000	3.612380000	1.466698000
C	-1.026130000	3.592850000	0.105506000
C	-0.526046000	2.440137000	-0.548647000
H	1.569019000	-2.652196000	1.028077000
H	2.086908000	-1.783218000	2.482567000
H	4.455717000	-1.346867000	1.655631000
H	5.890588000	-0.065572000	0.071226000
H	-1.024467000	-1.834669000	2.862209000
H	-0.548152000	-2.880026000	1.523953000

H	-3.698510000	-1.950076000	2.142053000
H	-5.300273000	-1.307586000	0.319402000
H	1.428155000	0.539036000	2.430784000
H	-0.068191000	0.095802000	3.239430000
H	-0.788225000	2.499792000	3.296812000
H	-1.554608000	4.487728000	1.962774000
H	-4.495425000	-0.494283000	-1.858970000
H	4.813212000	1.128860000	-1.843736000
H	-1.308498000	4.446035000	-0.497227000
N	-0.315455000	2.436438000	-1.878305000
H	-0.756849000	3.171696000	-2.407719000
H	-0.266248000	1.512477000	-2.328313000
N	-1.816085000	-0.169863000	-2.385790000
O	0.547842000	-2.391381000	-1.248050000
H	0.404798000	-2.025115000	-2.146349000
H	2.321856000	0.988690000	-2.084880000
H	-0.240790000	-2.936753000	-1.105815000

^{3}4(BAPA)$^{+}$

-11.3 kcal mol^{-1} from ^{1}4(BAPA)$^{+}$

Co	0.242273000	-0.494836000	-0.390021000
O	-0.368366000	-0.618291000	-2.217197000
N	2.166555000	-0.204758000	-0.448512000
N	-1.464694000	-1.046811000	-0.062244000
N	-0.320910000	1.616355000	-0.085338000
N	0.460138000	-0.482673000	1.593719000
C	1.796884000	-1.117411000	1.767500000
C	2.743178000	-0.549588000	0.726570000
C	4.104860000	-0.404862000	0.928380000
C	4.891691000	0.092994000	-0.106241000
C	4.290967000	0.446334000	-1.307134000
C	2.919810000	0.288669000	-1.437284000
C	-0.696675000	-1.260147000	2.169601000
C	-1.851351000	-1.315484000	1.184364000
C	-3.158490000	-1.696610000	1.430190000
C	-4.027613000	-1.813106000	0.327720000
C	-3.594655000	-1.577099000	-0.963395000
C	-2.254910000	-1.186234000	-1.155192000
C	0.480586000	0.933320000	2.069624000
C	-0.386128000	1.846221000	1.238828000
C	-1.094757000	2.886829000	1.795020000
C	-1.755449000	3.762058000	0.921419000
C	-1.679521000	3.561227000	-0.434205000

C	-0.948988000	2.455624000	-0.927348000
H	1.679610000	-2.183195000	1.572979000
H	2.193175000	-0.983198000	2.778521000
H	4.543326000	-0.674781000	1.880476000
H	5.959195000	0.211326000	0.030075000
H	-1.001217000	-0.846334000	3.132692000
H	-0.356026000	-2.281563000	2.347155000
H	-3.499785000	-1.911814000	2.433572000
H	-5.055338000	-2.108687000	0.497635000
H	1.508566000	1.286447000	1.969210000
H	0.219605000	0.983734000	3.129188000
H	-1.126235000	3.030359000	2.866551000
H	-2.323948000	4.596016000	1.314218000
H	-4.247331000	-1.687753000	-1.818032000
H	4.868928000	0.844778000	-2.129824000
H	-2.175957000	4.227511000	-1.128192000
N	-0.817149000	2.227186000	-2.260714000
H	-1.456194000	2.699481000	-2.879547000
H	-0.549813000	1.288632000	-2.540892000
N	-1.662694000	-0.959140000	-2.358166000
O	0.790217000	-2.840469000	-0.583040000
H	1.342915000	-2.907729000	-1.371312000
H	2.388975000	0.556485000	-2.341789000
H	-0.034010000	-3.276577000	-0.831962000

^{1}4(TAPA)$^{+}$ - Note: This is a transition structure

Co	0.032986000	-0.354871000	-0.234934000
O	-0.311413000	-0.711619000	-2.001998000
N	1.998961000	-0.361416000	-0.212949000
N	-1.648553000	-0.937956000	-0.036006000
N	-0.270439000	1.554886000	0.009365000
N	0.164853000	-0.458854000	1.741944000
C	1.387794000	-1.290412000	1.928254000
C	2.463244000	-0.796618000	0.987903000
C	3.801531000	-0.822497000	1.293751000
C	4.710272000	-0.409264000	0.308384000
C	4.254551000	-0.008558000	-0.922669000
C	2.866916000	-0.005590000	-1.179540000
C	-1.107584000	-1.091009000	2.266499000
C	-2.175000000	-1.124671000	1.192292000
C	-3.497958000	-1.434643000	1.325860000
C	-4.281986000	-1.549515000	0.138278000
C	-3.738872000	-1.389562000	-1.104702000

C	-2.347181000	-1.087459000	-1.206637000
C	0.376468000	0.927861000	2.239251000
C	-0.243338000	1.934237000	1.313472000
C	-0.662188000	3.166419000	1.744954000
C	-1.123073000	4.079294000	0.786453000
C	-1.145442000	3.720618000	-0.534028000
C	-0.719381000	2.428948000	-0.925211000
H	1.127571000	-2.306855000	1.631794000
H	1.727660000	-1.289116000	2.967785000
H	4.138421000	-1.159416000	2.264180000
H	5.773274000	-0.416033000	0.514596000
H	-1.443508000	-0.569132000	3.163187000
H	-0.871148000	-2.117366000	2.551254000
H	-3.936185000	-1.620322000	2.296948000
H	-5.334726000	-1.785099000	0.232139000
H	1.453039000	1.109801000	2.254544000
H	0.011480000	1.041733000	3.261509000
H	-0.624176000	3.420235000	2.795177000
H	-1.463181000	5.062109000	1.087652000
H	-4.326108000	-1.483838000	-2.007317000
H	4.939907000	0.295369000	-1.703146000
H	-1.498434000	4.404578000	-1.294812000
N	-0.726783000	2.070881000	-2.223715000
H	-1.223948000	2.657309000	-2.872812000
N	2.352555000	0.377705000	-2.375157000
H	2.996896000	0.494086000	-3.139981000
H	1.433219000	0.027805000	-2.622298000
H	-0.625235000	1.093273000	-2.470702000
N	-1.654204000	-0.937669000	-2.321700000
O	0.505032000	-3.159936000	-0.556609000
H	0.214412000	-2.840043000	-1.420753000
H	0.663825000	-4.103627000	-0.664734000

^{3}4(TAPA)$^{+}$ - Note: This is a transition structure

-11.6 kcal mol^{-1} from ^{1}4(TAPA)$^{+}$

Co	0.172108000	-0.471147000	-0.225386000
O	-0.266300000	-0.819282000	-2.069233000
N	2.112645000	-0.268089000	-0.181501000
N	-1.547008000	-1.024416000	0.014439000
N	-0.440285000	1.644727000	-0.136459000
N	0.245087000	-0.284393000	1.746540000
C	1.534542000	-0.962796000	2.060068000
C	2.585975000	-0.503774000	1.069946000

C	3.913496000	-0.367159000	1.393220000
C	4.802487000	0.005996000	0.373756000
C	4.339971000	0.218501000	-0.900763000
C	2.962382000	0.065974000	-1.170893000
C	-0.983591000	-0.947484000	2.320987000
C	-2.023643000	-1.197809000	1.243383000
C	-3.320750000	-1.653307000	1.419876000
C	-4.077905000	-1.947353000	0.272637000
C	-3.542860000	-1.818892000	-0.996449000
C	-2.220144000	-1.354729000	-1.114400000
C	0.316856000	1.166713000	2.087394000
C	-0.512418000	2.012810000	1.156818000
C	-1.210152000	3.115250000	1.595763000
C	-1.854958000	3.902696000	0.632218000
C	-1.779528000	3.556073000	-0.693503000
C	-1.061742000	2.395548000	-1.062600000
H	1.375987000	-2.032595000	1.919953000
H	1.854456000	-0.786807000	3.091408000
H	4.257996000	-0.544839000	2.402453000
H	5.856879000	0.122735000	0.591140000
H	-1.389605000	-0.348589000	3.137819000
H	-0.687697000	-1.909786000	2.742529000
H	-3.734125000	-1.791806000	2.409764000
H	-5.094745000	-2.300930000	0.388173000
H	1.358811000	1.467854000	1.966151000
H	0.045278000	1.329403000	3.133105000
H	-1.245888000	3.368903000	2.646509000
H	-2.412293000	4.781720000	0.931590000
H	-4.102140000	-2.075056000	-1.885329000
H	5.012280000	0.498154000	-1.701429000
H	-2.265919000	4.149874000	-1.456935000
N	-0.944269000	2.021089000	-2.362999000
H	-1.580031000	2.424290000	-3.031015000
N	2.442650000	0.278118000	-2.404699000
H	3.081146000	0.388474000	-3.174522000
H	1.533480000	-0.109763000	-2.626800000
H	-0.643692000	1.072491000	-2.547859000
N	-1.534188000	-1.247264000	-2.284295000
O	0.664471000	-2.949093000	-0.442454000
H	0.480456000	-2.938019000	-1.390163000
H	0.916578000	-3.853673000	-0.229543000

^{1}5(MAPA)$^{+}$

Co	0.111057000	-0.502905000	-0.447348000
O	-0.352872000	-0.711987000	-2.184167000
N	2.051700000	-0.471710000	-0.531630000
N	-1.548160000	-1.080215000	-0.159413000
N	-0.209932000	1.372960000	-0.198366000
N	0.348855000	-0.574221000	1.543816000
C	1.625839000	-1.327287000	1.688681000
C	2.611605000	-0.852099000	0.639672000
C	3.984178000	-0.854270000	0.813276000
C	4.797581000	-0.479098000	-0.252933000
C	4.213037000	-0.104906000	-1.456017000
C	2.830512000	-0.105836000	-1.555622000
C	-0.876768000	-1.280231000	2.092286000
C	-2.021898000	-1.189546000	1.103524000
C	-3.364381000	-1.309970000	1.312881000
C	-4.228330000	-1.303964000	0.172631000
C	-3.745105000	-1.165174000	-1.098876000
C	-2.338999000	-1.024770000	-1.277737000
C	0.483838000	0.829247000	2.036802000
C	-0.115825000	1.812936000	1.066039000
C	-0.494646000	3.099622000	1.412359000
C	-0.972113000	3.947589000	0.420235000
C	-1.067368000	3.479790000	-0.884911000
C	-0.687174000	2.177162000	-1.159367000
H	1.402596000	-2.378109000	1.493994000
H	2.043498000	-1.250197000	2.697107000
H	4.411665000	-1.152217000	1.762051000
H	5.874440000	-0.479685000	-0.142077000
H	-1.138879000	-0.890679000	3.077086000
H	-0.611313000	-2.332431000	2.210529000
H	-3.769765000	-1.431759000	2.308383000
H	-5.294098000	-1.409692000	0.330007000
H	1.548807000	1.054192000	2.126360000
H	0.048333000	0.937960000	3.032007000
H	-0.419935000	3.429058000	2.440895000
H	-1.275221000	4.957460000	0.665756000
H	-4.394758000	-1.139886000	-1.962698000
H	4.814149000	0.187451000	-2.306195000
H	-1.443967000	4.106078000	-1.682113000
N	-1.687024000	-0.809569000	-2.416133000
H	2.307064000	0.173802000	-2.461185000
H	-0.770148000	1.737102000	-2.143624000

^{3}5(MAPA)$^{+}$

-8.5 kcal mol^{-1} from ^{1}5(MAPA)$^{+}$

Co	0.212084000	-0.542546000	-0.410925000
O	-0.336242000	-0.907909000	-2.188553000
N	2.147942000	-0.459821000	-0.480446000
N	-1.493569000	-1.053834000	-0.032747000
N	-0.305890000	1.496966000	-0.435831000
N	0.404067000	-0.250691000	1.554632000
C	1.687831000	-0.948479000	1.848204000
C	2.688632000	-0.648094000	0.746124000
C	4.057138000	-0.611124000	0.942912000
C	4.888315000	-0.396023000	-0.153802000
C	4.325577000	-0.216588000	-1.410338000
C	2.945088000	-0.250282000	-1.534340000
C	-0.814719000	-0.844423000	2.232914000
C	-1.920837000	-1.119404000	1.226153000
C	-3.232550000	-1.473698000	1.486334000
C	-4.060864000	-1.770738000	0.385677000
C	-3.590019000	-1.723168000	-0.913441000
C	-2.248515000	-1.351595000	-1.120475000
C	0.527286000	1.220794000	1.792557000
C	-0.280345000	2.019531000	0.797538000
C	-0.891551000	3.225762000	1.098179000
C	-1.538706000	3.914226000	0.075689000
C	-1.560393000	3.369950000	-1.201549000
C	-0.937786000	2.147333000	-1.414897000
H	1.482389000	-2.020857000	1.843314000
H	2.089590000	-0.688166000	2.832358000
H	4.468683000	-0.751994000	1.934064000
H	5.962759000	-0.367489000	-0.024109000
H	-1.157538000	-0.185889000	3.032442000
H	-0.520629000	-1.786131000	2.699690000
H	-3.609322000	-1.531652000	2.498395000
H	-5.090684000	-2.052530000	0.565990000
H	1.577683000	1.481782000	1.649502000
H	0.266092000	1.470394000	2.823536000
H	-0.862939000	3.621127000	2.105818000
H	-2.025739000	4.859669000	0.278906000
H	-4.216149000	-1.965125000	-1.760721000
H	4.941284000	-0.049429000	-2.283563000
H	-2.060078000	3.873324000	-2.018304000
N	-1.615111000	-1.274225000	-2.322209000

H	2.438838000	-0.116539000	-2.481759000
H	-0.944157000	1.648896000	-2.376584000

^{1}5(BAPA)$^{+}$

Co	0.083218000	-0.447866000	-0.480190000
O	-0.431528000	-0.400669000	-2.235606000
N	2.051816000	-0.491587000	-0.573641000
N	-1.584746000	-1.057154000	-0.261621000
N	-0.081329000	1.414459000	0.158641000
N	0.349579000	-0.928832000	1.471365000
C	1.583250000	-1.763887000	1.434535000
C	2.589089000	-1.140109000	0.485956000
C	3.959292000	-1.254705000	0.640862000
C	4.795094000	-0.706781000	-0.328086000
C	4.234982000	-0.050554000	-1.415900000
C	2.854693000	0.044005000	-1.499751000
C	-0.895875000	-1.662909000	1.926238000
C	-2.039290000	-1.433966000	0.957884000
C	-3.369143000	-1.677012000	1.142642000
C	-4.248397000	-1.525561000	0.027065000
C	-3.788461000	-1.153040000	-1.204281000
C	-2.395275000	-0.901445000	-1.360087000
C	0.576471000	0.344677000	2.214508000
C	-0.032815000	1.527207000	1.508857000
C	-0.399576000	2.668001000	2.176587000
C	-0.814184000	3.770365000	1.415590000
C	-0.830120000	3.687496000	0.048442000
C	-0.454501000	2.481228000	-0.587976000
H	1.299785000	-2.740565000	1.037332000
H	2.012394000	-1.912597000	2.429981000
H	4.366904000	-1.768344000	1.502052000
H	5.870014000	-0.789635000	-0.229776000
H	-1.154827000	-1.375081000	2.945853000
H	-0.662437000	-2.729280000	1.936459000
H	-3.749557000	-2.004824000	2.100811000
H	-5.303920000	-1.720602000	0.167394000
H	1.654777000	0.513322000	2.255023000
H	0.222431000	0.265750000	3.243798000
H	-0.353547000	2.712720000	3.255922000
H	-1.116486000	4.687180000	1.905983000
H	-4.444697000	-1.034454000	-2.055103000
H	4.852765000	0.387799000	-2.187738000
H	-1.135289000	4.528314000	-0.560691000

N	-0.419627000	2.385348000	-1.934086000
H	-0.866786000	3.110574000	-2.470316000
H	-0.375267000	1.463072000	-2.361072000
N	-1.775569000	-0.516927000	-2.467434000
H	2.356817000	0.549175000	-2.317001000

^{3}5(BAPA)$^{+}$

-9.0 kcal mol^{-1} from ^{1}5(BAPA)$^{+}$

Co	0.136726000	-0.412709000	-0.398165000
O	-0.431412000	-0.650532000	-2.197445000
N	2.080570000	-0.495867000	-0.502483000
N	-1.537954000	-1.046917000	-0.044148000
N	-0.213201000	1.669374000	-0.128151000
N	0.396046000	-0.392662000	1.567046000
C	1.559302000	-1.307159000	1.719920000
C	2.589906000	-0.981847000	0.653175000
C	3.948659000	-1.186966000	0.812323000
C	4.799929000	-0.901408000	-0.251650000
C	4.268025000	-0.411965000	-1.437450000
C	2.898348000	-0.218331000	-1.524371000
C	-0.878893000	-0.867087000	2.231790000
C	-1.878083000	-1.365477000	1.205290000
C	-3.050770000	-2.060311000	1.445898000
C	-3.843648000	-2.417598000	0.339496000
C	-3.449929000	-2.128503000	-0.953733000
C	-2.232280000	-1.449336000	-1.141351000
C	0.740441000	1.003425000	1.968450000
C	-0.057133000	2.004246000	1.169538000
C	-0.516333000	3.182724000	1.708073000
C	-1.158161000	4.084707000	0.845265000
C	-1.308285000	3.773514000	-0.481771000
C	-0.823621000	2.533139000	-0.961342000
H	1.196238000	-2.326057000	1.563363000
H	1.995521000	-1.256766000	2.722698000
H	4.336075000	-1.565026000	1.749617000
H	5.866567000	-1.057395000	-0.151514000
H	-1.310869000	-0.019359000	2.765331000
H	-0.653798000	-1.634342000	2.975361000
H	-3.348455000	-2.322897000	2.451979000
H	-4.769774000	-2.953080000	0.506345000
H	1.796623000	1.152174000	1.737938000
H	0.613929000	1.143268000	3.044654000
H	-0.376181000	3.408343000	2.756404000

H	-1.534523000	5.026072000	1.226032000
H	-4.028124000	-2.435869000	-1.813771000
H	4.900239000	-0.179262000	-2.283435000
H	-1.795527000	4.456501000	-1.165849000
N	-0.921539000	2.190180000	-2.266296000
H	-1.523312000	2.728737000	-2.866022000
H	-0.735517000	1.228530000	-2.533441000
N	-1.651604000	-1.198431000	-2.343502000
H	2.419527000	0.159486000	-2.418232000

^{1}5(TAPA)$^{+}$

Co	-0.030069000	-0.441950000	0.402851000
O	0.368026000	-0.330788000	2.190018000
N	-2.016491000	-0.550541000	0.392963000
N	1.653082000	-1.048489000	0.317690000
N	0.191929000	1.394925000	-0.303654000
N	-0.170512000	-0.995014000	-1.530736000
C	-1.380080000	-1.866286000	-1.532220000
C	-2.468195000	-1.208103000	-0.709865000
C	-3.802392000	-1.299788000	-1.017966000
C	-4.723594000	-0.710487000	-0.137767000
C	-4.283259000	-0.080809000	0.998752000
C	-2.897742000	-0.021509000	1.265211000
C	1.118147000	-1.700863000	-1.903060000
C	2.188706000	-1.460995000	-0.856167000
C	3.526224000	-1.717018000	-0.940662000
C	4.326514000	-1.535625000	0.228503000
C	3.785756000	-1.124804000	1.413376000
C	2.385306000	-0.864711000	1.466701000
C	-0.409050000	0.240980000	-2.328453000
C	0.179966000	1.450609000	-1.660508000
C	0.577526000	2.554092000	-2.370904000
C	0.993894000	3.684675000	-1.654263000
C	0.991439000	3.657535000	-0.285986000
C	0.588955000	2.485211000	0.397182000
H	-1.100282000	-2.805165000	-1.050044000
H	-1.723620000	-2.089104000	-2.546423000
H	-4.128879000	-1.818311000	-1.908661000
H	-5.784152000	-0.762880000	-0.349656000
H	1.443181000	-1.390268000	-2.896509000
H	0.907752000	-2.771141000	-1.948792000
H	3.969964000	-2.075716000	-1.859663000
H	5.388225000	-1.738255000	0.167546000

H	-1.489557000	0.389885000	-2.382204000
H	-0.043636000	0.126004000	-3.350426000
H	0.554769000	2.547512000	-3.451822000
H	1.316310000	4.575178000	-2.179057000
H	4.383064000	-0.982066000	2.302960000
H	-4.978742000	0.362425000	1.699510000
H	1.304978000	4.516696000	0.292578000
N	0.566433000	2.452257000	1.744237000
H	1.031204000	3.190426000	2.245718000
N	-2.399413000	0.582875000	2.371353000
H	-3.050134000	0.841055000	3.094999000
H	-1.474310000	0.310076000	2.684751000
H	0.478835000	1.558997000	2.214692000
N	1.699823000	-0.450161000	2.521283000

^{3}5(TAPA)$^{+}$

-9.8 kcal mol^{-1} from ^{1}5(TAPA)$^{+}$

Co	0.079772000	-0.435993000	-0.288250000
O	-0.357825000	-0.695439000	-2.123334000
N	2.033824000	-0.572714000	-0.250355000
N	-1.622298000	-1.049922000	-0.056540000
N	-0.318603000	1.655691000	-0.073062000
N	0.185814000	-0.378245000	1.685152000
C	1.331727000	-1.291457000	1.948511000
C	2.454605000	-0.967337000	0.982244000
C	3.782805000	-1.085603000	1.303763000
C	4.727576000	-0.806417000	0.302746000
C	4.316195000	-0.437406000	-0.952861000
C	2.933994000	-0.334367000	-1.226413000
C	-1.137635000	-0.835010000	2.260328000
C	-2.066217000	-1.331884000	1.168703000
C	-3.268798000	-1.998938000	1.330288000
C	-3.979838000	-2.368475000	0.174137000
C	-3.480380000	-2.116480000	-1.090104000
C	-2.239075000	-1.462531000	-1.195605000
C	0.509638000	1.021682000	2.086002000
C	-0.231512000	2.012229000	1.225439000
C	-0.709970000	3.203295000	1.719002000
C	-1.295076000	4.096463000	0.809580000
C	-1.376866000	3.762816000	-0.518187000
C	-0.879462000	2.510632000	-0.948899000
H	0.983370000	-2.310542000	1.762504000
H	1.671186000	-1.233950000	2.987117000

H	4.088030000	-1.387706000	2.295841000
H	5.785102000	-0.889410000	0.520289000
H	-1.598932000	0.017541000	2.760692000
H	-0.971908000	-1.602321000	3.019287000
H	-3.649292000	-2.232425000	2.315476000
H	-4.926654000	-2.882892000	0.279495000
H	1.578981000	1.161294000	1.919588000
H	0.316210000	1.177791000	3.150074000
H	-0.625023000	3.443854000	2.769932000
H	-1.682136000	5.047507000	1.153818000
H	-3.995808000	-2.432318000	-1.986221000
H	5.031180000	-0.230440000	-1.738419000
H	-1.820265000	4.437428000	-1.239405000
N	-0.919220000	2.146416000	-2.252406000
H	-1.495297000	2.674244000	-2.885802000
N	2.462473000	0.034173000	-2.440314000
H	3.114856000	0.072814000	-3.206038000
H	1.506100000	-0.203823000	-2.680748000
H	-0.730816000	1.179374000	-2.489064000
N	-1.567758000	-1.249394000	-2.356908000

References

(1) Prasanth, C. P.; Ebbin, J.; Abhijith, A.; Niar, D. S.; Ibnusaud, I.; Raskatov, J.; Singaram, B.; Stabilization of $NaBH_4$ in Methanol Using a Catalytic Amount of NaOMe. Reduction of Esters and Lactones at Room Temperature without Solvent-Induced Loss of Hydride. *J Org Chem* **2018,** *83* (3), 1431-1440.

(2) Peterson, R. L.; Himes, R. A.; Kotani, H.; Suenobu, T.; Tian, L.; Siegler, M. A.; Solomon, E. I.; Fukuzumi, S.; Karlin, K. D., Cupric superoxo-mediated intermolecular C-H activation chemistry. *J Am Chem Soc* **2011,** *133* (6), 1702-5.

(3) Mareque-Rivas, J. C.; Prabaharan, R.; de Rosales, R. T., Relative importance of hydrogen bonding and coordinating groups in modulating the zinc-water acidity. *Chem Commun (Camb)* **2004,** (1), 76-7.

(4) Mikata, Y.; Nodomi, Y.; Ohnishi, R.; Kizu, A.; Konno, H., Tris(8-methoxy-2-quinolylmethyl)amine (8-MeOTQA) as a highly fluorescent $Zn^{(2+)}$ probe prepared by convenient C_3-symmetric tripodal amine synthesis. *Dalton Trans* **2015,** *44* (17), 8021-30.

(5) (a) Carpenter, J. E.; McNary, C. P.; Furin, A.; Sweeney, A. F.; Armentrout, P. B. How Hot are Your Ions Really? A Threshold Collision-Induced Dissociation Study of Substituted Benzylpyridinium “Thermometer” Ions. *J. Am. Soc. Mass Spectrom*. **2017**, *28*, 1876–1888. (b) Rahrt, R.; Auth, T.; Demireva, M.; Armentrout, P. B.; Koszlowski, K. Benzhydrylpyridinium Ions: A New Class of Thermometer Ions for the Characterization of Electrospray-Ionization Mass Spectrometers. *Anal. Chem.* **2019**, *91*, 11703-11711
